# Detailed analysis of the plasma extracellular vesicle proteome after separation from lipoproteins

**DOI:** 10.1007/s00018-018-2773-4

**Published:** 2018-02-13

**Authors:** Nasibeh Karimi, Aleksander Cvjetkovic, Su Chul Jang, Rossella Crescitelli, Mohammad Ali Hosseinpour Feizi, Rienk Nieuwland, Jan Lötvall, Cecilia Lässer

**Affiliations:** 1 Krefting Research Centre, Institute of Medicine at Sahlgrenska Academy, University of Gothenburg, Gothenburg, Sweden; 2 Department of Animal Biology, Faculty of Natural Sciences, University of Tabriz, Tabriz, Iran; 3 Codiak BioSciences, Cambridge, MA 02139 USA; 4 Laboratory of Experimental Clinical Chemistry, Department of Clinical Chemistry, and Vesicle Observation Centre, Academic Medical Centre of the University of Amsterdam, Amsterdam, The Netherlands

**Keywords:** Exosomes, Extracellular vesicles, Lipoproteins, Plasma, Serum, Size-exclusion chromatography, Density cushion, Mass spectrometry, Proteomics

## Abstract

The isolation of extracellular vesicles (EVs) from blood is of great importance to understand the biological role of circulating EVs and to develop EVs as biomarkers of disease. Due to the concurrent presence of lipoprotein particles, however, blood is one of the most difficult body fluids to isolate EVs from. The aim of this study was to develop a robust method to isolate and characterise EVs from blood with minimal contamination by plasma proteins and lipoprotein particles. Plasma and serum were collected from healthy subjects, and EVs were isolated by size-exclusion chromatography (SEC), with most particles being present in fractions 8–12, while the bulk of the plasma proteins was present in fractions 11–28. Vesicle markers peaked in fractions 7–11; however, the same fractions also contained lipoprotein particles. The purity of EVs was improved by combining a density cushion with SEC to further separate lipoprotein particles from the vesicles, which reduced the contamination of lipoprotein particles by 100-fold. Using this novel isolation procedure, a total of 1187 proteins were identified in plasma EVs by mass spectrometry, of which several proteins are known as EV-associated proteins but have hitherto not been identified in the previous proteomic studies of plasma EVs. This study shows that SEC alone is unable to completely separate plasma EVs from lipoprotein particles. However, combining SEC with a density cushion significantly improved the separation of EVs from lipoproteins and allowed for a detailed analysis of the proteome of plasma EVs, thus making blood a viable source for EV biomarker discovery.

**Electronic supplementary material:**

The online version of this article (10.1007/s00018-018-2773-4) contains supplementary material, which is available to authorized users.

## Introduction

A blood sample is minimally invasive and is one of the most commonly used samples for diagnostic purposes [1]. Diseased cells, such as tumour cells, and injured or stressed tissues release molecules into the bloodstream, and these molecules can be used to monitor the status of different tissues and organs without obtaining an invasive biopsy, and blood samples are, therefore, often referred to as a “liquid biopsy” [2]. Cells and molecules that can be analysed in a liquid biopsy include circulating tumour cells, cell-free DNA and RNA, soluble proteins, and extracellular vesicles (EVs) [2].

EVs are small (40–800 nm) membrane-enclosed vesicles that are released by all cells into the extracellular space [3, 4], and they contain RNA, lipids, proteins, and DNA that can be shuttled to other cells to influence the recipient cell’s phenotype [4–7]. Furthermore, patients suffering from diseases such as cancer have higher concentrations of circulating EVs, and these EVs can carry disease-specific molecules [3, 8–10]. Isolation of EVs from plasma and serum is, therefore, of great importance in the use of EVs as biomarkers for diseases such as cancer [3]. However, human plasma and serum contain a vast array of particles, including EVs, but also a dominating pool of lipid particles such as chylomicrons and multiple types of lipoprotein particles and plasma proteins, making blood one of the most difficult body fluids to isolate EVs from.

Chylomicrons are produced after ingestion of fat-containing meals, and they transport lipids and cholesterol to the liver via the peripheral blood plasma. The size of chylomicrons varies with the amount of ingested fat, ranging from 75 to 1200 nm in diameter [11]. The liver transforms the fat in chylomicrons into very low-density lipoproteins (VLDL, 30–80 nm), which in turn can be converted into smaller types of lipoproteins (5–35 nm). These types include intermediate-density lipoprotein (IDL), low-density lipoprotein (LDL), and high-density lipoprotein (HDL), and all of these transport triglycerides and cholesterol to and from the peripheral tissues. Lipoprotein particles are very abundant in the circulation, and estimates have suggested that there are 20- to 100-fold more lipoproteins than EVs in isolates from plasma [12, 13], with chylomicrons further increasing in numbers after food intake [13]. In addition, it is suggested that > 70% of the particles isolated from plasma are non-EVs [14] and that EVs isolated from plasma are contaminated by HDL [12] and LDL [13].

Thus, a major hurdle in the characterisation of circulating EVs is that EVs are difficult to separate from lipoproteins and chylomicrons, not only because of these molecules’ abundance, but also because they resemble EVs in their physical features, including size and density. The aim of this study was, therefore, to develop a robust procedure for isolating EVs from human blood with minimal contamination of lipoprotein particles to enable the proteome of highly purified plasma EVs to be determined.

## Materials and methods

### Blood collection and processing

**Fig. 1:**
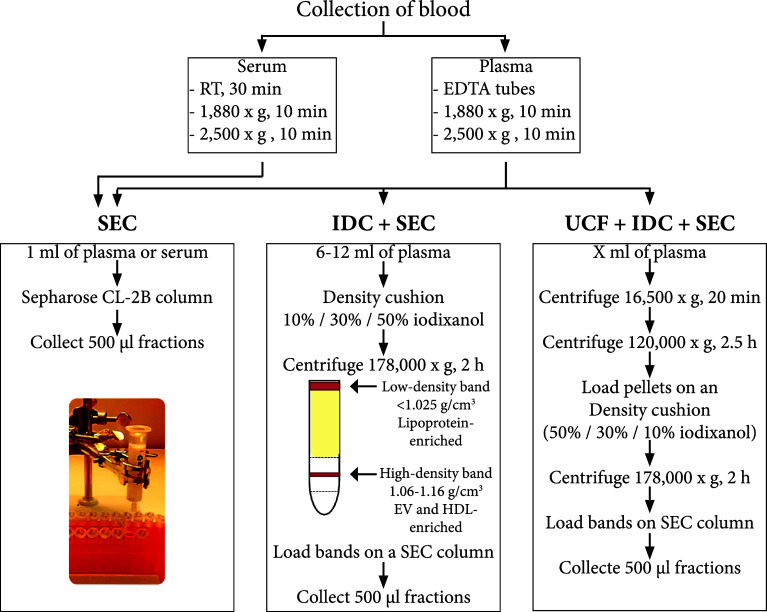
Schematic overview of the experimental workflow. Blood was collected in the morning from overnight-fasting healthy subjects, and plasma and serum were isolated. Several approaches were used to isolate EVs from plasma and serum and to separate them from lipoprotein particles and plasma proteins. *HDL* high-density lipoprotein, *IDC* iodixanol density cushion, *RT* room temperature, *SEC* size exclusion chromatography, *UCF* ultracentrifugation

Peripheral blood was collected from healthy donors after overnight-fasting. Briefly, for plasma, the blood was collected into K2E EDTA tubes, while for serum, the blood was collected into clot activator tubes. For plasma, processing was carried out directly, while, for serum, the sample was left at room temperature (RT) for 30 min to allow clotting. Plasma and serum were isolated by centrifugation at 1880×*g* for 10 min at RT. The plasma and serum were transferred to new tubes and centrifuged at 2500×*g* for 10 min at RT to minimise contamination by platelets. EVs were isolated directly by several different methods (Fig. 1). For some of the validating experiments, frozen plasma was used. Samples were collected with the approval of the Regional Ethical Approval Committee in Gothenburg, Sweden (no. 593-08).

### Isolation of EVs by size-exclusion chromatography

EVs were isolated with an in-house made size-exclusion chromatography (SEC) column as previously described [15]. Briefly, Sepharose CL-2B (GE Healthcare, Uppsala, Sweden) was packed in a Telos SPE column (Kinesis, Cambridgeshire, UK) to a final volume of 10 mL and equilibrated with PBS. Fresh plasma or serum samples (1 mL) were applied to the premade column, and up to 30 fractions of 0.5 mL were collected with PBS as the elution buffer.

### Isolation of EVs by an iodixanol density cushion and size-exclusion chromatography

An OptiPrep cushion was used for the removal of lipoprotein before loading the samples onto an SEC column. In brief, 6 mL of plasma was layered on top of a 2 mL 50%, 2 mL 30%, and 2 mL 10% OptiPrep cushion. The cushion and sample were centrifuged at 178,000×*g*_avg_ (SW 41 Ti rotor, *k*-factor 143.9, Beckman Coulter, Brea, CA, USA) for 2 h at 4 °C. A visible band between the 10 and 30% layers was collected (high-density band) as well as a band that was floating on top of the plasma (low-density band) (Fig. 1). Collected bands were loaded onto separate SEC columns. If the collected bands were less than 1 mL in volume, PBS was added to a final volume of 1 mL prior to loading. For some experiments, the same bands from two cushions were mixed to a final volume of 1 mL and loaded to one common SEC column. The SEC columns and vesicle isolation procedure were as described above.

### Isolation of EVs by ultracentrifugation, followed by iodixanol density cushion and size-exclusion chromatography

A combination of ultracentrifugation, iodixanol density cushion, and SEC was used for isolation of larger numbers of EVs of higher purity. Briefly, 40–80 mL of plasma pooled from several individuals was diluted in PBS and centrifuged at 16,500×*g*_avg_ (Type 70 Ti rotor, *k*-factor 950.6, Beckman Coulter) for 20 min to pellet larger EVs such as microvesicles. The supernatant was subjected to ultracentrifugation at 118,000×*g*_avg_ (Type 70 Ti rotor, *k*-factor 133.7, Beckman Coulter) for 2.5 h to pellet smaller EVs such as exosomes. Both EV pellets were re-suspended in PBS and mixed into one sample with a final volume of 6 mL that was loaded onto an iodixanol cushion (as described above). Again, the fraction between the 10 and 30% layer was collected and loaded onto an SEC column, and fractions were collected as described above.

### Nanoparticle tracking analysis

The particle concentrations in the SEC fractions were measured with a ZetaView^®^ PMX 110 instrument according to the manufacturer’s instruction (Particle Metrix, Meerbusch, Germany). Aliquots from the SEC fractions were diluted 10- to 1000-fold in PBS. All samples were measured in duplicate and using the same instrument settings. The chamber temperature was automatically measured and integrated into the calculation, and the sensitivity of the camera was set to 80 and the shutter was set to 100. Data were analysed using the ZetaView^®^ analysis software version 8.2.30.1 with a minimum size of 5, a maximum size of 5000, and a minimum brightness of 20.

### Protein concentration measurement

The protein concentrations in the SEC fractions were measured with a BCA assay according to the manufacturer’s instruction (Pierce^™^ BCA Protein Assay Kit, Thermo Scientific). An aliquot from each fraction was mixed with 1% SDS Tris–HCl and sonicated three times for 5 min with 10 s intermediate-speed vortexing in-between sonication prior to analysis.

### PageBlue protein staining

An equal volume (40 µL) from each of the collected SEC fractions was boiled in reducing buffer and subjected to electrophoresis on precast Mini-Protean TGX 4–20% gradient gels (Bio-Rad Laboratory). Gels were incubated with PageBlue Protein Staining Solution (Thermo Scientific, Waltham, MA, USA) for 2 h with gentle agitation at RT and then washed three times in dH_2_O before being detected with a VersaDoc 4000 MP imaging system (Bio-Rad Laboratories, Hercules, CA, USA).

### Western blot

Forty microliters of each SEC fraction were loaded and separated on Mini-Protean TGX precast 4–20% gels (Bio-Rad Laboratory), and proteins were blotted onto PVDF membranes using a Trans-Blot Turbo Transfer system (Bio-Rad Laboratory). Membranes were blocked with 5% non-fat dry milk in TBS containing 0.01% Tween-20 (TBST). Membranes were incubated with the following primary antibodies: CD81 (1:500 dilution; clone H-121, sc-9158, Santa Cruz Biotechnology, Santa Cruz, CA), TSG-101 (1:500 dilution; clone 4A10, ab83, Abcam, Cambridge, UK), flotillin-1 (1:1000 dilution; clone H-104, sc-25506, Santa Cruz Biotechnology), and Apo-A (1:1000 dilution; clone FL-267, sc-30089, Santa Cruz Biotechnology), diluted in TBST overnight at 4 °C. Membranes were washed three times before being incubated with the following secondary antibodies diluted in TBST; donkey anti-rabbit IgG HRP-linked F(ab′)_2_ fragment (1:10,000 dilution; NA9340V), and sheep anti-mouse IgG HRP-linked F(ab′)_2_ fragment (1:10,000 dilutions; NA9310V) (both from GE Healthcare, Buckinghamshire, UK). Blots were visualised with SuperSignal™ West Femto Maximum Sensitivity Substrate (Thermo Scientific) and a VersaDoc 4000 MP imaging system (Bio-Rad Laboratory) with Quantity One software.

### In-house made ELISA

Based on the protein concentration in fraction 10 (the fraction with the most particles), a volume equivalent to 500 ng proteins was used for ELISA. The same volume as for fraction 10 was then used for all other fractions, which were diluted in 1 mL PBS. Samples (100 µL per well) were added to a black-walled 96-well plate (Thermo Scientific) and incubated overnight at 4 °C. After incubation, the plate was washed three times with PBS and blocked with PBS with 1% bovine serum albumin (BSA) for 1 h at RT. The PBS-1% BSA was discarded, and the primary antibodies were added (1:200 dilution) and the plate and samples were incubated for 2 h at RT. Primary antibodies included CD9, CD63, and CD81 (all from Santa Cruz Biotechnology). The plates were then washed three times with PBS-1% BSA and then incubated with donkey anti-rabbit IgG HRP-linked F(ab′)_2_ fragment (1:2000 dilution) or sheep anti-mouse IgG HRP-linked F(ab′)_2_ fragment (1:2000 dilution) (both from GE Healthcare) for 1 h at RT. The plates and samples were then washed four times, and the BM Chemiluminescence ELISA substrate (Roche, Basel, Switzerland) was used according to the manufacturer’s protocols to measure the chemiluminescence on a Varioskan™ LUX multimode microplate reader (Thermo Fisher Scientific).

### Total RNA isolation

RNA was isolated either from 300 µL of the high-density or low-density band from the density cushion or from the pellets of pooled SEC fractions (F1–6, F7–12, F13–18, F19–24, and F25–30). The pooled fractions were ultracentrifuged at 115,000×*g*_avg_ for 1 h (TLA-100.3 rotor, *k*-factor 52.8, Beckman Coulter). Total RNA was extracted using the miRCURY RNA Isolation Kit—Cell and Plant (Exiqon, Vedbaek, Denmark) according to the manufacturer’s instructions. For the density bands, 700 µL lysis buffer was added to 300 µL sample, and for the pooled SEC fractions, 350 µL lysis buffer was added to the pellets. One microliter of isolated RNA was examined by capillary electrophoresis using an Agilent RNA 6000 Pico chip on an Agilent 2100 Bioanalyzer (Agilent Technologies, Santa Clara, CA).

### Electron microscopy

Formvar/carbon-coated copper grids (Ted Pella, Inc., Redding, CA, USA) were glow discharged before the samples were loaded. The grids and samples were incubated for 15 min, fixed sequentially in 2% paraformaldehyde and 2.5% glutaraldehyde, and contrasted in 2% uranyl acetate. The preparations were examined using an LEO 912AB Omega electron microscope (Carl Zeiss NTS, Jena, Germany).

### Mass spectrometry

Proteomic analyses were performed at The Proteomics Core Facility at the Sahlgrenska Academy, University of Gothenburg. The EV samples (30 µg) were lysed by the addition of sodium dodecyl sulphate (SDS) to a final concentration of 2% SDS and 50 mM triethylammonium bicarbonate (TEAB). Samples were digested with trypsin using the filter-aided sample preparation method [16]. Briefly, samples were reduced with 100 mM dithiothreitol at 60 °C for 30 min, transferred to 30 kDa MWCO Pall Nanosep centrifugation filters (Sigma-Aldrich), and washed several times with 8 M urea, and once with digestion buffer prior to alkylation with 10 mM methyl methanethiosulfonate in digestion buffer for 30 min. Digestion was performed by addition of trypsin (0.3 µg, Pierce MS-grade trypsin, Thermo Fisher Scientific) in 50 mM TEAB and 1% sodium deoxycholate (SDC) buffer at 37 °C overnight. An additional portion of enzyme was added and incubated for another 2 h. Peptides were collected by centrifugation and SDC was removed by acidification with 10% trifluoroacetic acid.

Samples were desalted using PepClean C18 spin columns (Thermo Fisher Scientific) according to the manufacturer’s guidelines prior to analysis on a Q Exactive mass spectrometer (Thermo Fisher Scientific) interfaced with an Easy nLC 1200 liquid chromatography system. Peptides were separated using an in-house constructed C18 analytical column (300 mm × 0.075 mm I.D., 3 μm, Dr. Maisch, Germany) using a gradient from 6 to 27% acetonitrile in 0.1% formic acid over 45 min followed by an increase to 80% acetonitrile in 0.1% formic acid for 5 min at a flow rate of 3 nL/min. Precursor ion mass spectra were acquired at 70 K resolution, and MS/MS analysis was performed in a data-dependent mode where the 10 most intense precursor ions were selected for fragmentation using HCD at a collision energy of 27. Charge states 2–6 were selected for fragmentation, and dynamic exclusion was set to 30 s.

Data analysis was performed using the Proteome Discoverer version 1.4 software (Thermo Fisher Scientific) and the Human SWISSPROT Database version Jan 2017 (Swiss Institute of Bioinformatics, Switzerland). Mascot 2.3 (Matrix Science) was used as the search engine with a precursor mass tolerance of 5 ppm and a fragment mass tolerance of 200 mmu. Tryptic peptides were accepted with one missed cleavage, and methionine oxidation and cysteine alkylation were set as variable modifications. The detected peptide threshold in the software was a 1% False Discovery Rate by searching against a reversed database, and identified proteins were grouped by shared sequences to minimise redundancy.

### Bioinformatics

The proteins identified with LC–MS/MS were analysed using the Database for Annotation, Visualization and Integrated Discovery (DAVID; http://david.abcc.ncifcrf.gov/) to identify cellular components associated with or enriched in the vesicle proteome. Venny (http://bioinfogp.cnb.csic.es/tools/venny/index.html) was used to compare lists of proteins to find common and unique molecules. Information from the exosome database EVpedia (http://www.evpedia.info/) [17] was accessed in April 2017.

## Results and discussion

### Size-exclusion chromatography fails to separate extracellular vesicles from lipoproteins

**Fig. 2:**
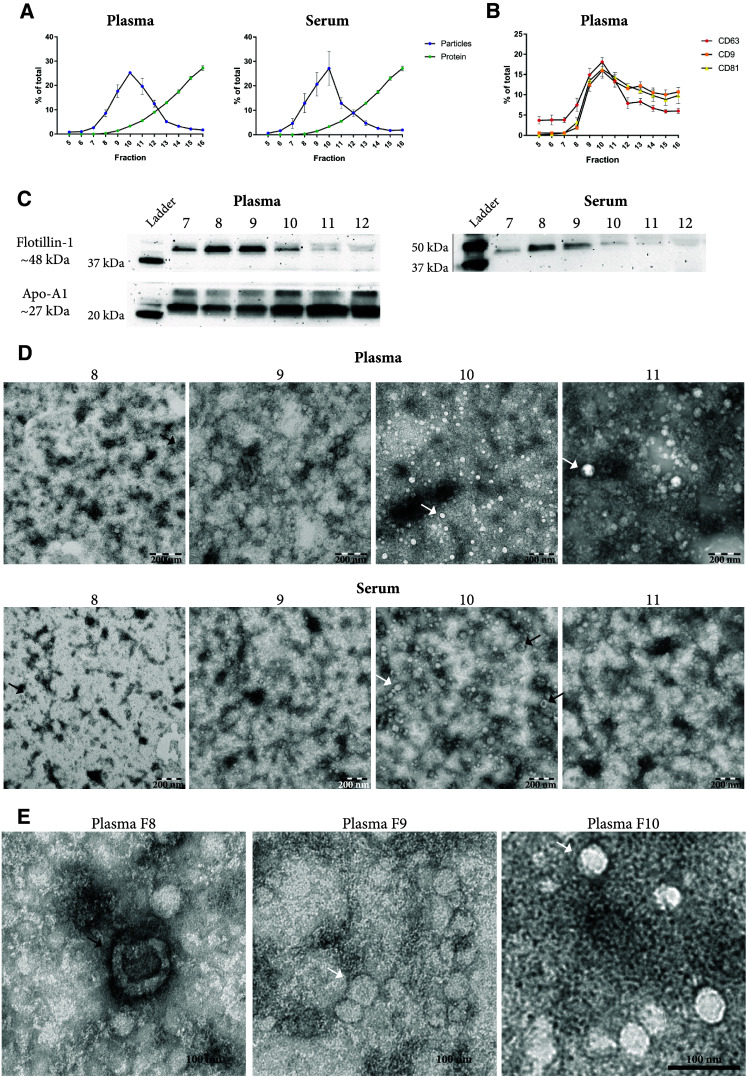
Evaluation of EVs isolated with size-exclusion chromatography (SEC). One millilitre of plasma or serum was loaded onto 10 mL Sepharose CL-2B columns, and up to 30 fractions of 500 µL were collected from each column. **a** Concentrations of particles and proteins in the SEC fractions were determined with nanoparticle tracking analysis (NTA; ZetaView^®^, blue) and BCA (green), respectively. Data are presented as the percentage of the total amount of particles or proteins in fractions 5–16. *N* = 4–6, and the results are presented as the average ± SEM. **b** ELISA was used to determine the expression of CD9, CD63, and CD81 on the vesicles in the SEC fractions. Data are presented as the percentage of the total expression for each protein  in fractions 5–16. *N* = 3–5, and the results are presented as the average ± SEM. **c** Presence of the vesicle marker flotillin-1 and the HDL marker Apo-A1 was determined in fractions 7–12 (40 µL/fraction) with Western blot. **d**, **e** Fifteen microliters (1–20 µg protein) from fractions 8–11 were loaded onto grids, negative stained, and evaluated with electron microscopy. Examples of EV-like structures (cup-shaped) are indicated by black arrows, and examples of lipoprotein particle-like structures (white structures) are indicated by white arrows. **e** Enlargements from fraction 8–10 from the plasma sample showed in **d**. Scale bars are 200 nm in **d** and 100 nm in **e**

One millilitre of plasma or serum was loaded onto an in-house-made Sepharose-based SEC column (Fig. 1) as previous described [15], and 28 fractions were collected. Nanoparticle tracking analysis (NTA) was used to determine the concentration of particles in each fraction. Because fractions 8–12 contained the highest concentrations of particles (Supplementary Figure 1), we limited the subsequent analysis to fractions 5–16. While fractions 8–12 contained the highest concentration of particles in both plasma and serum, peaking in fraction 10, the bulk of the proteins eluted in later fractions (Fig. 2a and Supplementary Figure 2), confirming the previous results [15].

We used an in-house developed ELISA system to determine the presence of the common EV markers CD9, CD63, and CD81 in the SEC fractions. All three markers were the most prominent in the fractions containing the highest concentrations of particles (Fig. 2b, fraction 8–12). Flotillin-1, also a marker of EVs, was most abundant in fractions 8 and 9 of both serum and plasma as determined by Western blot (Fig. 2c). However, Western blot also revealed that apolipoprotein A1 (Apo-A1), a marker for HDL and chylomicrons, was detectable in fractions 7–12, showing that lipoprotein particles were co-isolated with EVs (Fig. 2c). The presence of lipoprotein particles and plasma proteins was confirmed by electron microscopy (Fig. 2d, e). It is, however, important to note that when the degree of contamination is evaluated by measuring lipoproteins such as Apo-A, Apo-B, and Apo-E, proteins such as Apo-E have been reported to be present on exosomes, specifically those released from pigment cells [18], and EVs isolated from plasma can be covered with LDL [13], thus making it difficult to discriminate between lipoprotein particle contaminants and EV-associated lipoproteins.

We continued by analysing fractions 8 and 9 with mass spectrometry; however, only 88 proteins were identified (data not shown). Most of these proteins were plasma proteins, such as Apo-B, which is the primary apolipoprotein of chylomicrons (Apo B-48) and LDL and VLDL (Apo B-100). Other apolipoproteins known to be associated with chylomicrons and other lipoprotein particles were also observed. However, few EV-specific proteins were identified, with only 9 proteins identified from the top 100 human EV proteins listed on EVpedia [17]. Together, these results show that although fractions 8–12 contained particles with the size of EVs (Fig. 2a) and contained the highest concentrations of EV markers (Fig. 2b, c), electron microscopy and Western blot revealed that these fractions also contained lipoproteins and plasma proteins (Fig. 2c–e). Böing et al. used the same SEC column as we have used here, and they showed that the purest vesicles were found in fraction 9 [15]. When fractions 10, 11, and 12 were included, the recovery increased as these fractions also contained vesicles. However, as these fractions also contained more contaminants, this led to an overall decrease in purity. We observed a similar trend of decreased purity beyond fraction 10, but when we analysed fractions 8 and 9 with mass spectrometry, it was also clear that mainly lipoprotein and plasma proteins and relatively few vesicle proteins, could be identified.

### Sequential density gradient and size-exclusion chromatography separate EVs from lipoproteins

**Fig. 3:**
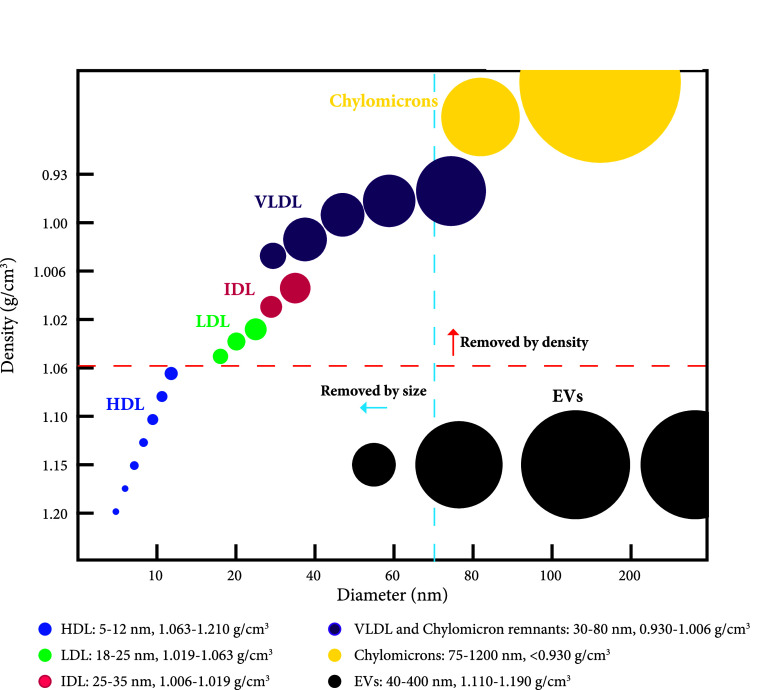
Schematic overview of the size and density of lipoproteins and EVs. Several of the lipoproteins such as high-density lipoproteins (HDL), low-density lipoproteins (LDL), intermediate-density lipoproteins (IDL), very low-density lipoproteins (VLDL), and chylomicrons overlap with extracellular vesicles (EVs) in terms of size or density. With an iodixanol density cushion of 10%/30%/50%, the 10% layer will create a density cutoff at approximate 1.06 g/cm^3^ (indicated by the orange dashed line). With Sepharose CL-2B SEC columns, the size cutoff is approximately 75 nm (indicated by the blue dashed line). The picture is modified from [11]

Because the concentration of chylomicrons increases in the blood after a meal, blood was collected from individuals after overnight fast. Despite this precaution, SEC alone was still unable to separate EVs from contaminating factors (Fig. 2c–e). Several lipoproteins have a diameter of less than 35 nm and would, therefore, be eluted in later fractions than EVs. On the other hand, chylomicrons and VLDL are overlapping in diameter with EVs, and co-isolation is expected during size-based separation by SEC. Because chylomicrons and VLDL differ in density from EVs, < 0.93, < 1.06, and > 1.10 g/cm^3^, respectively [11, 19], we decided to combine SEC with a density-based separation to further separate EVs from lipoprotein particles and chylomicrons. Density separation was thus expected to remove lipoprotein particles that are similar in size to EVs but differ in density, whereas SEC removes the lipoprotein particles with similar density to EVs but that differ in size (Fig. 3).

**Fig. 4:**
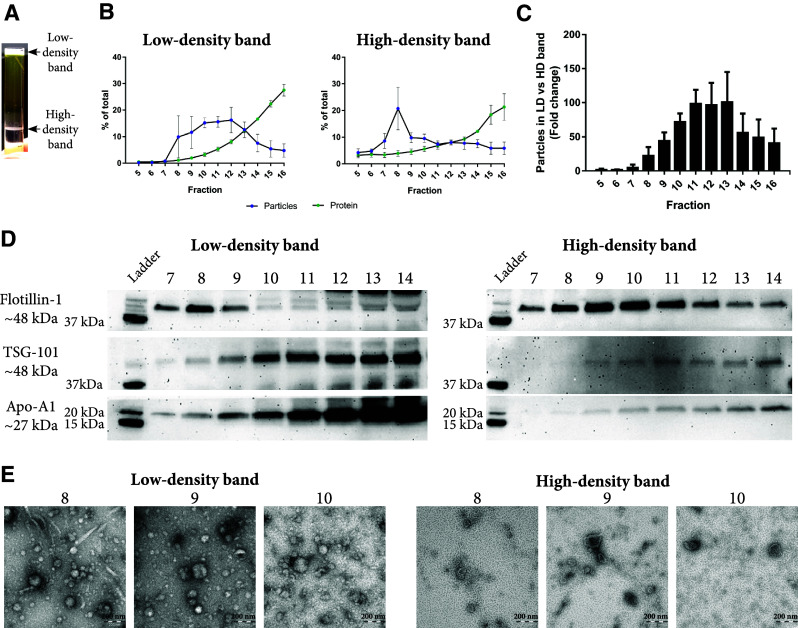
Evaluation of EVs isolated with the combination of density cushion and size-exclusion chromatography (IDC + SEC). Six millilitres of plasma were loaded on top of a density cushion (50%/30%/10% iodixanol), and visible bands after ultracentrifugation were further loaded onto 10 mL Sepharose CL-2B columns, and up to 30 fractions of 500 µL each were collected. **a** After centrifuging of the plasma sample on top of the cushion, two bands were visible. One band contained material floating above 1.025 g/cm^3^ (low-density band), and the second band contained material floating at approximately 1.06–1.16 g/cm^3^ (high-density band). **b** Low-density and high-density bands were loaded onto individual SEC columns, and fractions were collected. The concentrations of particles and proteins in the SEC fractions were determined by nanoparticle tracking analysis (NTA; ZetaView^®^, blue) and BCA (green), respectively. Data are presented as the percentage of the total amount of particles or proteins in fractions 5–16. *N* = 3, and the results are presented as the average ± SEM. **c** Total number of particles was determined by ZetaView^®^ in fractions 5–16 from the high-density and low-density bands isolated from the same plasma samples, and the fold change was calculated. *N* = 3, and the results are presented as the average ± SEM. *LD* low-density, *HD* high-density. **d** Presence of the vesicle markers flotillin-1 and TSG-101 as well as the HDL marker Apo-A1 was determined by Western blot of fractions 7–14 (40 µL/fraction) isolated from both the high-density and low-density band. **e** Fifteen microliters (1–6 µg protein) from fractions 8–10 from the high-density and low-density bands were loaded onto grids, negative stained, and evaluated with electron microscopy. Scale bars are 200 nm

Six millilitres of plasma were loaded on top of an iodixanol density cushion (Fig. 1) and centrifuged. The density cushion was carefully designed to allow the vesicles to float at approximately 1.06–1.16 g/cm^3^, while most lipoprotein particles have a lower density (Fig. 3) and, therefore, will float on top of the cushion. Following centrifugation, two bands were visible, one band containing material floating above the plasma at approximately < 1.025 g/cm^3^, here called the “low-density band”, which was enriched in lipoprotein particles, and a “high-density band” containing material floating at approximately 1.06–1.16 g/cm^3^, which was expected to be enriched in EVs (Fig. 4a). Next, the low-density and high-density bands were loaded onto individual SEC columns, and the concentrations of particles and proteins were measured in the eluted fractions. The majority of particles were recovered in fractions 7–14 for the low-density band, whereas the high-density band peaked at fraction 8 (Fig. 4b). When the absolute numbers of particles were compared between the same fractions of the low-density and high-density bands from the same sample, fractions 8–14 contained 30- to 100-fold more particles in the low-density band, suggesting that the chylomicrons and lipoprotein particles are substantially more abundant then EVs in plasma (Fig. 4c), supporting the previous observations [12–14]. This emphasises the difficulty that the EV field faces when working with highly complex samples such as plasma, where EVs are a tiny minority among other particles with similar physical features. This also highlights the limitations of NTA. Although NTA efficiently measures the particle abundance in SEC fractions, NTA fails to distinguish EVs from non-EV components. This becomes a problem especially when working with plasma and serum as lipoprotein particles overlap in size with EVs (Fig. 3). Thus, our results demonstrate that it is important to combine separation steps, each based on its own physical separation principle, when working with plasma, and to characterise the obtained isolates with several methods to determine the composition of the isolates.

Western blot showed that flotillin-1 was present in SEC fractions 7–9 of the low-density band and in fractions 7–14 of the high-density band (Fig. 4d). Interestingly, TSG-101 was mainly detected in SEC fractions 10–14 from both the low-density and high-density bands, suggesting the presence of a subpopulation of EVs positive for TSG-101 but negative for or containing lower amounts of flotillin-1 and eluting in later fractions than the flotillin-1-positive vesicles (Fig. 4d). It was surprising to us that the SEC fractions from the lipoprotein-enriched band (low-density band) also contained detectable levels of established vesicle markers. This indicates that particles or vesicles positive for classical EV markers are also present at densities < 1.025 g/cm^3^. This is puzzling as to our knowledge, there is little evidence suggesting that EVs can be found at this density. However, two studies have found EVs at these densities, one where prominin-1—containing particles were isolated from human epithelial colorectal adenocarcinoma cells at a density of 1.032–1.068 g/cm^3^ [20], and another study where vesicles were isolated from bone marrow-derived mesenchymal stem cells at a density of < 1.06 g/cm^3^ [21].

In this study, we used Sepharose CL-2B with a pore size of approximately 75 nm [22], which causes the EVs below this size to be eluted in later fractions together with the small lipoprotein particles and soluble plasma proteins. When evaluating the yield of vesicles in the different fractions, it is important to remember that NTA cannot detect smaller vesicles or particles that might be present in the analysed fractions nor in fractions 16, and later, because NTA has a lower limit of detection of about 70 nm for EVs, although this detection limit depends on the refractive index of the measured particles [23]. Thus, NTA cannot detect HDL and LDL particles that are eluted in the later fractions. Our finding that flotillin-1 and TSG-101 are also detectable in the fractions containing smaller particles stresses the need for a way to separate these potentially interesting EVs from small lipoproteins and plasma proteins, as well as the need for more sensitive instrumentation capable of detecting particles at such a minute size range.

Apo-A1 was mainly detected in the SEC fractions of the low-density band, suggesting that by implementing the density cushion before SEC, the lipoprotein particles can be efficiently separated from the plasma EVs (Fig. 4d). The higher particle concentration in the low-density band compared to the high-density band was further supported by electron microscopy (Fig. 4e). Furthermore, electron microscopy revealed that the low-density band contained more lipoprotein-like particles, while the high-density band contained more EV-like structures (Fig. 4e). Thus, the SEC fractions from the high-density band contained less contamination, and a purer EV isolate was generated when flotation and SEC were combined.

A commercial column, Exo-spin (CellGS), has previously been used to successfully isolate EVs from cell culture media from a prostate cancer cell line; however, when plasma was used, the fractions containing EVs overlapped with the fractions containing apolipoproteins [14]. Thus, this column also has a problem with separating EVs from lipoprotein particles, highlighting again that a combination of density cushion and SEC is probably essential when plasma or serum is used, although this problem might not be as great when cell culture media are used as a starting material, especially if the cells are cultured in a serum-free environment.

Due to the small number of vesicles in the high-density band, bands from two density cushions were combined and loaded onto a single SEC column, resulting in a total of 12 mL plasma as starting material, and fractions 8 and 9 were analysed with mass spectrometry. In total, 634 and 608 proteins were identified in fractions 8 and 9, respectively (data not shown), with approximately 90% overlap, indicating that vesicles in fractions 8 and 9 are similar in their protein cargo. In total, 86 proteins were identified from the top 100 human EV proteins listed at EVpedia [17]. It is, therefore, reasonable to conclude that the combination of flotation and density gradient is essential to remove contaminating lipid protein particles and plasma proteins for the analysis of plasma EV protein content. In addition, with this approach, the starting volume of plasma could be increased.

### The starting amount of plasma can be further increased with sequential ultracentrifugation, density gradient, and size-exclusion chromatography

**Fig. 5:**
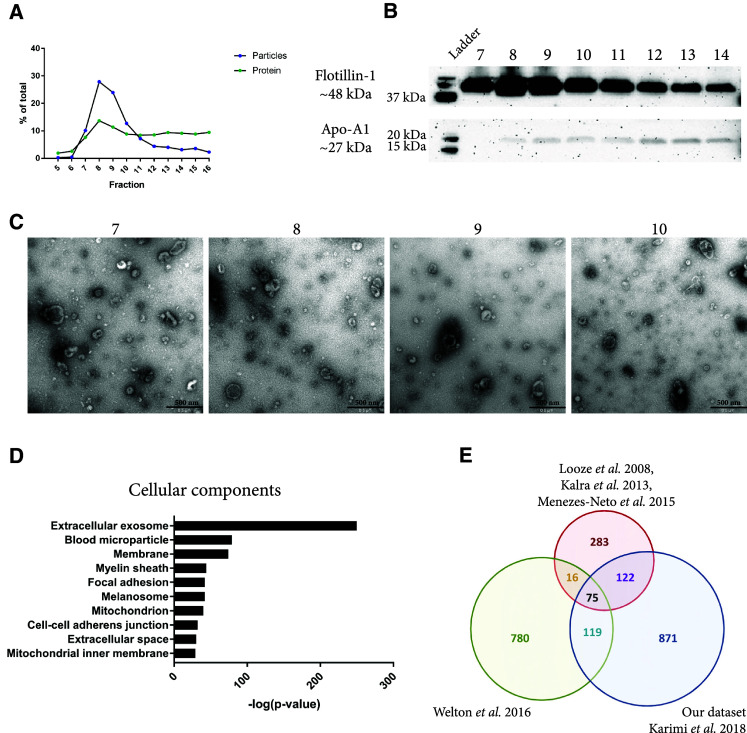
Evaluation of EVs isolated with the combination of ultracentrifugation, density cushion, and size-exclusion chromatography (UCF + IDC + SEC). To be able to increase the starting volume of plasma, two centrifugation steps were added. The pellets from 16,500×*g* and 118,000×*g* spins were re-suspended in PBS, mixed, loaded on top of a density cushion (50%/30%/10% iodixanol), and centrifuged. The band between 30 and 10% was subsequently loaded onto a 10 mL Sepharose CL-2B column, and up to 30 fractions of 500 µL each were collected. **a** Concentrations of particles and proteins in the SEC fractions were determined with nanoparticle tracking analysis (NTA; ZetaView^®^, blue) and BCA (green), respectively. Data are presented as the percentage of the total amount of particles or proteins in fractions 5–16. *N* = 1. **b** Presence of the vesicle marker flotillin-1 and the HDL marker Apo-A1 was determined with Western blot in fractions 7–14 (40 µL/fraction). **c** Five micrograms of protein (11–19 µL) from SEC fractions 7–10 were loaded onto grids, negative stained, and evaluated with electron microscopy. Scale bars are 500 nm. **d** LC–MS/MS was performed on the EVs isolated from fractions 8 and 9 and pooled. In total, 1187 proteins were identified and were analysed with DAVID Bioinformatics Resources 6.8 (https://david.ncifcrf.gov/). The ten most associated cellular compartments (based on *p* value) are listed in the graph. **e** The  1187 identified proteins were compared to previously published proteomes of plasma EVs [29–31, 33]

To increase the concentration of vesicles, ultracentrifugation was added to the procedure (Fig. 1), thereby offering the advantage that we could start with any volume of plasma. Plasma was centrifuged at 16,500×*g* and 120,000×*g*, and both pellets were dissolved in PBS, mixed, and loaded on top of a density cushion. The high-density band was subsequently loaded onto an SEC column and fractions were collected. In accordance with the previous observations, most particles eluted in fractions 8 and 9 (Fig. 5a). Western blot once more showed that the flotillin-1 signal was strongest in fractions 8 and 9; however, flotillin-1 was now also detectable in fractions 10–14 (Fig. 5b). Furthermore, Western blot showed low levels of Apo-A1 in fractions 7–14 (Fig. 5b). Electron microscopy confirmed that more vesicles were present in fractions 7–10 when the volume of starting material was increased (Fig. 5c compared to Fig. 4e). Fractions 8 and 9 were combined and analysed with mass spectrometry, and in total, 1187 proteins were identified (Supplementary Table 1). A total of 85 proteins were identified from the top 100 human EV proteins listed on EVpedia [17] as well as several common EV proteins such as Rab proteins, annexins, tetraspanins, heat shock proteins, and ESCRT proteins (Table 1). The identified proteins were analysed with GO Term Finder to identify enriched cellular components compared to the genome frequency, and the top associated terms were “Extracellular exosome”, “Blood microparticle”, and “Membrane” (Fig. 5d), supporting the conclusion that EVs of high purity had, indeed, been isolated.

**Table 1:** Identification of common EV proteins

Protein group	Proteins
Rabs	**Rab-1A, -1B**, **-2A**, **-2B**, **-4A**, **-4B**, **-5A**, **-5B**, **-5C**, **-6A**, **-6B**, **-7a**, **-8A**, **-8B** **Rab-10**, **-11B**, **-14**, **-18** **Rab-21**, **-27B** **Rab-30**, **-32**, **-33A**, **-35**, **-37**, **-38**
Annexins	Annexin A2, **Annexin A4**, **Annexin A7**, **Annexin A11**
Tetraspanins	**CD9**, **CD63**, **CD81**, **CD82**, **CD151**, **TSPAN2**, **TSPAN14**, **TSPAN32**
Common EV markers	**MHC class I**, **Ezrin**, Flotillin-1, Flotillin-2, **Cofilin-1**, **Profilin-1**, **CD59**, 14-3-3 protein (beta/alpha, epsilon, eta, gamma, sigma, theta, zeta/delta)
Heat shock proteins	Heat shock 70 kDa protein 1A/1B Heat shock cognate 71 kDa **Heat shock protein 75** **kDa, mitochondrial** Heat shock protein beta-1 Heat shock protein HSP 90-alpha and beta
ESCRT	ESCRT-0—ND ESCRT-I—**VPS-28**, **VPS-37B** ESCRT-II—ND ESCRT-III—**CHMP4B**, **CHMP6** ESCRT accessory—**Clathrin**, **Alix**

Proteins highlighted in bold are the proteins that have not been previously identified in plasma EVs with mass spectrometry or SOMAscan^®^ [29–31, 33]

*ND* not detected

### Protein analysis of plasma-derived extracellular vesicles

In an attempt to determine the cellular origin of the isolated vesicles, the presence of markers for several cells was evaluated in the proteomic data set. In the plasma EVs, the following cell markers were detected with mass spectrometry: CD235a (glycophorin-A) for erythrocytes; CD41, CD61, and CD62p for platelets; and CD56 for NK cells. However, neurons, glia cells, and skeletal muscle cells can also express CD56, which is why it cannot be concluded that theses EVs really originate from NK cells. The expression of the following specific cell type proteins was not detectable in plasma EVs: CD326 (EpCAM) for epithelial cells, CD146 for endothelial cells, CD45 for leukocytes, CD66b for granulocytes, CD14 and CD33 for macrophages/monocytes, CD34 for hematopoietic stem cells, CD11c and CD123 for dendritic cells, CD19 and CD20 for B cells, and CD3, CD4, and CD8 for T cells. Importantly, we did detect MHC class I but not MHC class II, the latter being expressed only on antigen presenting cells. Together, these data show that the majority of the plasma EVs originated from erythrocytes and platelets; however, even though we were unable to detect other cell-specific markers in our present analysis, these other cells might still contribute to the mixture of circulating EVs.

Interestingly, the membrane proteins CD55, CD59, and CD47 were identified in plasma EVs. CD55 and CD59 protect cells against lysis by the complement complex [24], and it has been demonstrated that when expressed on exosomes, these molecules protect against complement-mediated vesicle lysis [25]. The expression of CD47, which is extensively expressed on red blood cells, is considered to prevent recognition by macrophages, and it is referred to as a “don’t-eat-me” signal or “marker of self” [26]. The presence of these three proteins on the plasma-derived EVs suggests that they are at least partly protected from rapid consumption in the circulation thus allowing for prolonged circulation time.

The concentration of albumin in plasma ranges between 30 and 50 mg/mL [27], and albumin is, therefore, the main contaminant when plasma EVs are isolated by ultracentrifugation, irrespectively of washings [28]. Therefore, several previous studies determining the proteome of plasma EVs by mass spectrometry have used other isolation methods such as SEC, commercial columns, density gradients, and immune-affinity capture [29, 30]. Looze et al. were the first to use mass spectrometry on EVs isolated from human plasma [31]. Although an ambitious approach including both gel exclusion chromatography and rate zonal centrifugation was applied to isolate vesicles, only 66 proteins could be identified [31]. Kalra et al. identified 213 proteins using three different isolation methods for plasma EVs [29]. In addition, de Menezes-Neto et al. used different isolation methods and identified 330 proteins [30]. In total, these studies together identified approximately 400 proteins in EVs isolated from human plasma [29–32]. However, only half of the identified proteins were observed in more than one study, demonstrating a large heterogeneity between studies [30]. Furthermore, relatively few specific vesicle markers were identified, demonstrating how difficult it is to isolate and separate EVs from abundant plasma proteins [30]. In addition, Welton et al. used SEC to isolate plasma EVs, but in this study, only 21 proteins could be detected by mass spectrometry, and these proteins were mainly soluble plasma proteins, despite removal of more than > 97% of the proteins [33]. Thus, the authors used a protein array platform, SOMAscan^®^, instead of mass spectrometry and could then detect approximately 1000 proteins [33]. Because SOMAscan^®^ is a multiplex aptamer-based protein array, only aptamer-binding proteins were detected. Therefore, several exosomal markers such as CD9, CD81, and ezrin were not found, as they were not among the 1300 proteins that the aptamer-based assay had been designed for. Thus, although the authors increased the identified proteins from 21 to over a 1000 using a multiplex protein assay, this technique still has limitations.

When we compare our data set to these previously published data sets, it is clear that the overlap is very small and that we have identified almost 800 novel proteins previously not identified in plasma EVs (Fig. 5e). Several of these proteins have been identified in previous proteomic studies on EVs from other sources such as cell lines (Table 1, highlighted in bold) and some of these EV proteins, such as CD63, CD9, and CD81, have previously been identified in plasma EVs with Western blot, electron microscopy, or flow cytometry [15, 29, 30, 33, 34], but so far never with mass spectrometry. Thus, our current study has identified many novel plasma EV proteins, far beyond what has previously been reported. This highlights two important conclusions from our study. First, a combination of several purification steps is required to generate a sample that is pure enough for mass spectrometry. Second, several particles observed in plasma are, indeed, not vesicles, and it is, therefore, most likely not yet possible to isolate sufficient EVs from a small volume of plasma (< 10 mL) for subsequent analyses using the current state-of-the-art techniques.

### RNA analysis of plasma-derived extracellular vesicles

**Fig. 6:**
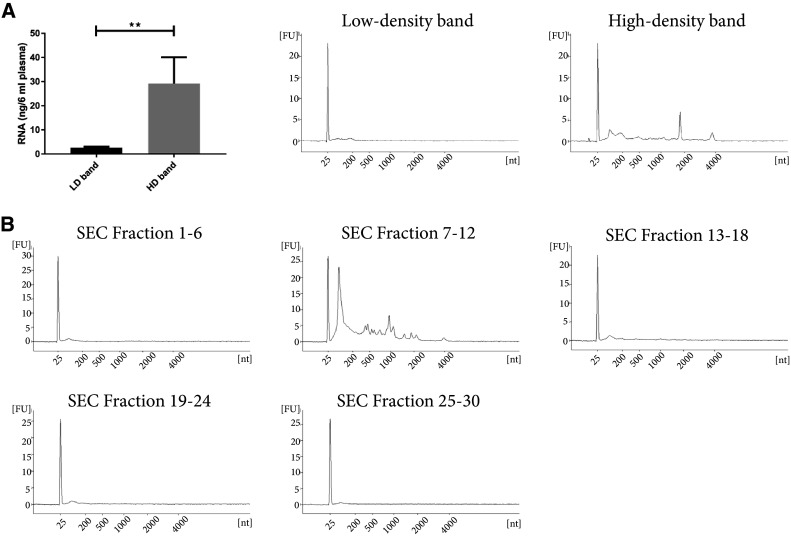
RNA isolation from EV-enriched and lipoprotein-enriched fractions. **a** Six millilitres of plasma were loaded onto a 50%/30%/10% iodixanol density cushion, and the low-density and high-density bands were isolated. RNA was isolated with a miRCURY RNA Isolation Kit—Cell and Plant (Exiqon) directly from 300 µL of the high-density and low-density bands and analysed with a Bioanalyzer^®^ (Agilent). *N* = 4–6, and the results are presented as the average ± SEM. **p value < 0.01. *LD* low-density, *HD* high-density. **b** Fifty-eight millilitres of plasma were ultracentrifuged, and the pellets from a 16,500×*g* and 118,000×*g* spin were re-suspended in PBS, mixed, loaded on top of a density cushion (50%/30%/10% iodixanol) and centrifuged. The band between 30 and 10% was subsequently loaded onto a 10 mL Sepharose CL-2B column and 30 fractions of 500 µL each were collected in pools of 6 fractions (3 mL in total/pool). The sample pools were ultracentrifuged, and RNA was isolated and analysed as in **a**

Both EVs [7, 35] and HDL [36] have been shown to contain RNA. Interestingly, it has been shown that EVs of different densities differ in RNA content [35, 37]. Thus, the amount of and characteristics of the RNA cargo could be partly responsible for the different densities, with EVs of higher density containing more RNA [35]. Based on the densities for different biomolecules (protein (1.35 g/cm^3^), RNA and DNA (1.7 g/cm^3^), and (lipids ~ 1 g/cm^3^) [38, 39]), the particles/vesicles floating at a density as low as < 1.025 g/cm^3^ should be composed of > 90% lipids and have very low concentrations of both RNA and protein. We, therefore, analysed the RNA content in the high-density and low-density bands to determine whether the lipoprotein particles with lower density also contain RNA. Bioanalyzer analysis showed that the EV-enriched band (high-density band) contained significantly more RNA than the lipoprotein-enriched band (low-density band) (Fig. 6a). The high-density band was then separated further by SEC, and the collected fractions were pooled (fractions 1–6, 7–12, 13–18, 19–24, and 25–30) prior to ultracentrifugation and RNA analysis. The RNA in the high-density band was mainly present in SEC fractions 7–12 (Fig. 6b), which again were the fractions containing most of the EVs and no lipoprotein particles (Figs. 4d, e, 5a–d). These data strongly suggest that most of the circulating RNA is, indeed, packed in the EVs and that lipoprotein particles, except HDL, and a potential subpopulation of low-density EVs contain little or no RNA.

## Conclusion

Isolation of EVs from bio-fluids for downstream analysis is still problematic, because many body fluids have a complex biochemical and physical composition. This is particularly true for plasma and serum, making it difficult to obtain pure EV isolates from such fluids. This study demonstrates that a two-step isolation procedure, combining density cushion separation followed by SEC, isolates EVs from human plasma, and efficiently separates EVs from the main contaminants lipoproteins and plasma proteins. By floating the vesicles on a density cushion, the EVs could be separated from the chylomicrons and other lipoproteins with lower densities than EVs, and by loading the floated EVs on an SEC column, the EVs could be further separated from soluble proteins and lipoproteins with a smaller size than EVs. With this isolation approach, plasma EVs of previously unattained purity could be identified by electron microscopy and Western blot, and in total, 1187 proteins could be identified in the EV isolates with mass spectrometry, without excessive contamination of plasma proteins and lipoprotein particles. Several of these proteins have been identified previously in EVs isolated from different sources, but they have not previously been detected with mass spectrometry using plasma EVs isolated by other methods. Again, the results presented here support the feasibility of the combined use of flotation and SEC to isolate highly pure EVs from blood. However, the need for these highly purified blood-derived EVs might depend on the downstream analyses. As the lipoprotein particles with low density only contained low or no amounts of RNA, the requirement of purification might be less if it is the RNA that will be analysed. Furthermore, if a focused analysis such as ELISA or the SOMAscan^®^ assay is used, the problem of contaminating protein is probably smaller; however, for mass spectrometry analysis, for example, this approach is crucial as well as for functional studies.

In conclusion, by combining a density cushion and SEC, we could for the first time isolate EVs from plasma with high purity. Furthermore, we show that the majority of particles detected in plasma are not EVs, which emphasises the relevance of stringent isolation methods prior to the analysis of EV cargo and function to avoid studying non-EV-associated features.

### Electronic supplementary material

Below is the link to the electronic supplementary material.

Supplementary Figure 1. Nanoparticle tracking analysis of 28 fractions isolated from plasma with size-exclusion chromatography. The concentration of particles in each SEC fraction was determined with nanoparticle tracking analysis (NTA; ZetaView^®^) (PDF 117 kb)

Supplementary Figure 2. PageBlue staining of 27 fractions isolated from plasma with size-exclusion chromatography. The presence of proteins in each SEC fraction from plasma was determined by SDS-PAGE of 40 µL samples from each 500 µL fraction on precast 4-20% gradient gels (PDF 75 kb)

Supplementary material 3. The list of the 1187 proteins identified in healthy plasma-derived extracellularvesicles (PDF 222 kb)

## References

[1] Anderson NL, Anderson NG (2002). The human plasma proteome: history, character, and diagnostic prospects. Mol Cell Proteom.

[2] Perakis S, Speicher MR (2017). Emerging concepts in liquid biopsies. BMC Med.

[3] Lässer C (2015). Exosomes in diagnostic and therapeutic applications: biomarker, vaccine and RNA interference delivery vehicle. Expert Opin Biol Ther.

[4] Yanez-Mo M, Siljander PR, Andreu Z (2015). Biological properties of extracellular vesicles and their physiological functions. J Extracell Vesicles.

[5] Shelke GV, Jang SC, Yin Y (2016). Human mast cells release extracellular vesicle-associated DNA. Matters.

[6] Lazaro-Ibanez E, Sanz-Garcia A, Visakorpi T (2014). Different gDNA content in the subpopulations of prostate cancer extracellular vesicles: apoptotic bodies, microvesicles, and exosomes. Prostate.

[7] Valadi H, Ekström K, Bossios A (2007). Exosome-mediated transfer of mRNAs and microRNAs is a novel mechanism of genetic exchange between cells. Nat Cell Biol.

[8] Skog J, Wurdinger T, van Rijn S (2008). Glioblastoma microvesicles transport RNA and proteins that promote tumour growth and provide diagnostic biomarkers. Nat Cell Biol.

[9] Logozzi M, De Milito A, Lugini L (2009). High levels of exosomes expressing CD63 and caveolin-1 in plasma of melanoma patients. PLoS One.

[10] Eldh M, Olofsson Bagge R, Lasser C (2014). MicroRNA in exosomes isolated directly from the liver circulation in patients with metastatic uveal melanoma. BMC Cancer.

[11] Feingold KR, Grunfeld C (2015) Introduction to lipids and lipoproteins. In: De Groot LJ, Chrousos G, Dungan K, Feingold KR, Grossman A, Hershman JM, Koch C, Korbonits M, McLachlan R, New M et al (eds) Endotext [Internet]. MDText.com, Inc, South Dartmouth (MA)

[12] Yuana Y, Levels J, Grootemaat A (2014). Co-isolation of extracellular vesicles and high-density lipoproteins using density gradient ultracentrifugation. J Extracell Vesicles.

[13] Sodar BW, Kittel A, Paloczi K (2016). Low-density lipoprotein mimics blood plasma-derived exosomes and microvesicles during isolation and detection. Sci Rep.

[14] Welton JL, Webber JP, Botos LA (2015). Ready-made chromatography columns for extracellular vesicle isolation from plasma. J Extracell Vesicles.

[15] Boing AN, van der Pol E, Grootemaat AE (2014). Single-step isolation of extracellular vesicles by size-exclusion chromatography. J Extracell Vesicles.

[16] Gross JC, Chaudhary V, Bartscherer K (2012). Active Wnt proteins are secreted on exosomes. Nat Cell Biol.

[17] Kim DK, Lee J, Kim SR (2015). EVpedia: a community web portal for extracellular vesicles research. Bioinformatics.

[18] van Niel G, Bergam P, Di Cicco A (2015). Apolipoprotein E regulates amyloid formation within endosomes of pigment cells. Cell Rep.

[19] Thery C, Ostrowski M, Segura E (2009). Membrane vesicles as conveyors of immune responses. Nat Rev Immunol.

[20] Marzesco AM, Janich P, Wilsch-Brauninger M (2005). Release of extracellular membrane particles carrying the stem cell marker prominin-1 (CD133) from neural progenitors and other epithelial cells. J Cell Sci.

[21] Didiot MC, Hall LM, Coles AH (2016). Exosome-mediated delivery of hydrophobically modified siRNA for huntingtin mRNA silencing. Mol Ther J Am Soc Gene Ther.

[22] Hagel L, Östberg M, Andersson T (1996). Apparent pore size distributions of chromatography media. J Chromatogr A.

[23] van der Pol E, Coumans FA, Grootemaat AE (2014). Particle size distribution of exosomes and microvesicles determined by transmission electron microscopy, flow cytometry, nanoparticle tracking analysis, and resistive pulse sensing. J Thromb Haemost.

[24] Raposo G, Stoorvogel W (2013). Extracellular vesicles: exosomes, microvesicles, and friends. J Cell Biol.

[25] Colombo M, Raposo G, Thery C (2014). Biogenesis, secretion, and intercellular interactions of exosomes and other extracellular vesicles. Annu Rev Cell Dev Biol.

[26] Kalra H, Drummen GP, Mathivanan S (2016). Focus on extracellular vesicles: introducing the next small big thing. Int J Mol Sci.

[27] Executive Committee of the German Medical Association on the Recommendation of the Scientific Advisory Board (2016) Cross-sectional guidelines for therapy with blood components and plasma derivatives, Chap 5 Human Albumin—Revised. Transfus Med Hemother 43(3):223–232 10.1159/000446043 PMC4924448 27403094

[28] Baranyai T, Herczeg K, Onodi Z (2015). Isolation of exosomes from blood plasma: qualitative and quantitative comparison of ultracentrifugation and size exclusion chromatography methods. PLoS One.

[29] Kalra H, Adda CG, Liem M (2013). Comparative proteomics evaluation of plasma exosome isolation techniques and assessment of the stability of exosomes in normal human blood plasma. Proteomics.

[30] de Menezes-Neto A, Saez MJ, Lozano-Ramos I (2015). Size-exclusion chromatography as a stand-alone methodology identifies novel markers in mass spectrometry analyses of plasma-derived vesicles from healthy individuals. J Extracell Vesicles.

[31] Looze C, Yui D, Leung L (2009). Proteomic profiling of human plasma exosomes identifies PPARgamma as an exosome-associated protein. Biochem Biophys Res Commun.

[32] Bastos-Amador P, Royo F, Gonzalez E (2012). Proteomic analysis of microvesicles from plasma of healthy donors reveals high individual variability. J Proteom.

[33] Welton JL, Brennan P, Gurney M (2016). Proteomics analysis of vesicles isolated from plasma and urine of prostate cancer patients using a multiplex, aptamer-based protein array. J Extracell Vesicles.

[34] Lässer C, Alikhani VS, Ekström K (2011). Human saliva, plasma and breast milk exosomes contain RNA: uptake by macrophages. J Transl Med.

[35] Lässer C, Shelke GV, Yeri A (2017). Two distinct extracellular RNA signatures released by a single cell type identified by microarray and next-generation sequencing. RNA Biol.

[36] Vickers KC, Palmisano BT, Shoucri BM (2011). MicroRNAs are transported in plasma and delivered to recipient cells by high-density lipoproteins. Nat Cell Biol.

[37] Willms E, Johansson HJ, Mager I (2016). Cells release subpopulations of exosomes with distinct molecular and biological properties. Sci Rep.

[38] Riva S, Barrai I, Cavalli-Sforza L (1969). Dependence of the buoyant density of single-stranded DNA on base composition. J Mol Biol.

[39] Fischer H, Polikarpov I, Craievich AF (2004). Average protein density is a molecular-weight-dependent function. Protein Sci Publ Protein Soc.

[40] O’Driscoll L, Stoorvogel W, Thery C (2017). European network on microvesicles and exosomes in health and disease (ME-HaD). Eur J Pharm Sci Off J Eur Fed Pharm Sci.

