# Supplementary figures and images for: Detailed analysis of the plasma extracellular vesicle proteome after separation from lipoproteins
Source: Cell Mol Life Sci. 2018 Feb 13;75(15):2873–86. doi: 10.1007/s00018-018-2773-4 (PMC6021463; doi:10.1007/s00018-018-2773-4)

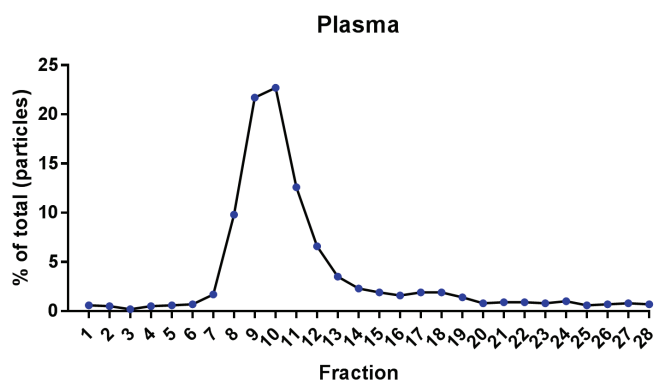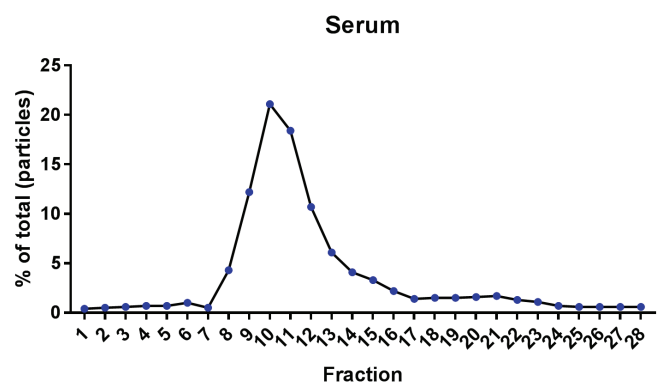

**Supplementary Figure 1**

Supplement: Supplementary file 1 — Supplementary Figure 1. Nanoparticle tracking analysis of 28 fractions isolated from plasma with size-exclusion chromatography. The concentration of particles in each SEC fraction was determined with nanoparticle tracking analysis (NTA; ZetaView®) (PDF 117 kb) [file 18_2018_2773_MOESM1_ESM.pdf]

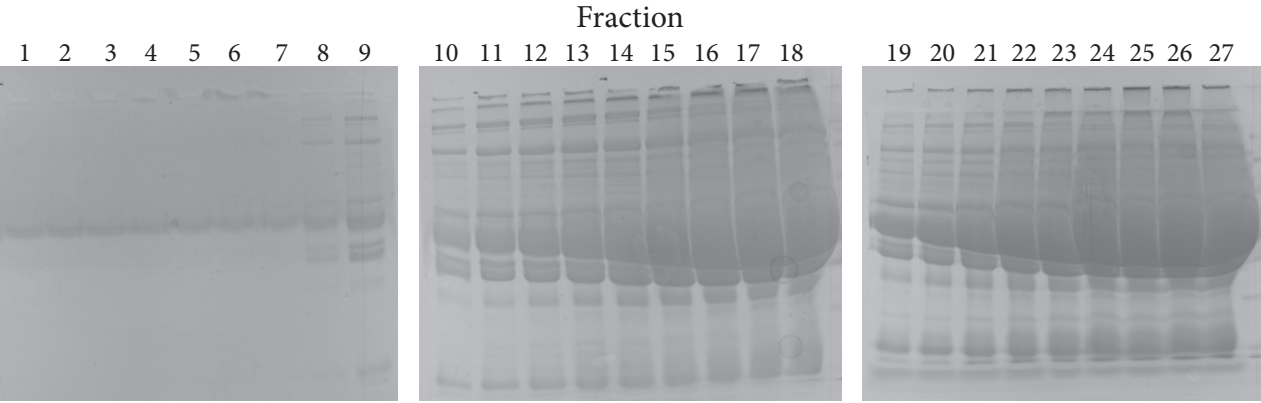

**Supplementary Figure 2**

Supplement: Supplementary file 2 — Supplementary Figure 2. PageBlue staining of 27 fractions isolated from plasma with size-exclusion chromatography. The presence of proteins in each SEC fraction from plasma was determined by SDS-PAGE of 40 µL samples from each 500 µL fraction on precast 4-20% gradient gels (PDF 75 kb) [file 18_2018_2773_MOESM2_ESM.pdf]
