# Supplementary material for: Detailed analysis of the plasma extracellular vesicle proteome after separation from lipoproteins
Source: Cell Mol Life Sci. 2018 Feb 13;75(15):2873–86. doi: 10.1007/s00018-018-2773-4 (PMC6021463; doi:10.1007/s00018-018-2773-4)
Supplement: Supplementary file 3 — Supplementary material 3. The list of the 1187 proteins identified in healthy plasma-derived extracellularvesicles (PDF 222 kb) [file 18_2018_2773_MOESM3_ESM.pdf]

Supplementery Table 1

Accession	Description	ΣCoverage	# Peptides
Q9P0J1	[Pyruvate dehydrogenase [acetyl-transferring]]-phosphatase 1, mitochondrial OS=Homo sapiens GN=PDP1 PE=1 SV=3 - [PDP1_HU	2,98	1
P61604	10 kDa heat shock protein, mitochondrial OS=Homo sapiens GN=HSPE1 PE=1 SV=2 - [CH10_HUMAN]	41,18	3
P31946	14-3-3 protein beta/alpha OS=Homo sapiens GN=YWHAB PE=1 SV=3 - [1433B_HUMAN]	43,90	9
P62258	14-3-3 protein epsilon OS=Homo sapiens GN=YWHAE PE=1 SV=1 - [1433E_HUMAN]	47,06	11
Q04917	14-3-3 protein eta OS=Homo sapiens GN=YWHAH PE=1 SV=4 - [1433F_HUMAN]	45,93	10
P61981	14-3-3 protein gamma OS=Homo sapiens GN=YWHAG PE=1 SV=2 - [1433G_HUMAN]	42,11	7
P31947	14-3-3 protein sigma OS=Homo sapiens GN=SFN PE=1 SV=1 - [1433S_HUMAN]	21,77	4
P27348	14-3-3 protein theta OS=Homo sapiens GN=YWHAQ PE=1 SV=1 - [1433T_HUMAN]	42,45	7
P63104	14-3-3 protein zeta/delta OS=Homo sapiens GN=YWHAZ PE=1 SV=1 - [1433Z_HUMAN]	59,59	11
Q99943	1-acyl-sn-glycerol-3-phosphate acyltransferase alpha OS=Homo sapiens GN=AGPAT1 PE=2 SV=2 - [PLCA_HUMAN]	7,42	1
Q9NUQ2	1-acyl-sn-glycerol-3-phosphate acyltransferase epsilon OS=Homo sapiens GN=AGPAT5 PE=1 SV=3 - [PLCE_HUMAN]	3,30	1
Q9NRZ7	1-acyl-sn-glycerol-3-phosphate acyltransferase gamma OS=Homo sapiens GN=AGPAT3 PE=1 SV=1 - [PLCC_HUMAN]	2,66	1
Q01970	1-phosphatidylinositol 4,5-bisphosphate phosphodiesterase beta-3 OS=Homo sapiens GN=PLCB3 PE=1 SV=2 - [PLCB3_HUMAN]	0,81	1
Q16698	2,4-dienoyl-CoA reductase, mitochondrial OS=Homo sapiens GN=DECR1 PE=1 SV=1 - [DECR_HUMAN]	12,54	4
P62195	26S protease regulatory subunit 8 OS=Homo sapiens GN=PSMC5 PE=1 SV=1 - [PRS8_HUMAN]	3,20	1
Q13200	26S proteasome non-ATPase regulatory subunit 2 OS=Homo sapiens GN=PSMD2 PE=1 SV=3 - [PSMD2_HUMAN]	2,09	1
Q02218	2-oxoglutarate dehydrogenase, mitochondrial OS=Homo sapiens GN=OGDH PE=1 SV=3 - [ODO1_HUMAN]	4,69	3
P52815	39S ribosomal protein L12, mitochondrial OS=Homo sapiens GN=MRPL12 PE=1 SV=2 - [RM12_HUMAN]	6,57	1
Q13405	39S ribosomal protein L49, mitochondrial OS=Homo sapiens GN=MRPL49 PE=1 SV=1 - [RM49_HUMAN]	8,43	1
Q99714	3-hydroxyacyl-CoA dehydrogenase type-2 OS=Homo sapiens GN=HSD17B10 PE=1 SV=3 - [HCD2_HUMAN]	22,99	4
P31937	3-hydroxyisobutyrate dehydrogenase, mitochondrial OS=Homo sapiens GN=HIBADH PE=1 SV=2 - [3HIDH_HUMAN]	3,87	1
P09110	3-ketoacyl-CoA thiolase, peroxisomal OS=Homo sapiens GN=ACAA1 PE=1 SV=2 - [THIK_HUMAN]	2,12	1
P08195	4F2 cell-surface antigen heavy chain OS=Homo sapiens GN=SLC3A2 PE=1 SV=3 - [4F2_HUMAN]	9,68	1
P49189	4-trimethylaminobutyraldehyde dehydrogenase OS=Homo sapiens GN=ALDH9A1 PE=1 SV=3 - [AL9A1_HUMAN]	4,25	2
Q00013	55 kDa erythrocyte membrane protein OS=Homo sapiens GN=MPP1 PE=1 SV=2 - [EM55_HUMAN]	4,72	1
P10809	60 kDa heat shock protein, mitochondrial OS=Homo sapiens GN=HSPD1 PE=1 SV=2 - [CH60_HUMAN]	22,16	11
P17858	6-phosphofructokinase, liver type OS=Homo sapiens GN=PFKL PE=1 SV=6 - [K6PL_HUMAN]	3,59	1
P11021	78 kDa glucose-regulated protein OS=Homo sapiens GN=HSPA5 PE=1 SV=2 - [GRP78_HUMAN]	41,59	17
Q9UBM7	7-dehydrocholesterol reductase OS=Homo sapiens GN=DHCR7 PE=1 SV=1 - [DHCR7_HUMAN]	1,89	1
O95870	Abhydrolase domain-containing protein 16A OS=Homo sapiens GN=ABHD16A PE=1 SV=3 - [ABHGA_HUMAN]	17,56	4
Q8IZP0	Abl interactor 1 OS=Homo sapiens GN=ABI1 PE=1 SV=4 - [ABI1_HUMAN]	2,17	1
A1L0T0	Acetolactate synthase-like protein OS=Homo sapiens GN=ILVBL PE=1 SV=2 - [ILVBL_HUMAN]	3,80	1
Q9NUB1	Acetyl-coenzyme A synthetase 2-like, mitochondrial OS=Homo sapiens GN=ACSS1 PE=1 SV=2 - [ACS2L_HUMAN]	2,03	1
Q13510	Acid ceramidase OS=Homo sapiens GN=ASAH1 PE=1 SV=5 - [ASAH1_HUMAN]	7,85	1
Q99798	Aconitate hydratase, mitochondrial OS=Homo sapiens GN=ACO2 PE=1 SV=2 - [ACON_HUMAN]	9,10	6
P60709	Actin, cytoplasmic 1 OS=Homo sapiens GN=ACTB PE=1 SV=1 - [ACTB_HUMAN]	45,87	12
P63267	Actin, gamma-enteric smooth muscle OS=Homo sapiens GN=ACTG2 PE=1 SV=1 - [ACTH_HUMAN]	25,27	8
P61160	Actin-related protein 2 OS=Homo sapiens GN=ACTR2 PE=1 SV=1 - [ARP2_HUMAN]	9,90	1

O15143	Actin-related protein 2/3 complex subunit 1B OS=Homo sapiens GN=ARPC1B PE=1 SV=3 - [ARC1B_HUMAN]	9,14	1
O15144	Actin-related protein 2/3 complex subunit 2 OS=Homo sapiens GN=ARPC2 PE=1 SV=1 - [ARPC2_HUMAN]	30,00	1
P59998	Actin-related protein 2/3 complex subunit 4 OS=Homo sapiens GN=ARPC4 PE=1 SV=3 - [ARPC4_HUMAN]	17,86	2
O15511	Actin-related protein 2/3 complex subunit 5 OS=Homo sapiens GN=ARPC5 PE=1 SV=3 - [ARPC5_HUMAN]	16,56	2
P61158	Actin-related protein 3 OS=Homo sapiens GN=ACTR3 PE=1 SV=3 - [ARP3_HUMAN]	16,99	3
Q9NPJ3	Acyl-coenzyme A thioesterase 13 OS=Homo sapiens GN=ACOT13 PE=1 SV=1 - [ACO13_HUMAN]	15,71	2
O14734	Acyl-coenzyme A thioesterase 8 OS=Homo sapiens GN=ACOT8 PE=1 SV=1 - [ACOT8_HUMAN]	2,51	1
Q9Y305	Acyl-coenzyme A thioesterase 9, mitochondrial OS=Homo sapiens GN=ACOT9 PE=1 SV=2 - [ACOT9_HUMAN]	5,24	2
O75608	Acyl-protein thioesterase 1 OS=Homo sapiens GN=LYPLA1 PE=1 SV=1 - [LYPA1_HUMAN]	4,78	1
Q8TB61	Adenosine 3'-phospho 5'-phosphosulfate transporter 1 OS=Homo sapiens GN=SLC35B2 PE=1 SV=1 - [S35B2_HUMAN]	2,78	1
O43306	Adenylate cyclase type 6 OS=Homo sapiens GN=ADCY6 PE=1 SV=2 - [ADCY6_HUMAN]	8,22	5
P54819	Adenylate kinase 2, mitochondrial OS=Homo sapiens GN=AK2 PE=1 SV=2 - [KAD2_HUMAN]	4,60	1
P00568	Adenylate kinase isoenzyme 1 OS=Homo sapiens GN=AK1 PE=1 SV=3 - [KAD1_HUMAN]	5,15	1
Q01518	Adenylyl cyclase-associated protein 1 OS=Homo sapiens GN=CAP1 PE=1 SV=5 - [CAP1_HUMAN]	25,89	7
Q9HDC9	Adipocyte plasma membrane-associated protein OS=Homo sapiens GN=APMAP PE=1 SV=2 - [APMAP_HUMAN]	21,39	4
Q15848	Adiponectin OS=Homo sapiens GN=ADIPOQ PE=1 SV=1 - [ADIPO_HUMAN]	12,30	1
P05141	ADP/ATP translocase 2 OS=Homo sapiens GN=SLC25A5 PE=1 SV=7 - [ADT2_HUMAN]	19,13	4
P12236	ADP/ATP translocase 3 OS=Homo sapiens GN=SLC25A6 PE=1 SV=4 - [ADT3_HUMAN]	19,13	4
P61204	ADP-ribosylation factor 3 OS=Homo sapiens GN=ARF3 PE=1 SV=2 - [ARF3_HUMAN]	22,10	1
P18085	ADP-ribosylation factor 4 OS=Homo sapiens GN=ARF4 PE=1 SV=3 - [ARF4_HUMAN]	11,67	1
P84085	ADP-ribosylation factor 5 OS=Homo sapiens GN=ARF5 PE=1 SV=2 - [ARF5_HUMAN]	11,67	1
P62330	ADP-ribosylation factor 6 OS=Homo sapiens GN=ARF6 PE=1 SV=2 - [ARF6_HUMAN]	6,29	1
Q15041	ADP-ribosylation factor-like protein 6-interacting protein 1 OS=Homo sapiens GN=ARL6IP1 PE=1 SV=2 - [AR6P1_HUMAN]	3,94	1
Q9NVJ2	ADP-ribosylation factor-like protein 8B OS=Homo sapiens GN=ARL8B PE=1 SV=1 - [ARL8B_HUMAN]	23,12	1
Q9UKK9	ADP-sugar pyrophosphatase OS=Homo sapiens GN=NUDT5 PE=1 SV=1 - [NUDT5_HUMAN]	6,85	1
Q9Y4W6	AFG3-like protein 2 OS=Homo sapiens GN=AFG3L2 PE=1 SV=2 - [AFG32_HUMAN]	2,13	2
O43488	Aflatoxin B1 aldehyde reductase member 2 OS=Homo sapiens GN=AKR7A2 PE=1 SV=3 - [ARK72_HUMAN]	3,34	1
Q8IZ83	Aldehyde dehydrogenase family 16 member A1 OS=Homo sapiens GN=ALDH16A1 PE=1 SV=2 - [A16A1_HUMAN]	1,37	1
P05186	Alkaline phosphatase, tissue-nonspecific isozyme OS=Homo sapiens GN=ALPL PE=1 SV=4 - [PPBT_HUMAN]	4,39	2
O00116	Alkyldihydroxyacetonephosphate synthase, peroxisomal OS=Homo sapiens GN=AGPS PE=1 SV=1 - [ADAS_HUMAN]	5,47	2
Q9BYC5	Alpha-(1,6)-fucosyltransferase OS=Homo sapiens GN=FUT8 PE=1 SV=2 - [FUT8_HUMAN]	10,09	3
P01011	Alpha-1-antichymotrypsin OS=Homo sapiens GN=SERPINA3 PE=1 SV=2 - [AACT_HUMAN]	15,13	4
P01009	Alpha-1-antitrypsin OS=Homo sapiens GN=SERPINA1 PE=1 SV=3 - [A1AT_HUMAN]	41,15	9
P04217	Alpha-1B-glycoprotein OS=Homo sapiens GN=A1BG PE=1 SV=4 - [A1BG_HUMAN]	8,89	3
P08697	Alpha-2-antiplasmin OS=Homo sapiens GN=SERPINF2 PE=1 SV=3 - [A2AP_HUMAN]	4,28	2
P02765	Alpha-2-HS-glycoprotein OS=Homo sapiens GN=AHSG PE=1 SV=1 - [FETUA_HUMAN]	15,80	5
P01023	Alpha-2-macroglobulin OS=Homo sapiens GN=A2M PE=1 SV=3 - [A2MG_HUMAN]	51,09	26
A8K2U0	Alpha-2-macroglobulin-like protein 1 OS=Homo sapiens GN=A2ML1 PE=1 SV=3 - [A2ML1_HUMAN]	0,89	1
P12814	Alpha-actinin-1 OS=Homo sapiens GN=ACTN1 PE=1 SV=2 - [ACTN1_HUMAN]	44,62	19
O43707	Alpha-actinin-4 OS=Homo sapiens GN=ACTN4 PE=1 SV=2 - [ACTN4_HUMAN]	35,02	11

P35611	Alpha-adducin OS=Homo sapiens GN=ADD1 PE=1 SV=2 - [ADDA_HUMAN]	4,48	2
P04745	Alpha-amylase 1 OS=Homo sapiens GN=AMY1A PE=1 SV=2 - [AMY1_HUMAN]	4,70	2
P06733	Alpha-enolase OS=Homo sapiens GN=ENO1 PE=1 SV=2 - [ENOA_HUMAN]	37,56	7
Q16706	Alpha-mannosidase 2 OS=Homo sapiens GN=MAN2A1 PE=1 SV=2 - [MA2A1_HUMAN]	2,45	1
P49641	Alpha-mannosidase 2x OS=Homo sapiens GN=MAN2A2 PE=2 SV=3 - [MA2A2_HUMAN]	2,87	1
P17050	Alpha-N-acetylgalactosaminidase OS=Homo sapiens GN=NAGA PE=1 SV=2 - [NAGAB_HUMAN]	5,60	1
Q9NSC7	Alpha-N-acetylgalactosaminide alpha-2,6-sialyltransferase 1 OS=Homo sapiens GN=ST6GALNAC1 PE=2 SV=1 - [SIA7A_HUMAN]	1,50	1
P54920	Alpha-soluble NSF attachment protein OS=Homo sapiens GN=NAPA PE=1 SV=3 - [SNAA_HUMAN]	7,46	1
P37840	Alpha-synuclein OS=Homo sapiens GN=SNCA PE=1 SV=1 - [SYUA_HUMAN]	11,43	1
P27338	Amine oxidase [flavin-containing] B OS=Homo sapiens GN=MAOB PE=1 SV=3 - [AOFB_HUMAN]	22,50	6
P15144	Aminopeptidase N OS=Homo sapiens GN=ANPEP PE=1 SV=4 - [AMPN_HUMAN]	18,92	3
P05067	Amyloid beta A4 protein OS=Homo sapiens GN=APP PE=1 SV=3 - [A4_HUMAN]	2,47	2
Q9Y679	Ancient ubiquitous protein 1 OS=Homo sapiens GN=AUP1 PE=1 SV=1 - [AUP1_HUMAN]	2,52	1
Q15389	Angiopoietin-1 OS=Homo sapiens GN=ANGPT1 PE=1 SV=2 - [ANGP1_HUMAN]	6,43	2
Q8NI99	Angiopoietin-related protein 6 OS=Homo sapiens GN=ANGPTL6 PE=1 SV=1 - [ANGL6_HUMAN]	2,77	1
P01019	Angiotensinogen OS=Homo sapiens GN=AGT PE=1 SV=1 - [ANGT_HUMAN]	11,13	2
P04920	Anion exchange protein 2 OS=Homo sapiens GN=SLC4A2 PE=1 SV=4 - [B3A2_HUMAN]	2,90	1
P16157	Ankyrin-1 OS=Homo sapiens GN=ANK1 PE=1 SV=3 - [ANK1_HUMAN]	10,47	11
P50995	Annexin A11 OS=Homo sapiens GN=ANXA11 PE=1 SV=1 - [ANX11_HUMAN]	11,68	3
P07355	Annexin A2 OS=Homo sapiens GN=ANXA2 PE=1 SV=2 - [ANXA2_HUMAN]	25,37	4
P09525	Annexin A4 OS=Homo sapiens GN=ANXA4 PE=1 SV=4 - [ANXA4_HUMAN]	21,94	1
P20073	Annexin A7 OS=Homo sapiens GN=ANXA7 PE=1 SV=3 - [ANXA7_HUMAN]	19,47	1
Q9NW15	Anoctamin-10 OS=Homo sapiens GN=ANO10 PE=1 SV=2 - [ANO10_HUMAN]	4,24	2
Q4KMQ2	Anoctamin-6 OS=Homo sapiens GN=ANO6 PE=1 SV=2 - [ANO6_HUMAN]	10,11	4
Q03518	Antigen peptide transporter 1 OS=Homo sapiens GN=TAP1 PE=1 SV=2 - [TAP1_HUMAN]	7,05	3
Q03519	Antigen peptide transporter 2 OS=Homo sapiens GN=TAP2 PE=1 SV=1 - [TAP2_HUMAN]	3,94	1
P01008	Antithrombin-III OS=Homo sapiens GN=SERPINC1 PE=1 SV=1 - [ANT3_HUMAN]	10,13	2
Q10567	AP-1 complex subunit beta-1 OS=Homo sapiens GN=AP1B1 PE=1 SV=2 - [AP1B1_HUMAN]	4,64	3
P61966	AP-1 complex subunit sigma-1A OS=Homo sapiens GN=AP1S1 PE=1 SV=1 - [AP1S1_HUMAN]	10,13	1
O95782	AP-2 complex subunit alpha-1 OS=Homo sapiens GN=AP2A1 PE=1 SV=3 - [AP2A1_HUMAN]	8,80	6
P63010	AP-2 complex subunit beta OS=Homo sapiens GN=AP2B1 PE=1 SV=1 - [AP2B1_HUMAN]	3,31	2
Q96CW1	AP-2 complex subunit mu OS=Homo sapiens GN=AP2M1 PE=1 SV=2 - [AP2M1_HUMAN]	14,02	2
O00203	AP-3 complex subunit beta-1 OS=Homo sapiens GN=AP3B1 PE=1 SV=3 - [AP3B1_HUMAN]	2,74	3
P02647	Apolipoprotein A-I OS=Homo sapiens GN=APOA1 PE=1 SV=1 - [APOA1_HUMAN]	55,06	17
P02652	Apolipoprotein A-II OS=Homo sapiens GN=APOA2 PE=1 SV=1 - [APOA2_HUMAN]	19,00	3
P06727	Apolipoprotein A-IV OS=Homo sapiens GN=APOA4 PE=1 SV=3 - [APOA4_HUMAN]	26,01	7
P04114	Apolipoprotein B-100 OS=Homo sapiens GN=APOB PE=1 SV=2 - [APOB_HUMAN]	10,69	41
P02654	Apolipoprotein C-I OS=Homo sapiens GN=APOC1 PE=1 SV=1 - [APOC1_HUMAN]	37,35	3
P02655	Apolipoprotein C-II OS=Homo sapiens GN=APOC2 PE=1 SV=1 - [APOC2_HUMAN]	17,82	2
P02656	Apolipoprotein C-III OS=Homo sapiens GN=APOC3 PE=1 SV=1 - [APOC3_HUMAN]	16,16	1

P05090	Apolipoprotein D OS=Homo sapiens GN=APOD PE=1 SV=1 - [APOD_HUMAN]	34,92	7
P02649	Apolipoprotein E OS=Homo sapiens GN=APOE PE=1 SV=1 - [APOE_HUMAN]	40,06	12
O14791	Apolipoprotein L1 OS=Homo sapiens GN=APOL1 PE=1 SV=5 - [APOL1_HUMAN]	25,13	7
O95445	Apolipoprotein M OS=Homo sapiens GN=APOM PE=1 SV=2 - [APOM_HUMAN]	12,23	3
P08519	Apolipoprotein(a) OS=Homo sapiens GN=LPA PE=1 SV=1 - [APOA_HUMAN]	6,82	1
Q07812	Apoptosis regulator BAX OS=Homo sapiens GN=BAX PE=1 SV=1 - [BAX_HUMAN]	22,92	3
O95831	Apoptosis-inducing factor 1, mitochondrial OS=Homo sapiens GN=AIFM1 PE=1 SV=1 - [AIFM1_HUMAN]	20,39	5
P18054	Arachidonate 12-lipoxygenase, 12S-type OS=Homo sapiens GN=ALOX12 PE=1 SV=4 - [LOX12_HUMAN]	19,61	2
Q9UH62	Armadillo repeat-containing X-linked protein 3 OS=Homo sapiens GN=ARMCX3 PE=1 SV=1 - [ARMX3_HUMAN]	12,93	3
P00505	Aspartate aminotransferase, mitochondrial OS=Homo sapiens GN=GOT2 PE=1 SV=3 - [AATM_HUMAN]	13,72	5
Q12797	Aspartyl/asparaginyl beta-hydroxylase OS=Homo sapiens GN=ASPH PE=1 SV=3 - [ASPH_HUMAN]	3,17	2
Q8WXF7	Atlastin-1 OS=Homo sapiens GN=ATL1 PE=1 SV=1 - [ATLA1_HUMAN]	5,56	3
Q6DD88	Atlastin-3 OS=Homo sapiens GN=ATL3 PE=1 SV=1 - [ATLA3_HUMAN]	12,75	4
P00846	ATP synthase subunit a OS=Homo sapiens GN=MT-ATP6 PE=1 SV=1 - [ATP6_HUMAN]	4,42	1
P25705	ATP synthase subunit alpha, mitochondrial OS=Homo sapiens GN=ATP5A1 PE=1 SV=1 - [ATPA_HUMAN]	32,55	16
P24539	ATP synthase subunit b, mitochondrial OS=Homo sapiens GN=ATP5F1 PE=1 SV=2 - [AT5F1_HUMAN]	14,84	2
P06576	ATP synthase subunit beta, mitochondrial OS=Homo sapiens GN=ATP5B PE=1 SV=3 - [ATPB_HUMAN]	32,70	12
O75947	ATP synthase subunit d, mitochondrial OS=Homo sapiens GN=ATP5H PE=1 SV=3 - [ATP5H_HUMAN]	18,01	2
P30049	ATP synthase subunit delta, mitochondrial OS=Homo sapiens GN=ATP5D PE=1 SV=2 - [ATPD_HUMAN]	13,69	2
P56385	ATP synthase subunit e, mitochondrial OS=Homo sapiens GN=ATP5I PE=1 SV=2 - [ATP5I_HUMAN]	31,88	3
P56381	ATP synthase subunit epsilon, mitochondrial OS=Homo sapiens GN=ATP5E PE=1 SV=2 - [ATP5E_HUMAN]	15,69	1
O75964	ATP synthase subunit g, mitochondrial OS=Homo sapiens GN=ATP5L PE=1 SV=3 - [ATP5L_HUMAN]	24,27	1
P36542	ATP synthase subunit gamma, mitochondrial OS=Homo sapiens GN=ATP5C1 PE=1 SV=1 - [ATPG_HUMAN]	14,77	2
P48047	ATP synthase subunit O, mitochondrial OS=Homo sapiens GN=ATP5O PE=1 SV=1 - [ATPO_HUMAN]	8,92	2
P18859	ATP synthase-coupling factor 6, mitochondrial OS=Homo sapiens GN=ATP5J PE=1 SV=1 - [ATP5J_HUMAN]	17,59	1
Q9NVI7	ATPase family AAA domain-containing protein 3A OS=Homo sapiens GN=ATAD3A PE=1 SV=2 - [ATD3A_HUMAN]	6,94	3
Q9NRK6	ATP-binding cassette sub-family B member 10, mitochondrial OS=Homo sapiens GN=ABCB10 PE=1 SV=2 - [ABCBA_HUMAN]	1,49	1
Q9NP58	ATP-binding cassette sub-family B member 6, mitochondrial OS=Homo sapiens GN=ABCB6 PE=1 SV=1 - [ABCB6_HUMAN]	8,08	2
P33897	ATP-binding cassette sub-family D member 1 OS=Homo sapiens GN=ABCD1 PE=1 SV=2 - [ABCD1_HUMAN]	1,88	1
O75882	Attractin OS=Homo sapiens GN=ATRN PE=1 SV=2 - [ATRN_HUMAN]	0,70	1
Q7Z3C6	Autophagy-related protein 9A OS=Homo sapiens GN=ATG9A PE=1 SV=3 - [ATG9A_HUMAN]	2,15	1
O95816	BAG family molecular chaperone regulator 2 OS=Homo sapiens GN=BAG2 PE=1 SV=1 - [BAG2_HUMAN]	4,27	1
P02730	Band 3 anion transport protein OS=Homo sapiens GN=SLC4A1 PE=1 SV=3 - [B3AT_HUMAN]	18,44	12
P35613	Basigin OS=Homo sapiens GN=BSG PE=1 SV=2 - [BASI_HUMAN]	21,04	2
P51572	B-cell receptor-associated protein 31 OS=Homo sapiens GN=BCAP31 PE=1 SV=3 - [BAP31_HUMAN]	31,30	8
Q07817	Bcl-2-like protein 1 OS=Homo sapiens GN=BCL2L1 PE=1 SV=1 - [B2CL1_HUMAN]	3,86	1
P02749	Beta-2-glycoprotein 1 OS=Homo sapiens GN=APOH PE=1 SV=3 - [APOH_HUMAN]	21,45	3
P42025	Beta-centractin OS=Homo sapiens GN=ACTR1B PE=1 SV=1 - [ACTY_HUMAN]	4,52	2
Q9HBI1	Beta-parvin OS=Homo sapiens GN=PARVB PE=1 SV=1 - [PARVB_HUMAN]	21,43	4
P07814	Bifunctional glutamate/proline--tRNA ligase OS=Homo sapiens GN=EPRS PE=1 SV=5 - [SYEP_HUMAN]	0,73	1

Q10589	Bone marrow stromal antigen 2 OS=Homo sapiens GN=BST2 PE=1 SV=1 - [BST2_HUMAN]	5,56	1
P80723	Brain acid soluble protein 1 OS=Homo sapiens GN=BASP1 PE=1 SV=2 - [BASP1_HUMAN]	40,97	5
P23560	Brain-derived neurotrophic factor OS=Homo sapiens GN=BDNF PE=1 SV=1 - [BDNF_HUMAN]	6,07	1
Q8WY22	BRI3-binding protein OS=Homo sapiens GN=BRI3BP PE=1 SV=1 - [BRI3B_HUMAN]	6,77	1
Q96CX2	BTB/POZ domain-containing protein KCTD12 OS=Homo sapiens GN=KCTD12 PE=1 SV=1 - [KCD12_HUMAN]	14,77	2
O14523	C2 domain-containing protein 2-like OS=Homo sapiens GN=C2CD2L PE=1 SV=3 - [C2C2L_HUMAN]	4,25	2
P04003	C4b-binding protein alpha chain OS=Homo sapiens GN=C4BPA PE=1 SV=2 - [C4BPA_HUMAN]	29,98	4
P20851	C4b-binding protein beta chain OS=Homo sapiens GN=C4BPB PE=1 SV=1 - [C4BPB_HUMAN]	4,76	1
O75844	CAAX prenyl protease 1 homolog OS=Homo sapiens GN=ZMPSTE24 PE=1 SV=2 - [FACE1_HUMAN]	10,53	4
Q99653	Calcineurin B homologous protein 1 OS=Homo sapiens GN=CHP1 PE=1 SV=3 - [CHP1_HUMAN]	26,15	1
Q96BS2	Calcineurin B homologous protein 3 OS=Homo sapiens GN=TESC PE=1 SV=3 - [CHP3_HUMAN]	32,24	2
Q8NE86	Calcium uniporter protein, mitochondrial OS=Homo sapiens GN=MCU PE=1 SV=1 - [MCU_HUMAN]	12,25	2
Q9UJS0	Calcium-binding mitochondrial carrier protein Aralar2 OS=Homo sapiens GN=SLC25A13 PE=1 SV=2 - [CMC2_HUMAN]	1,19	1
Q6NUK1	Calcium-binding mitochondrial carrier protein SCaMC-1 OS=Homo sapiens GN=SLC25A24 PE=1 SV=2 - [SCMC1_HUMAN]	8,81	1
P98194	Calcium-transporting ATPase type 2C member 1 OS=Homo sapiens GN=ATP2C1 PE=1 SV=3 - [AT2C1_HUMAN]	6,31	5
Q05682	Caldesmon OS=Homo sapiens GN=CALD1 PE=1 SV=3 - [CALD1_HUMAN]	11,22	7
P62158	Calmodulin OS=Homo sapiens GN=CALM1 PE=1 SV=2 - [CALM_HUMAN]	35,57	2
Q9NZT1	Calmodulin-like protein 5 OS=Homo sapiens GN=CALML5 PE=1 SV=2 - [CALL5_HUMAN]	32,88	2
P27824	Calnexin OS=Homo sapiens GN=CANX PE=1 SV=2 - [CALX_HUMAN]	20,61	8
P04632	Calpain small subunit 1 OS=Homo sapiens GN=CAPNS1 PE=1 SV=1 - [CPNS1_HUMAN]	16,79	1
P07384	Calpain-1 catalytic subunit OS=Homo sapiens GN=CAPN1 PE=1 SV=1 - [CAN1_HUMAN]	18,35	6
Q99439	Calponin-2 OS=Homo sapiens GN=CNN2 PE=1 SV=4 - [CNN2_HUMAN]	10,36	3
P27797	Calreticulin OS=Homo sapiens GN=CALR PE=1 SV=1 - [CALR_HUMAN]	23,74	3
P22694	cAMP-dependent protein kinase catalytic subunit beta OS=Homo sapiens GN=PRKACB PE=1 SV=2 - [KAPCB_HUMAN]	9,69	4
P10644	cAMP-dependent protein kinase type I-alpha regulatory subunit OS=Homo sapiens GN=PRKAR1A PE=1 SV=1 - [KAP0_HUMAN]	32,28	6
P00915	Carbonic anhydrase 1 OS=Homo sapiens GN=CA1 PE=1 SV=2 - [CAH1_HUMAN]	7,28	1
Q9UI42	Carboxypeptidase A4 OS=Homo sapiens GN=CPA4 PE=1 SV=2 - [CBPA4_HUMAN]	4,75	2
P22792	Carboxypeptidase N subunit 2 OS=Homo sapiens GN=CPN2 PE=1 SV=3 - [CPN2_HUMAN]	1,83	1
Q9GZU7	Carboxy-terminal domain RNA polymerase II polypeptide A small phosphatase 1 OS=Homo sapiens GN=CTDSP1 PE=1 SV=1 - [CTI	3,45	1
Q8N3K9	Cardiomyopathy-associated protein 5 OS=Homo sapiens GN=CMYA5 PE=1 SV=3 - [CMYA5_HUMAN]	0,17	1
P43155	Carnitine O-acetyltransferase OS=Homo sapiens GN=CRAT PE=1 SV=5 - [CACP_HUMAN]	3,35	2
P50416	Carnitine O-palmitoyltransferase 1, liver isoform OS=Homo sapiens GN=CPT1A PE=1 SV=2 - [CPT1A_HUMAN]	15,14	7
P23786	Carnitine O-palmitoyltransferase 2, mitochondrial OS=Homo sapiens GN=CPT2 PE=1 SV=2 - [CPT2_HUMAN]	1,52	1
P49747	Cartilage oligomeric matrix protein OS=Homo sapiens GN=COMP PE=1 SV=2 - [COMP_HUMAN]	5,42	3
P31944	Caspase-14 OS=Homo sapiens GN=CASP14 PE=1 SV=2 - [CASPE_HUMAN]	15,29	2
P04040	Catalase OS=Homo sapiens GN=CAT PE=1 SV=3 - [CATA_HUMAN]	15,56	5
P21964	Catechol O-methyltransferase OS=Homo sapiens GN=COMT PE=1 SV=2 - [COMT_HUMAN]	2,95	1
P07858	Cathepsin B OS=Homo sapiens GN=CTSB PE=1 SV=3 - [CATB_HUMAN]	3,24	1
P07339	Cathepsin D OS=Homo sapiens GN=CTSD PE=1 SV=1 - [CATD_HUMAN]	15,53	5
P08311	Cathepsin G OS=Homo sapiens GN=CTSG PE=1 SV=2 - [CATG_HUMAN]	3,53	1

P56202	Cathepsin W OS=Homo sapiens GN=CTSW PE=1 SV=2 - [CATW_HUMAN]	3,19	1
P20645	Cation-dependent mannose-6-phosphate receptor OS=Homo sapiens GN=M6PR PE=1 SV=1 - [MPRD_HUMAN]	15,52	3
P13501	C-C motif chemokine 5 OS=Homo sapiens GN=CCL5 PE=1 SV=3 - [CCL5_HUMAN]	8,79	1
Q6YHK3	CD109 antigen OS=Homo sapiens GN=CD109 PE=1 SV=2 - [CD109_HUMAN]	4,50	2
P48509	CD151 antigen OS=Homo sapiens GN=CD151 PE=1 SV=3 - [CD151_HUMAN]	19,37	2
Q9NNX6	CD209 antigen OS=Homo sapiens GN=CD209 PE=1 SV=1 - [CD209_HUMAN]	13,37	1
P29965	CD40 ligand OS=Homo sapiens GN=CD40LG PE=1 SV=1 - [CD40L_HUMAN]	3,83	1
P16070	CD44 antigen OS=Homo sapiens GN=CD44 PE=1 SV=3 - [CD44_HUMAN]	2,16	1
O43866	CD5 antigen-like OS=Homo sapiens GN=CD5L PE=1 SV=1 - [CD5L_HUMAN]	35,45	6
P13987	CD59 glycoprotein OS=Homo sapiens GN=CD59 PE=1 SV=1 - [CD59_HUMAN]	15,63	1
P08962	CD63 antigen OS=Homo sapiens GN=CD63 PE=1 SV=2 - [CD63_HUMAN]	7,56	2
P60033	CD81 antigen OS=Homo sapiens GN=CD81 PE=1 SV=1 - [CD81_HUMAN]	8,47	1
P27701	CD82 antigen OS=Homo sapiens GN=CD82 PE=1 SV=1 - [CD82_HUMAN]	3,75	1
P21926	CD9 antigen OS=Homo sapiens GN=CD9 PE=1 SV=4 - [CD9_HUMAN]	13,60	3
P14209	CD99 antigen OS=Homo sapiens GN=CD99 PE=1 SV=1 - [CD99_HUMAN]	7,57	1
Q96JB5	CDK5 regulatory subunit-associated protein 3 OS=Homo sapiens GN=CDK5RAP3 PE=1 SV=2 - [CK5P3_HUMAN]	2,77	1
O14735	CDP-diacylglycerol--inositol 3-phosphatidyltransferase OS=Homo sapiens GN=CDIPT PE=1 SV=1 - [CDIPT_HUMAN]	9,86	2
Q9NV96	Cell cycle control protein 50A OS=Homo sapiens GN=TMEM30A PE=1 SV=1 - [CC50A_HUMAN]	17,73	1
P60953	Cell division control protein 42 homolog OS=Homo sapiens GN=CDC42 PE=1 SV=2 - [CDC42_HUMAN]	10,99	2
Q96G23	Ceramide synthase 2 OS=Homo sapiens GN=CERS2 PE=1 SV=1 - [CERS2_HUMAN]	2,37	1
P00450	Ceruloplasmin OS=Homo sapiens GN=CP PE=1 SV=1 - [CERU_HUMAN]	15,77	9
O76074	cGMP-specific 3',5'-cyclic phosphodiesterase OS=Homo sapiens GN=PDE5A PE=1 SV=2 - [PDE5A_HUMAN]	2,40	2
Q9H444	Charged multivesicular body protein 4b OS=Homo sapiens GN=CHMP4B PE=1 SV=1 - [CHM4B_HUMAN]	13,39	1
Q96FZ7	Charged multivesicular body protein 6 OS=Homo sapiens GN=CHMP6 PE=1 SV=3 - [CHMP6_HUMAN]	3,48	1
O00299	Chloride intracellular channel protein 1 OS=Homo sapiens GN=CLIC1 PE=1 SV=4 - [CLIC1_HUMAN]	31,95	4
Q8WWI5	Choline transporter-like protein 1 OS=Homo sapiens GN=SLC44A1 PE=1 SV=1 - [CTL1_HUMAN]	11,42	3
O75390	Citrate synthase, mitochondrial OS=Homo sapiens GN=CS PE=1 SV=2 - [CISY_HUMAN]	22,75	5
Q96DZ9	CKLF-like MARVEL transmembrane domain-containing protein 5 OS=Homo sapiens GN=CMTM5 PE=2 SV=2 - [CKLF5_HUMAN]	8,97	2
Q00610	Clathrin heavy chain 1 OS=Homo sapiens GN=CLTC PE=1 SV=5 - [CLH1_HUMAN]	16,90	10
Q14677	Clathrin interactor 1 OS=Homo sapiens GN=CLINT1 PE=1 SV=1 - [EPN4_HUMAN]	12,00	5
P09496	Clathrin light chain A OS=Homo sapiens GN=CLTA PE=1 SV=1 - [CLCA_HUMAN]	6,45	2
P09497	Clathrin light chain B OS=Homo sapiens GN=CLTB PE=1 SV=1 - [CLCB_HUMAN]	11,35	3
O15551	Claudin-3 OS=Homo sapiens GN=CLDN3 PE=1 SV=1 - [CLD3_HUMAN]	11,82	1
O00501	Claudin-5 OS=Homo sapiens GN=CLDN5 PE=1 SV=1 - [CLD5_HUMAN]	12,84	2
O96005	Cleft lip and palate transmembrane protein 1 OS=Homo sapiens GN=CLPTM1 PE=1 SV=1 - [CLPT1_HUMAN]	2,24	1
P10909	Clusterin OS=Homo sapiens GN=CLU PE=1 SV=1 - [CLUS_HUMAN]	22,27	8
Q8IY22	C-Maf-inducing protein OS=Homo sapiens GN=CMIP PE=1 SV=3 - [CMIP_HUMAN]	1,03	1
Q14019	Coactosin-like protein OS=Homo sapiens GN=COTL1 PE=1 SV=3 - [COTL1_HUMAN]	16,20	1
P12259	Coagulation factor V OS=Homo sapiens GN=F5 PE=1 SV=4 - [FA5_HUMAN]	11,29	16
P00451	Coagulation factor VIII OS=Homo sapiens GN=F8 PE=1 SV=1 - [FA8_HUMAN]	0,43	1

P00748	Coagulation factor XII OS=Homo sapiens GN=F12 PE=1 SV=3 - [FA12_HUMAN]	1,46	1
P00488	Coagulation factor XIII A chain OS=Homo sapiens GN=F13A1 PE=1 SV=4 - [F13A_HUMAN]	25,82	11
P53621	Coatomer subunit alpha OS=Homo sapiens GN=COPA PE=1 SV=2 - [COPA_HUMAN]	6,54	5
P53618	Coatomer subunit beta OS=Homo sapiens GN=COPB1 PE=1 SV=3 - [COPB_HUMAN]	1,89	1
P35606	Coatomer subunit beta' OS=Homo sapiens GN=COPB2 PE=1 SV=2 - [COPB2_HUMAN]	8,39	5
O43405	Cochlin OS=Homo sapiens GN=COCH PE=1 SV=1 - [COCH_HUMAN]	1,82	1
P23528	Cofilin-1 OS=Homo sapiens GN=CFL1 PE=1 SV=3 - [COF1_HUMAN]	39,16	4
Q9NX63	Coiled-coil-helix-coiled-coil-helix domain-containing protein 3, mitochondrial OS=Homo sapiens GN=CHCHD3 PE=1 SV=1 - [CHCH3_	14,98	3
P12110	Collagen alpha-2(VI) chain OS=Homo sapiens GN=COL6A2 PE=1 SV=4 - [CO6A2_HUMAN]	1,08	1
P12111	Collagen alpha-3(VI) chain OS=Homo sapiens GN=COL6A3 PE=1 SV=5 - [CO6A3_HUMAN]	7,96	4
P02745	Complement C1q subcomponent subunit A OS=Homo sapiens GN=C1QA PE=1 SV=2 - [C1QA_HUMAN]	4,49	1
P02746	Complement C1q subcomponent subunit B OS=Homo sapiens GN=C1QB PE=1 SV=3 - [C1QB_HUMAN]	23,32	2
P02747	Complement C1q subcomponent subunit C OS=Homo sapiens GN=C1QC PE=1 SV=3 - [C1QC_HUMAN]	37,14	4
P00736	Complement C1r subcomponent OS=Homo sapiens GN=C1R PE=1 SV=2 - [C1R_HUMAN]	30,07	9
P09871	Complement C1s subcomponent OS=Homo sapiens GN=C1S PE=1 SV=1 - [C1S_HUMAN]	3,92	3
P06681	Complement C2 OS=Homo sapiens GN=C2 PE=1 SV=2 - [CO2_HUMAN]	1,60	1
P01024	Complement C3 OS=Homo sapiens GN=C3 PE=1 SV=2 - [CO3_HUMAN]	41,91	34
P0C0L5	Complement C4-B OS=Homo sapiens GN=C4B PE=1 SV=2 - [CO4B_HUMAN]	18,98	16
P01031	Complement C5 OS=Homo sapiens GN=C5 PE=1 SV=4 - [CO5_HUMAN]	9,01	3
P13671	Complement component C6 OS=Homo sapiens GN=C6 PE=1 SV=3 - [CO6_HUMAN]	1,93	1
P10643	Complement component C7 OS=Homo sapiens GN=C7 PE=1 SV=2 - [CO7_HUMAN]	2,49	1
P07357	Complement component C8 alpha chain OS=Homo sapiens GN=C8A PE=1 SV=2 - [CO8A_HUMAN]	4,62	1
P07358	Complement component C8 beta chain OS=Homo sapiens GN=C8B PE=1 SV=3 - [CO8B_HUMAN]	1,35	1
P02748	Complement component C9 OS=Homo sapiens GN=C9 PE=1 SV=2 - [CO9_HUMAN]	6,08	2
P08174	Complement decay-accelerating factor OS=Homo sapiens GN=CD55 PE=1 SV=4 - [DAF_HUMAN]	9,71	3
P00751	Complement factor B OS=Homo sapiens GN=CFB PE=1 SV=2 - [CFAB_HUMAN]	9,95	7
P08603	Complement factor H OS=Homo sapiens GN=CFH PE=1 SV=4 - [CFAH_HUMAN]	13,97	4
Q03591	Complement factor H-related protein 1 OS=Homo sapiens GN=CFHR1 PE=1 SV=2 - [FHR1_HUMAN]	13,64	3
Q6PJW8	Consortin OS=Homo sapiens GN=CNST PE=1 SV=3 - [CNST_HUMAN]	3,72	2
Q92905	COP9 signalosome complex subunit 5 OS=Homo sapiens GN=COPS5 PE=1 SV=4 - [CSN5_HUMAN]	2,99	1
P31146	Coronin-1A OS=Homo sapiens GN=CORO1A PE=1 SV=4 - [COR1A_HUMAN]	7,59	2
Q9BR76	Coronin-1B OS=Homo sapiens GN=CORO1B PE=1 SV=1 - [COR1B_HUMAN]	3,48	1
Q9ULV4	Coronin-1C OS=Homo sapiens GN=CORO1C PE=1 SV=1 - [COR1C_HUMAN]	8,23	2
P08185	Corticosteroid-binding globulin OS=Homo sapiens GN=SERPINA6 PE=1 SV=1 - [CBG_HUMAN]	1,73	1
O15320	cTAGE family member 5 OS=Homo sapiens GN=CTAGE5 PE=1 SV=4 - [CTGE5_HUMAN]	1,74	1
O15194	CTD small phosphatase-like protein OS=Homo sapiens GN=CTDSPL PE=1 SV=2 - [CTDSL_HUMAN]	3,99	1
Q9P126	C-type lectin domain family 1 member B OS=Homo sapiens GN=CLEC1B PE=1 SV=2 - [CLC1B_HUMAN]	6,99	1
P61073	C-X-C chemokine receptor type 4 OS=Homo sapiens GN=CXCR4 PE=1 SV=1 - [CXCR4_HUMAN]	12,78	1
P01040	Cystatin-A OS=Homo sapiens GN=CSTA PE=1 SV=1 - [CYTA_HUMAN]	48,98	4
Q15828	Cystatin-M OS=Homo sapiens GN=CST6 PE=1 SV=1 - [CYTM_HUMAN]	7,38	1

P21291	Cysteine and glycine-rich protein 1 OS=Homo sapiens GN=CSRP1 PE=1 SV=3 - [CSRP1_HUMAN]	5,18	1
Q96HD1	Cysteine-rich with EGF-like domain protein 1 OS=Homo sapiens GN=CRELD1 PE=1 SV=3 - [CREL1_HUMAN]	9,29	1
P04839	Cytochrome b-245 heavy chain OS=Homo sapiens GN=CYBB PE=1 SV=2 - [CY24B_HUMAN]	1,93	1
P31930	Cytochrome b-c1 complex subunit 1, mitochondrial OS=Homo sapiens GN=UQCRC1 PE=1 SV=3 - [QCR1_HUMAN]	4,17	2
P22695	Cytochrome b-c1 complex subunit 2, mitochondrial OS=Homo sapiens GN=UQCRC2 PE=1 SV=3 - [QCR2_HUMAN]	11,70	4
O14949	Cytochrome b-c1 complex subunit 8 OS=Homo sapiens GN=UQCRQ PE=1 SV=4 - [QCR8_HUMAN]	8,54	1
P47985	Cytochrome b-c1 complex subunit Rieske, mitochondrial OS=Homo sapiens GN=UQCRFS1 PE=1 SV=2 - [UCRI_HUMAN]	10,58	3
P99999	Cytochrome c OS=Homo sapiens GN=CYCS PE=1 SV=2 - [CYC_HUMAN]	38,10	1
P00403	Cytochrome c oxidase subunit 2 OS=Homo sapiens GN=MT-CO2 PE=1 SV=1 - [COX2_HUMAN]	14,54	3
P13073	Cytochrome c oxidase subunit 4 isoform 1, mitochondrial OS=Homo sapiens GN=COX4I1 PE=1 SV=1 - [COX41_HUMAN]	31,95	4
P20674	Cytochrome c oxidase subunit 5A, mitochondrial OS=Homo sapiens GN=COX5A PE=1 SV=2 - [COX5A_HUMAN]	21,33	2
P10606	Cytochrome c oxidase subunit 5B, mitochondrial OS=Homo sapiens GN=COX5B PE=1 SV=2 - [COX5B_HUMAN]	15,50	2
P14854	Cytochrome c oxidase subunit 6B1 OS=Homo sapiens GN=COX6B1 PE=1 SV=2 - [CX6B1_HUMAN]	19,77	2
P09669	Cytochrome c oxidase subunit 6C OS=Homo sapiens GN=COX6C PE=1 SV=2 - [COX6C_HUMAN]	20,00	1
P14406	Cytochrome c oxidase subunit 7A2, mitochondrial OS=Homo sapiens GN=COX7A2 PE=1 SV=1 - [CX7A2_HUMAN]	27,71	1
O14548	Cytochrome c oxidase subunit 7A-related protein, mitochondrial OS=Homo sapiens GN=COX7A2L PE=1 SV=2 - [COX7R_HUMAN]	11,40	1
Q6UW02	Cytochrome P450 20A1 OS=Homo sapiens GN=CYP20A1 PE=2 SV=1 - [CP20A_HUMAN]	8,44	1
Q7L576	Cytoplasmic FMR1-interacting protein 1 OS=Homo sapiens GN=CYFIP1 PE=1 SV=1 - [CYFP1_HUMAN]	9,98	8
O43639	Cytoplasmic protein NCK2 OS=Homo sapiens GN=NCK2 PE=1 SV=2 - [NCK2_HUMAN]	2,89	1
P28838	Cytosol aminopeptidase OS=Homo sapiens GN=LAP3 PE=1 SV=3 - [AMPL_HUMAN]	2,31	1
Q96KP4	Cytosolic non-specific dipeptidase OS=Homo sapiens GN=CNDP2 PE=1 SV=2 - [CNDP2_HUMAN]	5,89	2
Q96HY6	DDRGK domain-containing protein 1 OS=Homo sapiens GN=DDRGK1 PE=1 SV=2 - [DDRGK_HUMAN]	4,14	1
Q9Y394	Dehydrogenase/reductase SDR family member 7 OS=Homo sapiens GN=DHRS7 PE=1 SV=1 - [DHRS7_HUMAN]	15,93	3
Q6IAN0	Dehydrogenase/reductase SDR family member 7B OS=Homo sapiens GN=DHRS7B PE=1 SV=2 - [DRS7B_HUMAN]	21,54	3
Q13011	Delta(3,5)-Delta(2,4)-dienoyl-CoA isomerase, mitochondrial OS=Homo sapiens GN=ECH1 PE=1 SV=2 - [ECH1_HUMAN]	11,59	3
P30038	Delta-1-pyrroline-5-carboxylate dehydrogenase, mitochondrial OS=Homo sapiens GN=ALDH4A1 PE=1 SV=3 - [AL4A1_HUMAN]	4,26	2
P49184	Deoxyribonuclease-1-like 1 OS=Homo sapiens GN=DNASE1L1 PE=1 SV=1 - [DNSL1_HUMAN]	8,61	1
P81605	Dermcidin OS=Homo sapiens GN=DCD PE=1 SV=2 - [DCD_HUMAN]	35,45	2
Q08554	Desmocollin-1 OS=Homo sapiens GN=DSC1 PE=1 SV=2 - [DSC1_HUMAN]	5,03	3
Q02413	Desmoglein-1 OS=Homo sapiens GN=DSG1 PE=1 SV=2 - [DSG1_HUMAN]	8,58	3
P15924	Desmoplakin OS=Homo sapiens GN=DSP PE=1 SV=3 - [DESP_HUMAN]	9,44	15
P60981	Destrin OS=Homo sapiens GN=DSTN PE=1 SV=3 - [DEST_HUMAN]	7,88	2
P09622	Dihydrolipoyl dehydrogenase, mitochondrial OS=Homo sapiens GN=DLD PE=1 SV=2 - [DLDH_HUMAN]	9,82	5
P10515	Dihydrolipoyllysine-residue acetyltransferase component of pyruvate dehydrogenase complex, mitochondrial OS=Homo sapiens GN=	2,63	1
P36957	Dihydrolipoyllysine-residue succinyltransferase component of 2-oxoglutarate dehydrogenase complex, mitochondrial OS=Homo sapi	18,54	5
Q9Y4D1	Disheveled-associated activator of morphogenesis 1 OS=Homo sapiens GN=DAAM1 PE=1 SV=2 - [DAAM1_HUMAN]	12,43	8
O14672	Disintegrin and metalloproteinase domain-containing protein 10 OS=Homo sapiens GN=ADAM10 PE=1 SV=1 - [ADA10_HUMAN]	16,71	8
Q9UBS4	DnaJ homolog subfamily B member 11 OS=Homo sapiens GN=DNAJB11 PE=1 SV=1 - [DJB11_HUMAN]	2,23	1
Q8IXB1	DnaJ homolog subfamily C member 10 OS=Homo sapiens GN=DNAJC10 PE=1 SV=2 - [DJC10_HUMAN]	2,14	1
Q13217	DnaJ homolog subfamily C member 3 OS=Homo sapiens GN=DNAJC3 PE=1 SV=1 - [DNJC3_HUMAN]	1,79	1

Q9P2X0	Dolichol-phosphate mannosyltransferase subunit 3 OS=Homo sapiens GN=DPM3 PE=1 SV=2 - [DPM3_HUMAN]	10,87	1
P39656	Dolichyl-diphosphooligosaccharide--protein glycosyltransferase 48 kDa subunit OS=Homo sapiens GN=DDOST PE=1 SV=4 - [OST48	14,47	2
P04843	Dolichyl-diphosphooligosaccharide--protein glycosyltransferase subunit 1 OS=Homo sapiens GN=RPN1 PE=1 SV=1 - [RPN1_HUMAN	35,91	10
P04844	Dolichyl-diphosphooligosaccharide--protein glycosyltransferase subunit 2 OS=Homo sapiens GN=RPN2 PE=1 SV=3 - [RPN2_HUMAN	13,79	4
P61803	Dolichyl-diphosphooligosaccharide--protein glycosyltransferase subunit DAD1 OS=Homo sapiens GN=DAD1 PE=1 SV=3 - [DAD1_HU	28,32	3
P46977	Dolichyl-diphosphooligosaccharide--protein glycosyltransferase subunit STT3A OS=Homo sapiens GN=STT3A PE=1 SV=2 - [STT3A_	7,80	3
Q8TCJ2	Dolichyl-diphosphooligosaccharide--protein glycosyltransferase subunit STT3B OS=Homo sapiens GN=STT3B PE=1 SV=1 - [STT3B_	6,05	2
Q9Y673	Dolichyl-phosphate beta-glucosyltransferase OS=Homo sapiens GN=ALG5 PE=1 SV=1 - [ALG5_HUMAN]	4,01	1
Q5BKT4	Dol-P-Glc:Glc(2)Man(9)GlcNAc(2)-PP-Dol alpha-1,2-glucosyltransferase OS=Homo sapiens GN=ALG10 PE=2 SV=1 - [AG10A_HUMAI	2,11	1
Q16643	Drebrin OS=Homo sapiens GN=DBN1 PE=1 SV=4 - [DREB_HUMAN]	8,01	4
Q9UJU6	Drebrin-like protein OS=Homo sapiens GN=DBNL PE=1 SV=1 - [DBNL_HUMAN]	3,72	1
Q8TEA8	D-tyrosyl-tRNA(Tyr) deacylase 1 OS=Homo sapiens GN=DTD1 PE=1 SV=2 - [DTD1_HUMAN]	14,83	2
Q9UN19	Dual adapter for phosphotyrosine and 3-phosphotyrosine and 3-phosphoinositide OS=Homo sapiens GN=DAPP1 PE=1 SV=1 - [DAF	3,57	1
Q14203	Dynactin subunit 1 OS=Homo sapiens GN=DCTN1 PE=1 SV=3 - [DCTN1_HUMAN]	2,66	3
Q13561	Dynactin subunit 2 OS=Homo sapiens GN=DCTN2 PE=1 SV=4 - [DCTN2_HUMAN]	6,98	2
P63167	Dynein light chain 1, cytoplasmic OS=Homo sapiens GN=DYNLL1 PE=1 SV=1 - [DYL1_HUMAN]	24,72	1
Q96K19	E3 ubiquitin-protein ligase RNF170 OS=Homo sapiens GN=RNF170 PE=1 SV=2 - [RN170_HUMAN]	5,43	1
Q5T4S7	E3 ubiquitin-protein ligase UBR4 OS=Homo sapiens GN=UBR4 PE=1 SV=1 - [UBR4_HUMAN]	1,06	2
Q6ZMW3	Echinoderm microtubule-associated protein-like 6 OS=Homo sapiens GN=EML6 PE=2 SV=2 - [EMAL6_HUMAN]	0,36	1
Q9H4M9	EH domain-containing protein 1 OS=Homo sapiens GN=EHD1 PE=1 SV=2 - [EHD1_HUMAN]	17,60	3
Q9NZN3	EH domain-containing protein 3 OS=Homo sapiens GN=EHD3 PE=1 SV=2 - [EHD3_HUMAN]	11,96	3
P13804	Electron transfer flavoprotein subunit alpha, mitochondrial OS=Homo sapiens GN=ETFA PE=1 SV=1 - [ETFA_HUMAN]	13,21	3
P38117	Electron transfer flavoprotein subunit beta OS=Homo sapiens GN=ETFB PE=1 SV=3 - [ETFB_HUMAN]	19,61	5
Q16134	Electron transfer flavoprotein-ubiquinone oxidoreductase, mitochondrial OS=Homo sapiens GN=ETFDH PE=1 SV=2 - [ETFD_HUMAI	1,94	1
P68104	Elongation factor 1-alpha 1 OS=Homo sapiens GN=EEF1A1 PE=1 SV=1 - [EF1A1_HUMAN]	6,06	3
P26641	Elongation factor 1-gamma OS=Homo sapiens GN=EEF1G PE=1 SV=3 - [EF1G_HUMAN]	5,26	1
P49411	Elongation factor Tu, mitochondrial OS=Homo sapiens GN=TUFM PE=1 SV=2 - [EFTU_HUMAN]	14,16	5
P50402	Emerin OS=Homo sapiens GN=EMD PE=1 SV=1 - [EMD_HUMAN]	24,02	4
Q9Y6C2	EMILIN-1 OS=Homo sapiens GN=EMILIN1 PE=1 SV=2 - [EMIL1_HUMAN]	16,83	14
P17813	Endoglin OS=Homo sapiens GN=ENG PE=1 SV=2 - [EGLN_HUMAN]	1,82	1
O94919	Endonuclease domain-containing 1 protein OS=Homo sapiens GN=ENDOD1 PE=1 SV=2 - [ENDD1_HUMAN]	16,80	6
Q99962	Endophilin-A1 OS=Homo sapiens GN=SH3GL2 PE=1 SV=1 - [SH3G2_HUMAN]	3,41	1
Q9NZ08	Endoplasmic reticulum aminopeptidase 1 OS=Homo sapiens GN=ERAP1 PE=1 SV=3 - [ERAP1_HUMAN]	5,53	1
Q7Z2K6	Endoplasmic reticulum metallopeptidase 1 OS=Homo sapiens GN=ERMP1 PE=1 SV=2 - [ERMP1_HUMAN]	4,54	2
P30040	Endoplasmic reticulum resident protein 29 OS=Homo sapiens GN=ERP29 PE=1 SV=4 - [ERP29_HUMAN]	19,92	1
Q9BS26	Endoplasmic reticulum resident protein 44 OS=Homo sapiens GN=ERP44 PE=1 SV=1 - [ERP44_HUMAN]	10,84	4
Q969X5	Endoplasmic reticulum-Golgi intermediate compartment protein 1 OS=Homo sapiens GN=ERGIC1 PE=1 SV=1 - [ERGI1_HUMAN]	6,55	2
P14625	Endoplasmin OS=Homo sapiens GN=HSP90B1 PE=1 SV=1 - [ENPL_HUMAN]	30,01	11
Q96AP7	Endothelial cell-selective adhesion molecule OS=Homo sapiens GN=ESAM PE=1 SV=1 - [ESAM_HUMAN]	15,38	3
P42892	Endothelin-converting enzyme 1 OS=Homo sapiens GN=ECE1 PE=1 SV=2 - [ECE1_HUMAN]	1,04	1

Q92556	Engulfment and cell motility protein 1 OS=Homo sapiens GN=ELMO1 PE=1 SV=2 - [ELMO1_HUMAN]	1,24	1
P42126	Enoyl-CoA delta isomerase 1, mitochondrial OS=Homo sapiens GN=ECI1 PE=1 SV=1 - [ECI1_HUMAN]	4,97	1
P98172	Ephrin-B1 OS=Homo sapiens GN=EFNB1 PE=1 SV=1 - [EFNB1_HUMAN]	14,74	2
P42566	Epidermal growth factor receptor substrate 15 OS=Homo sapiens GN=EPS15 PE=1 SV=2 - [EPS15_HUMAN]	2,12	1
Q9UBC2	Epidermal growth factor receptor substrate 15-like 1 OS=Homo sapiens GN=EPS15L1 PE=1 SV=1 - [EP15R_HUMAN]	2,20	1
Q8N766	ER membrane protein complex subunit 1 OS=Homo sapiens GN=EMC1 PE=1 SV=1 - [EMC1_HUMAN]	5,34	2
Q15006	ER membrane protein complex subunit 2 OS=Homo sapiens GN=EMC2 PE=1 SV=1 - [EMC2_HUMAN]	14,14	1
Q9NPA0	ER membrane protein complex subunit 7 OS=Homo sapiens GN=EMC7 PE=1 SV=1 - [EMC7_HUMAN]	4,13	1
O94905	Erlin-2 OS=Homo sapiens GN=ERLIN2 PE=1 SV=1 - [ERLN2_HUMAN]	22,71	3
P27105	Erythrocyte band 7 integral membrane protein OS=Homo sapiens GN=STOM PE=1 SV=3 - [STOM_HUMAN]	42,71	10
P16452	Erythrocyte membrane protein band 4.2 OS=Homo sapiens GN=EPB42 PE=1 SV=3 - [EPB42_HUMAN]	12,01	7
Q96PL5	Erythroid membrane-associated protein OS=Homo sapiens GN=ERMAP PE=1 SV=1 - [ERMAP_HUMAN]	1,68	1
P30042	ES1 protein homolog, mitochondrial OS=Homo sapiens GN=C21orf33 PE=1 SV=3 - [ES1_HUMAN]	7,46	2
Q8NBQ5	Estradiol 17-beta-dehydrogenase 11 OS=Homo sapiens GN=HSD17B11 PE=1 SV=3 - [DHB11_HUMAN]	11,33	3
Q53GQ0	Estradiol 17-beta-dehydrogenase 12 OS=Homo sapiens GN=HSD17B12 PE=1 SV=2 - [DHB12_HUMAN]	8,65	2
P60842	Eukaryotic initiation factor 4A-I OS=Homo sapiens GN=EIF4A1 PE=1 SV=1 - [IF4A1_HUMAN]	2,46	1
Q17RC7	Exocyst complex component 3-like protein 4 OS=Homo sapiens GN=EXOC3L4 PE=2 SV=2 - [EX3L4_HUMAN]	4,16	2
Q9BSJ8	Extended synaptotagmin-1 OS=Homo sapiens GN=ESYT1 PE=1 SV=1 - [ESYT1_HUMAN]	14,13	7
A0FGR8	Extended synaptotagmin-2 OS=Homo sapiens GN=ESYT2 PE=1 SV=1 - [ESYT2_HUMAN]	14,01	3
P15311	Ezrin OS=Homo sapiens GN=EZR PE=1 SV=4 - [EZRI_HUMAN]	20,31	4
P52907	F-actin-capping protein subunit alpha-1 OS=Homo sapiens GN=CAPZA1 PE=1 SV=3 - [CAZA1_HUMAN]	40,91	2
P47755	F-actin-capping protein subunit alpha-2 OS=Homo sapiens GN=CAPZA2 PE=1 SV=3 - [CAZA2_HUMAN]	22,73	3
P47756	F-actin-capping protein subunit beta OS=Homo sapiens GN=CAPZB PE=1 SV=4 - [CAPZB_HUMAN]	39,35	1
Q7L8L6	FAST kinase domain-containing protein 5 OS=Homo sapiens GN=FASTKD5 PE=1 SV=1 - [FAKD5_HUMAN]	1,96	1
P49327	Fatty acid synthase OS=Homo sapiens GN=FASN PE=1 SV=3 - [FAS_HUMAN]	0,96	2
Q6ZVX7	F-box only protein 50 OS=Homo sapiens GN=NCCRP1 PE=1 SV=1 - [FBX50_HUMAN]	3,64	1
Q86UX7	Fermitin family homolog 3 OS=Homo sapiens GN=FERMT3 PE=1 SV=1 - [URP2_HUMAN]	38,98	13
Q9UGM5	Fetuin-B OS=Homo sapiens GN=FETUB PE=1 SV=2 - [FETUB_HUMAN]	2,09	1
Q9Y613	FH1/FH2 domain-containing protein 1 OS=Homo sapiens GN=FHOD1 PE=1 SV=3 - [FHOD1_HUMAN]	0,86	1
P02671	Fibrinogen alpha chain OS=Homo sapiens GN=FGA PE=1 SV=2 - [FIBA_HUMAN]	28,52	15
P02675	Fibrinogen beta chain OS=Homo sapiens GN=FGB PE=1 SV=2 - [FIBB_HUMAN]	51,93	8
P02679	Fibrinogen gamma chain OS=Homo sapiens GN=FGG PE=1 SV=3 - [FIBG_HUMAN]	50,99	10
Q86WI1	Fibrocystin-L OS=Homo sapiens GN=PKHD1L1 PE=2 SV=2 - [PKHL1_HUMAN]	5,66	10
P02751	Fibronectin OS=Homo sapiens GN=FN1 PE=1 SV=4 - [FINC_HUMAN]	25,98	21
O75636	Ficolin-3 OS=Homo sapiens GN=FCN3 PE=1 SV=2 - [FCN3_HUMAN]	32,78	4
P20930	Filaggrin OS=Homo sapiens GN=FLG PE=1 SV=3 - [FILA_HUMAN]	3,03	2
Q5D862	Filaggrin-2 OS=Homo sapiens GN=FLG2 PE=1 SV=1 - [FILA2_HUMAN]	1,46	3
P21333	Filamin-A OS=Homo sapiens GN=FLNA PE=1 SV=4 - [FLNA_HUMAN]	51,76	75
Q5T1M5	FK506-binding protein 15 OS=Homo sapiens GN=FKBP15 PE=1 SV=2 - [FKB15_HUMAN]	1,15	1
O75955	Flotillin-1 OS=Homo sapiens GN=FLOT1 PE=1 SV=3 - [FLOT1_HUMAN]	34,19	11

Q14254	Flotillin-2 OS=Homo sapiens GN=FLOT2 PE=1 SV=2 - [FLOT2_HUMAN]	25,70	8
Q13642	Four and a half LIM domains protein 1 OS=Homo sapiens GN=FHL1 PE=1 SV=4 - [FHL1_HUMAN]	3,72	1
P04075	Fructose-bisphosphate aldolase A OS=Homo sapiens GN=ALDOA PE=1 SV=2 - [ALDOA_HUMAN]	21,15	4
P09972	Fructose-bisphosphate aldolase C OS=Homo sapiens GN=ALDOC PE=1 SV=2 - [ALDOC_HUMAN]	7,14	2
P07954	Fumarate hydratase, mitochondrial OS=Homo sapiens GN=FH PE=1 SV=3 - [FUMH_HUMAN]	10,00	3
P16930	Fumarylacetoacetase OS=Homo sapiens GN=FAH PE=1 SV=2 - [FAAA_HUMAN]	2,15	1
O15117	FYN-binding protein OS=Homo sapiens GN=FYB PE=1 SV=2 - [FYB_HUMAN]	9,71	6
Q08380	Galectin-3-binding protein OS=Homo sapiens GN=LGALS3BP PE=1 SV=1 - [LG3BP_HUMAN]	26,50	10
P47929	Galectin-7 OS=Homo sapiens GN=LGALS7 PE=1 SV=2 - [LEG7_HUMAN]	8,09	1
Q3ZCW2	Galectin-related protein OS=Homo sapiens GN=LGALSL PE=1 SV=2 - [LEGL_HUMAN]	6,40	1
P09104	Gamma-enolase OS=Homo sapiens GN=ENO2 PE=1 SV=3 - [ENOG_HUMAN]	9,45	2
O75223	Gamma-glutamylcyclotransferase OS=Homo sapiens GN=GGCT PE=1 SV=1 - [GGCT_HUMAN]	17,55	2
P17900	Ganglioside GM2 activator OS=Homo sapiens GN=GM2A PE=1 SV=4 - [SAP3_HUMAN]	8,81	2
Q96QA5	Gasdermin-A OS=Homo sapiens GN=GSDMA PE=2 SV=4 - [GSDMA_HUMAN]	3,82	2
P06396	Gelsolin OS=Homo sapiens GN=GSN PE=1 SV=1 - [GELS_HUMAN]	26,34	11
P11413	Glucose-6-phosphate 1-dehydrogenase OS=Homo sapiens GN=G6PD PE=1 SV=4 - [G6PD_HUMAN]	5,05	2
P06744	Glucose-6-phosphate isomerase OS=Homo sapiens GN=GPI PE=1 SV=4 - [G6PI_HUMAN]	5,20	3
P14314	Glucosidase 2 subunit beta OS=Homo sapiens GN=PRKCSH PE=1 SV=2 - [GLU2B_HUMAN]	10,42	3
P00367	Glutamate dehydrogenase 1, mitochondrial OS=Homo sapiens GN=GLUD1 PE=1 SV=2 - [DHE3_HUMAN]	18,46	3
O94925	Glutaminase kidney isoform, mitochondrial OS=Homo sapiens GN=GLS PE=1 SV=1 - [GLSK_HUMAN]	3,44	2
P00390	Glutathione reductase, mitochondrial OS=Homo sapiens GN=GSR PE=1 SV=2 - [GSHR_HUMAN]	4,41	1
Q9Y2Q3	Glutathione S-transferase kappa 1 OS=Homo sapiens GN=GSTK1 PE=1 SV=3 - [GSTK1_HUMAN]	23,45	3
P78417	Glutathione S-transferase omega-1 OS=Homo sapiens GN=GSTO1 PE=1 SV=2 - [GSTO1_HUMAN]	11,20	1
P04406	Glyceraldehyde-3-phosphate dehydrogenase OS=Homo sapiens GN=GAPDH PE=1 SV=3 - [G3P_HUMAN]	23,58	3
P43304	Glycerol-3-phosphate dehydrogenase, mitochondrial OS=Homo sapiens GN=GPD2 PE=1 SV=3 - [GPDM_HUMAN]	17,74	10
P50440	Glycine amidinotransferase, mitochondrial OS=Homo sapiens GN=GATM PE=1 SV=1 - [GATM_HUMAN]	2,84	1
P02724	Glycophorin-A OS=Homo sapiens GN=GYPA PE=1 SV=2 - [GLPA_HUMAN]	20,00	1
P04921	Glycophorin-C OS=Homo sapiens GN=GYPC PE=1 SV=1 - [GLPC_HUMAN]	20,31	1
Q9UBQ7	Glyoxylate reductase/hydroxypyruvate reductase OS=Homo sapiens GN=GRHPR PE=1 SV=1 - [GRHPR_HUMAN]	8,23	1
P36959	GMP reductase 1 OS=Homo sapiens GN=GMPR PE=1 SV=1 - [GMPR1_HUMAN]	3,48	1
Q9H4G4	Golgi-associated plant pathogenesis-related protein 1 OS=Homo sapiens GN=GLIPR2 PE=1 SV=3 - [GAPR1_HUMAN]	8,44	1
Q08379	Golgin subfamily A member 2 OS=Homo sapiens GN=GOLGA2 PE=1 SV=3 - [GOGA2_HUMAN]	2,00	2
Q8TBA6	Golgin subfamily A member 5 OS=Homo sapiens GN=GOLGA5 PE=1 SV=3 - [GOGA5_HUMAN]	1,78	1
Q4V328	GRIP1-associated protein 1 OS=Homo sapiens GN=GRIPAP1 PE=1 SV=1 - [GRAP1_HUMAN]	1,19	1
P62993	Growth factor receptor-bound protein 2 OS=Homo sapiens GN=GRB2 PE=1 SV=1 - [GRB2_HUMAN]	23,50	2
Q9H3K2	Growth hormone-inducible transmembrane protein OS=Homo sapiens GN=GHITM PE=1 SV=2 - [GHITM_HUMAN]	12,46	1
Q9UIJ7	GTP:AMP phosphotransferase AK3, mitochondrial OS=Homo sapiens GN=AK3 PE=1 SV=4 - [KAD3_HUMAN]	17,62	4
P01111	GTPase NRas OS=Homo sapiens GN=NRAS PE=1 SV=1 - [RASN_HUMAN]	10,58	1
P63096	Guanine nucleotide-binding protein G(i) subunit alpha-1 OS=Homo sapiens GN=GNAI1 PE=1 SV=2 - [GNAI1_HUMAN]	18,93	3
P04899	Guanine nucleotide-binding protein G(i) subunit alpha-2 OS=Homo sapiens GN=GNAI2 PE=1 SV=3 - [GNAI2_HUMAN]	38,03	8

P61952	Guanine nucleotide-binding protein G(I)/G(S)/G(O) subunit gamma-11 OS=Homo sapiens GN=GNG11 PE=1 SV=1 - [GBG11_HUMA	19,18	1
P63218	Guanine nucleotide-binding protein G(I)/G(S)/G(O) subunit gamma-5 OS=Homo sapiens GN=GNG5 PE=1 SV=3 - [GBG5_HUMAN]	23,53	2
O60262	Guanine nucleotide-binding protein G(I)/G(S)/G(O) subunit gamma-7 OS=Homo sapiens GN=GNG7 PE=1 SV=1 - [GBG7_HUMAN]	23,53	1
P62873	Guanine nucleotide-binding protein G(I)/G(S)/G(T) subunit beta-1 OS=Homo sapiens GN=GNB1 PE=1 SV=3 - [GBB1_HUMAN]	23,82	5
P62879	Guanine nucleotide-binding protein G(I)/G(S)/G(T) subunit beta-2 OS=Homo sapiens GN=GNB2 PE=1 SV=3 - [GBB2_HUMAN]	25,59	3
P08754	Guanine nucleotide-binding protein G(k) subunit alpha OS=Homo sapiens GN=GNAI3 PE=1 SV=3 - [GNAI3_HUMAN]	16,67	3
P50148	Guanine nucleotide-binding protein G(q) subunit alpha OS=Homo sapiens GN=GNAQ PE=1 SV=4 - [GNAQ_HUMAN]	33,43	6
P63092	Guanine nucleotide-binding protein G(s) subunit alpha isoforms short OS=Homo sapiens GN=GNAS PE=1 SV=1 - [GNAS2_HUMAN]	12,44	3
P19086	Guanine nucleotide-binding protein G(z) subunit alpha OS=Homo sapiens GN=GNAZ PE=2 SV=3 - [GNAZ_HUMAN]	24,79	6
Q14344	Guanine nucleotide-binding protein subunit alpha-13 OS=Homo sapiens GN=GNA13 PE=1 SV=2 - [GNA13_HUMAN]	31,30	5
Q9HAV0	Guanine nucleotide-binding protein subunit beta-4 OS=Homo sapiens GN=GNB4 PE=1 SV=3 - [GBB4_HUMAN]	16,76	4
P51790	H(+)/Cl(-) exchange transporter 3 OS=Homo sapiens GN=CLCN3 PE=1 SV=2 - [CLCN3_HUMAN]	3,30	1
P00738	Haptoglobin OS=Homo sapiens GN=HP PE=1 SV=1 - [HPT_HUMAN]	42,86	14
P00739	Haptoglobin-related protein OS=Homo sapiens GN=HPR PE=1 SV=2 - [HPTR_HUMAN]	40,23	10
P08107	Heat shock 70 kDa protein 1A/1B OS=Homo sapiens GN=HSPA1A PE=1 SV=5 - [HSP71_HUMAN]	26,52	10
P11142	Heat shock cognate 71 kDa protein OS=Homo sapiens GN=HSPA8 PE=1 SV=1 - [HSP7C_HUMAN]	32,82	14
Q12931	Heat shock protein 75 kDa, mitochondrial OS=Homo sapiens GN=TRAP1 PE=1 SV=3 - [TRAP1_HUMAN]	4,97	2
P04792	Heat shock protein beta-1 OS=Homo sapiens GN=HSPB1 PE=1 SV=2 - [HSPB1_HUMAN]	44,88	4
P07900	Heat shock protein HSP 90-alpha OS=Homo sapiens GN=HSP90AA1 PE=1 SV=5 - [HS90A_HUMAN]	17,62	5
P08238	Heat shock protein HSP 90-beta OS=Homo sapiens GN=HSP90AB1 PE=1 SV=4 - [HS90B_HUMAN]	17,27	3
P69905	Hemoglobin subunit alpha OS=Homo sapiens GN=HBA1 PE=1 SV=2 - [HBA_HUMAN]	39,44	4
P68871	Hemoglobin subunit beta OS=Homo sapiens GN=HBB PE=1 SV=2 - [HBB_HUMAN]	70,07	5
P02042	Hemoglobin subunit delta OS=Homo sapiens GN=HBD PE=1 SV=2 - [HBD_HUMAN]	50,34	3
P02790	Hemopexin OS=Homo sapiens GN=HPX PE=1 SV=2 - [HEMO_HUMAN]	8,66	3
Q9Y251	Heparanase OS=Homo sapiens GN=HPSE PE=1 SV=2 - [HPSE_HUMAN]	16,02	3
P05546	Heparin cofactor 2 OS=Homo sapiens GN=SERPIND1 PE=1 SV=3 - [HEP2_HUMAN]	7,01	2
P61978	Heterogeneous nuclear ribonucleoprotein K OS=Homo sapiens GN=HNRNPK PE=1 SV=1 - [HNRPK_HUMAN]	3,67	1
P19367	Hexokinase-1 OS=Homo sapiens GN=HK1 PE=1 SV=3 - [HXK1_HUMAN]	3,27	2
P30273	High affinity immunoglobulin epsilon receptor subunit gamma OS=Homo sapiens GN=FCER1G PE=1 SV=1 - [FCERG_HUMAN]	23,26	3
P42357	Histidine ammonia-lyase OS=Homo sapiens GN=HAL PE=1 SV=1 - [HUTH_HUMAN]	4,26	3
Q9BX68	Histidine triad nucleotide-binding protein 2, mitochondrial OS=Homo sapiens GN=HINT2 PE=1 SV=1 - [HINT2_HUMAN]	9,82	1
P0C0S5	Histone H2A.Z OS=Homo sapiens GN=H2AFZ PE=1 SV=2 - [H2AZ_HUMAN]	7,03	1
O60814	Histone H2B type 1-K OS=Homo sapiens GN=HIST1H2BK PE=1 SV=3 - [H2B1K_HUMAN]	21,43	3
P68431	Histone H3.1 OS=Homo sapiens GN=HIST1H3A PE=1 SV=2 - [H31_HUMAN]	10,29	2
P62805	Histone H4 OS=Homo sapiens GN=HIST1H4A PE=1 SV=2 - [H4_HUMAN]	21,36	2
P30443	HLA class I histocompatibility antigen, A-1 alpha chain OS=Homo sapiens GN=HLA-A PE=1 SV=1 - [1A01_HUMAN]	32,33	5
P05534	HLA class I histocompatibility antigen, A-24 alpha chain OS=Homo sapiens GN=HLA-A PE=1 SV=2 - [1A24_HUMAN]	38,90	6
P30456	HLA class I histocompatibility antigen, A-43 alpha chain OS=Homo sapiens GN=HLA-A PE=1 SV=1 - [1A43_HUMAN]	36,44	7
P10316	HLA class I histocompatibility antigen, A-69 alpha chain OS=Homo sapiens GN=HLA-A PE=1 SV=2 - [1A69_HUMAN]	36,44	6
P13747	HLA class I histocompatibility antigen, alpha chain E OS=Homo sapiens GN=HLA-E PE=1 SV=3 - [HLAE_HUMAN]	16,20	3

P30481	HLA class I histocompatibility antigen, B-44 alpha chain OS=Homo sapiens GN=HLA-B PE=1 SV=1 - [1B44_HUMAN]	33,70	4
P30483	HLA class I histocompatibility antigen, B-45 alpha chain OS=Homo sapiens GN=HLA-B PE=1 SV=1 - [1B45_HUMAN]	33,70	5
P01889	HLA class I histocompatibility antigen, B-7 alpha chain OS=Homo sapiens GN=HLA-B PE=1 SV=3 - [1B07_HUMAN]	37,85	5
P30498	HLA class I histocompatibility antigen, B-78 alpha chain OS=Homo sapiens GN=HLA-B PE=1 SV=1 - [1B78_HUMAN]	28,45	5
Q16836	Hydroxyacyl-coenzyme A dehydrogenase, mitochondrial OS=Homo sapiens GN=HADH PE=1 SV=3 - [HCDH_HUMAN]	12,74	3
P35914	Hydroxymethylglutaryl-CoA lyase, mitochondrial OS=Homo sapiens GN=HMGCL PE=1 SV=2 - [HMGCL_HUMAN]	6,46	2
Q9Y4L1	Hypoxia up-regulated protein 1 OS=Homo sapiens GN=HYOU1 PE=1 SV=1 - [HYOU1_HUMAN]	10,31	5
P01876	Ig alpha-1 chain C region OS=Homo sapiens GN=IGHA1 PE=1 SV=2 - [IGHA1_HUMAN]	39,38	8
P01877	Ig alpha-2 chain C region OS=Homo sapiens GN=IGHA2 PE=1 SV=3 - [IGHA2_HUMAN]	34,71	6
P01880	Ig delta chain C region OS=Homo sapiens GN=IGHD PE=1 SV=2 - [IGHD_HUMAN]	11,46	2
P01857	Ig gamma-1 chain C region OS=Homo sapiens GN=IGHG1 PE=1 SV=1 - [IGHG1_HUMAN]	42,12	8
P01859	Ig gamma-2 chain C region OS=Homo sapiens GN=IGHG2 PE=1 SV=2 - [IGHG2_HUMAN]	30,67	8
P01860	Ig gamma-3 chain C region OS=Homo sapiens GN=IGHG3 PE=1 SV=2 - [IGHG3_HUMAN]	37,67	8
P01861	Ig gamma-4 chain C region OS=Homo sapiens GN=IGHG4 PE=1 SV=1 - [IGHG4_HUMAN]	34,25	6
P23083	Ig heavy chain V-I region 2 OS=Homo sapiens GN=IGHV1-2 PE=1 SV=2 - [HV103_HUMAN]	22,22	2
P01602	Ig heavy chain V-I region 5 (Fragment) OS=Homo sapiens GN=IGKV1-5 PE=4 SV=2 - [KV110_HUMAN]	13,68	1
P01764	Ig heavy chain V-III region 23 OS=Homo sapiens GN=IGHV3-23 PE=1 SV=2 - [HV303_HUMAN]	47,01	2
P01834	Ig kappa chain C region OS=Homo sapiens GN=IGKC PE=1 SV=1 - [IGKC_HUMAN]	85,85	4
P06312	Ig kappa chain V-IV region (Fragment) OS=Homo sapiens GN=IGKV4-1 PE=4 SV=1 - [KV401_HUMAN]	17,36	1
P01701	Ig lambda chain V-I region 51 OS=Homo sapiens GN=IGLV1-51 PE=1 SV=2 - [LV151_HUMAN]	13,68	1
P0CG05	Ig lambda-2 chain C regions OS=Homo sapiens GN=IGLC2 PE=1 SV=1 - [LAC2_HUMAN]	62,26	5
P01871	Ig mu chain C region OS=Homo sapiens GN=IGHM PE=1 SV=3 - [IGHM_HUMAN]	49,12	16
P04220	Ig mu heavy chain disease protein OS=Homo sapiens PE=1 SV=1 - [MUCB_HUMAN]	40,41	9
Q9Y6R7	IgGFc-binding protein OS=Homo sapiens GN=FCGBP PE=1 SV=3 - [FCGBP_HUMAN]	9,20	7
A0A0C4DH31	Immunoglobulin heavy variable 1-18 OS=Homo sapiens GN=IGHV1-18 PE=3 SV=1 - [HV118_HUMAN]	23,93	2
A0A0C4DH29	Immunoglobulin heavy variable 1-3 OS=Homo sapiens GN=IGHV1-3 PE=3 SV=1 - [HV103_HUMAN]	22,22	2
P01743	Immunoglobulin heavy variable 1-46 OS=Homo sapiens GN=IGHV1-46 PE=1 SV=2 - [HV146_HUMAN]	22,22	1
P01766	Immunoglobulin heavy variable 3-13 OS=Homo sapiens GN=IGHV3-13 PE=1 SV=2 - [HV313_HUMAN]	25,00	1
A0A0B4J1V0	Immunoglobulin heavy variable 3-15 OS=Homo sapiens GN=IGHV3-15 PE=3 SV=1 - [HV315_HUMAN]	36,97	3
P01772	Immunoglobulin heavy variable 3-33 OS=Homo sapiens GN=IGHV3-33 PE=1 SV=2 - [HV333_HUMAN]	34,19	2
A0A0A0MS15	Immunoglobulin heavy variable 3-49 OS=Homo sapiens GN=IGHV3-49 PE=1 SV=1 - [HV349_HUMAN]	46,22	1
P01780	Immunoglobulin heavy variable 3-7 OS=Homo sapiens GN=IGHV3-7 PE=1 SV=2 - [HV307_HUMAN]	43,59	3
A0A0B4J1Y9	Immunoglobulin heavy variable 3-72 OS=Homo sapiens GN=IGHV3-72 PE=3 SV=1 - [HV372_HUMAN]	44,54	4
A0A0B4J1V6	Immunoglobulin heavy variable 3-73 OS=Homo sapiens GN=IGHV3-73 PE=3 SV=1 - [HV373_HUMAN]	44,54	2
A0A0B4J1X5	Immunoglobulin heavy variable 3-74 OS=Homo sapiens GN=IGHV3-74 PE=3 SV=1 - [HV374_HUMAN]	43,59	3
P01782	Immunoglobulin heavy variable 3-9 OS=Homo sapiens GN=IGHV3-9 PE=1 SV=2 - [HV309_HUMAN]	24,58	2
A0A0C4DH34	Immunoglobulin heavy variable 4-28 OS=Homo sapiens GN=IGHV4-28 PE=3 SV=1 - [HV428_HUMAN]	7,69	1
P01825	Immunoglobulin heavy variable 4-59 OS=Homo sapiens GN=IGHV4-59 PE=1 SV=2 - [HV459_HUMAN]	26,72	2
A0A0C4DH38	Immunoglobulin heavy variable 5-51 OS=Homo sapiens GN=IGHV5-51 PE=3 SV=1 - [HV551_HUMAN]	47,01	3
A0A0B4J1U7	Immunoglobulin heavy variable 6-1 OS=Homo sapiens GN=IGHV6-1 PE=3 SV=1 - [HV601_HUMAN]	21,49	1

P01591	Immunoglobulin J chain OS=Homo sapiens GN=IGJ PE=1 SV=4 - [IGJ_HUMAN]	48,43	5
P04430	Immunoglobulin kappa variable 1-16 OS=Homo sapiens GN=IGKV1-16 PE=1 SV=2 - [KV116_HUMAN]	13,68	1
P01599	Immunoglobulin kappa variable 1-17 OS=Homo sapiens GN=IGKV1-17 PE=1 SV=2 - [KV107_HUMAN]	23,93	1
P01594	Immunoglobulin kappa variable 1-33 OS=Homo sapiens GN=IGKV1-33 PE=1 SV=2 - [KV133_HUMAN]	13,68	1
A0A0C4DH72	Immunoglobulin kappa variable 1-6 OS=Homo sapiens GN=IGKV1-6 PE=3 SV=1 - [KV106_HUMAN]	23,93	1
A0A0C4DH67	Immunoglobulin kappa variable 1-8 OS=Homo sapiens GN=IGKV1-8 PE=3 SV=1 - [KV108_HUMAN]	26,96	2
A0A0C4DH68	Immunoglobulin kappa variable 2-24 OS=Homo sapiens GN=IGKV2-24 PE=3 SV=1 - [KV224_HUMAN]	10,83	1
P06310	Immunoglobulin kappa variable 2-30 OS=Homo sapiens GN=IGKV2-30 PE=3 SV=2 - [KV230_HUMAN]	19,17	1
A0A075B6S2	Immunoglobulin kappa variable 2D-29 OS=Homo sapiens GN=IGKV2D-29 PE=3 SV=1 - [KVD29_HUMAN]	22,50	1
A0A075B6S6	Immunoglobulin kappa variable 2D-30 OS=Homo sapiens GN=IGKV2D-30 PE=3 SV=1 - [KVD30_HUMAN]	22,50	1
P01619	Immunoglobulin kappa variable 3-20 OS=Homo sapiens GN=IGKV3-20 PE=1 SV=2 - [KV320_HUMAN]	21,55	1
A0A0C4DH25	Immunoglobulin kappa variable 3-20 OS=Homo sapiens GN=IGKV3D-20 PE=3 SV=1 - [KVD20_HUMAN]	21,55	1
A0A0A0MRZ8	Immunoglobulin kappa variable 3D-11 OS=Homo sapiens GN=IGKV3D-11 PE=3 SV=6 - [KVD11_HUMAN]	26,09	1
P01700	Immunoglobulin lambda variable 1-47 OS=Homo sapiens GN=IGLV1-47 PE=1 SV=2 - [LV147_HUMAN]	24,79	1
P01706	Immunoglobulin lambda variable 2-11 OS=Homo sapiens GN=IGLV2-11 PE=1 SV=2 - [LV211_HUMAN]	13,45	2
P01705	Immunoglobulin lambda variable 2-23 OS=Homo sapiens GN=IGLV2-23 PE=1 SV=2 - [LV223_HUMAN]	7,08	1
P01709	Immunoglobulin lambda variable 2-8 OS=Homo sapiens GN=IGLV2-8 PE=1 SV=2 - [LV208_HUMAN]	13,56	2
A0A075B6K4	Immunoglobulin lambda variable 3-10 OS=Homo sapiens GN=IGLV3-10 PE=3 SV=2 - [LV310_HUMAN]	27,83	3
P01714	Immunoglobulin lambda variable 3-19 OS=Homo sapiens GN=IGLV3-19 PE=1 SV=2 - [LV319_HUMAN]	16,96	2
P80748	Immunoglobulin lambda variable 3-21 OS=Homo sapiens GN=IGLV3-21 PE=1 SV=2 - [LV321_HUMAN]	23,08	1
P01717	Immunoglobulin lambda variable 3-25 OS=Homo sapiens GN=IGLV3-25 PE=1 SV=2 - [LV325_HUMAN]	18,75	2
A0A075B6I1	Immunoglobulin lambda variable 4-60 OS=Homo sapiens GN=IGLV4-60 PE=3 SV=1 - [LV460_HUMAN]	15,83	2
A0A075B6H9	Immunoglobulin lambda variable 4-69 OS=Homo sapiens GN=IGLV4-69 PE=1 SV=1 - [LV469_HUMAN]	21,85	1
A0A0G2JS06	Immunoglobulin lambda variable 5-39 OS=Homo sapiens GN=IGLV5-39 PE=3 SV=1 - [LV539_HUMAN]	14,63	2
P04211	Immunoglobulin lambda variable 7-43 OS=Homo sapiens GN=IGLV7-43 PE=3 SV=2 - [LV743_HUMAN]	21,37	1
A0A075B6I9	Immunoglobulin lambda variable 7-46 OS=Homo sapiens GN=IGLV7-46 PE=3 SV=4 - [LV746_HUMAN]	21,37	2
A0A075B6I0	Immunoglobulin lambda variable 8-61 OS=Homo sapiens GN=IGLV8-61 PE=3 SV=7 - [LV861_HUMAN]	14,75	2
A0A0B4J1Y8	Immunoglobulin lambda variable 9-49 OS=Homo sapiens GN=IGLV9-49 PE=1 SV=1 - [LV949_HUMAN]	8,13	1
P15814	Immunoglobulin lambda-like polypeptide 1 OS=Homo sapiens GN=IGLL1 PE=1 SV=1 - [IGLL1_HUMAN]	7,04	1
B9A064	Immunoglobulin lambda-like polypeptide 5 OS=Homo sapiens GN=IGLL5 PE=2 SV=2 - [IGLL5_HUMAN]	26,64	4
Q14974	Importin subunit beta-1 OS=Homo sapiens GN=KPNB1 PE=1 SV=2 - [IMB1_HUMAN]	1,37	1
Q3SXM5	Inactive hydroxysteroid dehydrogenase-like protein 1 OS=Homo sapiens GN=HSDL1 PE=1 SV=3 - [HSDL1_HUMAN]	7,58	1
Q70UQ0	Inhibitor of nuclear factor kappa-B kinase-interacting protein OS=Homo sapiens GN=IKBIP PE=1 SV=1 - [IKIP_HUMAN]	3,43	1
Q9H2U2	Inorganic pyrophosphatase 2, mitochondrial OS=Homo sapiens GN=PPA2 PE=1 SV=2 - [IPYR2_HUMAN]	9,88	2
Q14643	Inositol 1,4,5-trisphosphate receptor type 1 OS=Homo sapiens GN=ITPR1 PE=1 SV=3 - [ITPR1_HUMAN]	6,74	14
Q14571	Inositol 1,4,5-trisphosphate receptor type 2 OS=Homo sapiens GN=ITPR2 PE=1 SV=2 - [ITPR2_HUMAN]	4,85	8
P08069	Insulin-like growth factor 1 receptor OS=Homo sapiens GN=IGF1R PE=1 SV=1 - [IGF1R_HUMAN]	0,66	1
P17301	Integrin alpha-2 OS=Homo sapiens GN=ITGA2 PE=1 SV=1 - [ITA2_HUMAN]	24,81	14
P08648	Integrin alpha-5 OS=Homo sapiens GN=ITGA5 PE=1 SV=2 - [ITA5_HUMAN]	8,01	4
P23229	Integrin alpha-6 OS=Homo sapiens GN=ITGA6 PE=1 SV=5 - [ITA6_HUMAN]	23,10	14

P08514	Integrin alpha-IIb OS=Homo sapiens GN=ITGA2B PE=1 SV=3 - [ITA2B_HUMAN]	33,78	21
P06756	Integrin alpha-V OS=Homo sapiens GN=ITGAV PE=1 SV=2 - [ITAV_HUMAN]	10,02	1
P05556	Integrin beta-1 OS=Homo sapiens GN=ITGB1 PE=1 SV=2 - [ITB1_HUMAN]	18,05	10
P05107	Integrin beta-2 OS=Homo sapiens GN=ITGB2 PE=1 SV=2 - [ITB2_HUMAN]	4,29	2
P05106	Integrin beta-3 OS=Homo sapiens GN=ITGB3 PE=1 SV=2 - [ITB3_HUMAN]	39,85	22
Q13418	Integrin-linked protein kinase OS=Homo sapiens GN=ILK PE=1 SV=2 - [ILK_HUMAN]	29,87	8
P19827	Inter-alpha-trypsin inhibitor heavy chain H1 OS=Homo sapiens GN=ITIH1 PE=1 SV=3 - [ITIH1_HUMAN]	8,67	6
P19823	Inter-alpha-trypsin inhibitor heavy chain H2 OS=Homo sapiens GN=ITIH2 PE=1 SV=2 - [ITIH2_HUMAN]	13,00	10
Q06033	Inter-alpha-trypsin inhibitor heavy chain H3 OS=Homo sapiens GN=ITIH3 PE=1 SV=2 - [ITIH3_HUMAN]	1,12	1
Q14624	Inter-alpha-trypsin inhibitor heavy chain H4 OS=Homo sapiens GN=ITIH4 PE=1 SV=4 - [ITIH4_HUMAN]	12,58	9
P13598	Intercellular adhesion molecule 2 OS=Homo sapiens GN=ICAM2 PE=1 SV=2 - [ICAM2_HUMAN]	11,64	3
P32942	Intercellular adhesion molecule 3 OS=Homo sapiens GN=ICAM3 PE=1 SV=2 - [ICAM3_HUMAN]	3,11	2
P13164	Interferon-induced transmembrane protein 1 OS=Homo sapiens GN=IFITM1 PE=1 SV=3 - [IFM1_HUMAN]	12,80	1
Q27J81	Inverted formin-2 OS=Homo sapiens GN=INF2 PE=1 SV=2 - [INF2_HUMAN]	2,64	3
P50213	Isocitrate dehydrogenase [NAD] subunit alpha, mitochondrial OS=Homo sapiens GN=IDH3A PE=1 SV=1 - [IDH3A_HUMAN]	3,01	1
O43837	Isocitrate dehydrogenase [NAD] subunit beta, mitochondrial OS=Homo sapiens GN=IDH3B PE=1 SV=2 - [IDH3B_HUMAN]	4,16	1
P48735	Isocitrate dehydrogenase [NADP], mitochondrial OS=Homo sapiens GN=IDH2 PE=1 SV=2 - [IDHP_HUMAN]	32,30	10
P26440	Isovaleryl-CoA dehydrogenase, mitochondrial OS=Homo sapiens GN=IVD PE=1 SV=1 - [IVD_HUMAN]	7,09	3
P14923	Junction plakoglobin OS=Homo sapiens GN=JUP PE=1 SV=3 - [PLAK_HUMAN]	16,38	6
Q9Y624	Junctional adhesion molecule A OS=Homo sapiens GN=F11R PE=1 SV=1 - [JAM1_HUMAN]	50,84	9
Q9BX67	Junctional adhesion molecule C OS=Homo sapiens GN=JAM3 PE=1 SV=1 - [JAM3_HUMAN]	18,71	4
O60229	Kalirin OS=Homo sapiens GN=KALRN PE=1 SV=2 - [KALRN_HUMAN]	2,48	3
P49862	Kallikrein-7 OS=Homo sapiens GN=KLK7 PE=1 SV=1 - [KLK7_HUMAN]	3,56	1
P29622	Kallistatin OS=Homo sapiens GN=SERPINA4 PE=1 SV=3 - [KAIN_HUMAN]	7,03	1
Q14525	Keratin, type I cuticular Ha3-II OS=Homo sapiens GN=KRT33B PE=2 SV=3 - [KT33B_HUMAN]	3,96	1
P13645	Keratin, type I cytoskeletal 10 OS=Homo sapiens GN=KRT10 PE=1 SV=6 - [K1C10_HUMAN]	48,12	23
P13646	Keratin, type I cytoskeletal 13 OS=Homo sapiens GN=KRT13 PE=1 SV=4 - [K1C13_HUMAN]	15,07	4
P02533	Keratin, type I cytoskeletal 14 OS=Homo sapiens GN=KRT14 PE=1 SV=4 - [K1C14_HUMAN]	58,90	19
P08779	Keratin, type I cytoskeletal 16 OS=Homo sapiens GN=KRT16 PE=1 SV=4 - [K1C16_HUMAN]	52,01	17
Q04695	Keratin, type I cytoskeletal 17 OS=Homo sapiens GN=KRT17 PE=1 SV=2 - [K1C17_HUMAN]	43,52	15
Q7Z3Y8	Keratin, type I cytoskeletal 27 OS=Homo sapiens GN=KRT27 PE=1 SV=2 - [K1C27_HUMAN]	6,54	3
P35527	Keratin, type I cytoskeletal 9 OS=Homo sapiens GN=KRT9 PE=1 SV=3 - [K1C9_HUMAN]	42,70	13
P04264	Keratin, type II cytoskeletal 1 OS=Homo sapiens GN=KRT1 PE=1 SV=6 - [K2C1_HUMAN]	57,61	28
Q7Z794	Keratin, type II cytoskeletal 1b OS=Homo sapiens GN=KRT77 PE=2 SV=3 - [K2C1B_HUMAN]	22,32	9
P35908	Keratin, type II cytoskeletal 2 epidermal OS=Homo sapiens GN=KRT2 PE=1 SV=2 - [K22E_HUMAN]	69,17	26
P13647	Keratin, type II cytoskeletal 5 OS=Homo sapiens GN=KRT5 PE=1 SV=3 - [K2C5_HUMAN]	44,41	20
P02538	Keratin, type II cytoskeletal 6A OS=Homo sapiens GN=KRT6A PE=1 SV=3 - [K2C6A_HUMAN]	44,33	16
P04259	Keratin, type II cytoskeletal 6B OS=Homo sapiens GN=KRT6B PE=1 SV=5 - [K2C6B_HUMAN]	44,15	15
Q7RTS7	Keratin, type II cytoskeletal 74 OS=Homo sapiens GN=KRT74 PE=1 SV=2 - [K2C74_HUMAN]	7,56	5
Q8N1N4	Keratin, type II cytoskeletal 78 OS=Homo sapiens GN=KRT78 PE=2 SV=2 - [K2C78_HUMAN]	12,50	5

Q5XKE5	Keratin, type II cytoskeletal 79 OS=Homo sapiens GN=KRT79 PE=1 SV=2 - [K2C79_HUMAN]	11,21	3
Q6KB66	Keratin, type II cytoskeletal 80 OS=Homo sapiens GN=KRT80 PE=1 SV=2 - [K2C80_HUMAN]	13,05	4
Q5T749	Keratinocyte proline-rich protein OS=Homo sapiens GN=KPRP PE=1 SV=1 - [KPRP_HUMAN]	7,08	2
O00139	Kinesin-like protein KIF2A OS=Homo sapiens GN=KIF2A PE=1 SV=3 - [KIF2A_HUMAN]	5,24	4
P01042	Kininogen-1 OS=Homo sapiens GN=KNG1 PE=1 SV=2 - [KNG1_HUMAN]	10,71	6
Q08431	Lactadherin OS=Homo sapiens GN=MFGE8 PE=1 SV=2 - [MFGM_HUMAN]	7,49	1
P02788	Lactotransferrin OS=Homo sapiens GN=LTF PE=1 SV=6 - [TRFL_HUMAN]	13,80	2
Q14739	Lamin-B receptor OS=Homo sapiens GN=LBR PE=1 SV=2 - [LBR_HUMAN]	1,30	1
P48449	Lanosterol synthase OS=Homo sapiens GN=LSS PE=1 SV=1 - [ERG7_HUMAN]	1,64	1
O75387	Large neutral amino acids transporter small subunit 3 OS=Homo sapiens GN=SLC43A1 PE=2 SV=1 - [LAT3_HUMAN]	1,61	1
Q14766	Latent-transforming growth factor beta-binding protein 1 OS=Homo sapiens GN=LTBP1 PE=1 SV=4 - [LTBP1_HUMAN]	5,75	8
Q14696	LDLR chaperone MESD OS=Homo sapiens GN=MESDC2 PE=1 SV=2 - [MESD_HUMAN]	5,98	1
O95202	LETM1 and EF-hand domain-containing protein 1, mitochondrial OS=Homo sapiens GN=LETM1 PE=1 SV=1 - [LETM1_HUMAN]	1,62	1
P02750	Leucine-rich alpha-2-glycoprotein OS=Homo sapiens GN=LRG1 PE=1 SV=2 - [A2GL_HUMAN]	9,80	4
P42704	Leucine-rich PPR motif-containing protein, mitochondrial OS=Homo sapiens GN=LRPPRC PE=1 SV=3 - [LPPRC_HUMAN]	3,23	4
Q32MZ4	Leucine-rich repeat flightless-interacting protein 1 OS=Homo sapiens GN=LRRFIP1 PE=1 SV=2 - [LRRF1_HUMAN]	1,73	1
Q9Y608	Leucine-rich repeat flightless-interacting protein 2 OS=Homo sapiens GN=LRRFIP2 PE=1 SV=1 - [LRRF2_HUMAN]	3,05	2
Q14392	Leucine-rich repeat-containing protein 32 OS=Homo sapiens GN=LRRC32 PE=1 SV=1 - [LRC32_HUMAN]	1,51	1
Q96AG4	Leucine-rich repeat-containing protein 59 OS=Homo sapiens GN=LRRC59 PE=1 SV=1 - [LRC59_HUMAN]	17,26	1
Q7L1W4	Leucine-rich repeat-containing protein 8D OS=Homo sapiens GN=LRRC8D PE=1 SV=1 - [LRC8D_HUMAN]	4,43	2
P30740	Leukocyte elastase inhibitor OS=Homo sapiens GN=SERPINB1 PE=1 SV=1 - [ILEU_HUMAN]	25,33	1
Q08722	Leukocyte surface antigen CD47 OS=Homo sapiens GN=CD47 PE=1 SV=1 - [CD47_HUMAN]	8,67	3
P16150	Leukosialin OS=Homo sapiens GN=SPN PE=1 SV=1 - [LEUK_HUMAN]	5,50	2
Q16873	Leukotriene C4 synthase OS=Homo sapiens GN=LTC4S PE=1 SV=1 - [LTC4S_HUMAN]	9,33	1
P48059	LIM and senescent cell antigen-like-containing domain protein 1 OS=Homo sapiens GN=LIMS1 PE=1 SV=4 - [LIMS1_HUMAN]	7,69	1
Q14847	LIM and SH3 domain protein 1 OS=Homo sapiens GN=LASP1 PE=1 SV=2 - [LASP1_HUMAN]	3,83	1
O43561	Linker for activation of T-cells family member 1 OS=Homo sapiens GN=LAT PE=1 SV=1 - [LAT_HUMAN]	6,11	1
Q9BU23	Lipase maturation factor 2 OS=Homo sapiens GN=LMF2 PE=1 SV=2 - [LMF2_HUMAN]	1,13	1
P18428	Lipopolysaccharide-binding protein OS=Homo sapiens GN=LBP PE=1 SV=3 - [LBP_HUMAN]	2,29	1
P50851	Lipopolysaccharide-responsive and beige-like anchor protein OS=Homo sapiens GN=LRBA PE=1 SV=4 - [LRBA_HUMAN]	1,71	2
P00338	L-lactate dehydrogenase A chain OS=Homo sapiens GN=LDHA PE=1 SV=2 - [LDHA_HUMAN]	20,78	7
P07195	L-lactate dehydrogenase B chain OS=Homo sapiens GN=LDHB PE=1 SV=2 - [LDHB_HUMAN]	18,56	4
P36776	Lon protease homolog, mitochondrial OS=Homo sapiens GN=LONP1 PE=1 SV=2 - [LONM_HUMAN]	1,46	1
Q6P1M0	Long-chain fatty acid transport protein 4 OS=Homo sapiens GN=SLC27A4 PE=1 SV=1 - [S27A4_HUMAN]	6,84	1
O95573	Long-chain-fatty-acid--CoA ligase 3 OS=Homo sapiens GN=ACSL3 PE=1 SV=3 - [ACSL3_HUMAN]	2,08	1
O60488	Long-chain-fatty-acid--CoA ligase 4 OS=Homo sapiens GN=ACSL4 PE=1 SV=2 - [ACSL4_HUMAN]	1,83	1
Q9ULC5	Long-chain-fatty-acid--CoA ligase 5 OS=Homo sapiens GN=ACSL5 PE=1 SV=1 - [ACSL5_HUMAN]	3,81	3
P23490	Loricrin OS=Homo sapiens GN=LOR PE=1 SV=2 - [LORI_HUMAN]	2,56	1
P12318	Low affinity immunoglobulin gamma Fc region receptor II-a OS=Homo sapiens GN=FCGR2A PE=1 SV=4 - [FCG2A_HUMAN]	5,99	2
P01130	Low-density lipoprotein receptor OS=Homo sapiens GN=LDLR PE=1 SV=1 - [LDLR_HUMAN]	1,51	1

P51884	Lumican OS=Homo sapiens GN=LUM PE=1 SV=2 - [LUM_HUMAN]	5,92	2
Q5SQ64	Lymphocyte antigen 6 complex locus protein G6f OS=Homo sapiens GN=LY6G6F PE=1 SV=2 - [LY66F_HUMAN]	6,06	1
P19256	Lymphocyte function-associated antigen 3 OS=Homo sapiens GN=CD58 PE=1 SV=1 - [LFA3_HUMAN]	7,20	2
Q12912	Lymphoid-restricted membrane protein OS=Homo sapiens GN=LRMP PE=1 SV=3 - [LRMP_HUMAN]	2,70	1
Q6P1A2	Lysophospholipid acyltransferase 5 OS=Homo sapiens GN=LPCAT3 PE=1 SV=1 - [MBOA5_HUMAN]	8,83	1
P10619	Lysosomal protective protein OS=Homo sapiens GN=CTSA PE=1 SV=2 - [PPGB_HUMAN]	6,67	1
P11279	Lysosome-associated membrane glycoprotein 1 OS=Homo sapiens GN=LAMP1 PE=1 SV=3 - [LAMP1_HUMAN]	8,63	4
P61626	Lysozyme C OS=Homo sapiens GN=LYZ PE=1 SV=1 - [LYSC_HUMAN]	14,19	1
Q9H3U5	Major facilitator superfamily domain-containing protein 1 OS=Homo sapiens GN=MFSD1 PE=2 SV=2 - [MFSD1_HUMAN]	4,95	2
Q14728	Major facilitator superfamily domain-containing protein 10 OS=Homo sapiens GN=MFSD10 PE=2 SV=1 - [MFS10_HUMAN]	6,59	1
A6NFX1	Major facilitator superfamily domain-containing protein 2B OS=Homo sapiens GN=MFSD2B PE=2 SV=3 - [MFS2B_HUMAN]	6,84	1
Q6ZSS7	Major facilitator superfamily domain-containing protein 6 OS=Homo sapiens GN=MFSD6 PE=1 SV=2 - [MFSD6_HUMAN]	1,77	1
P04156	Major prion protein OS=Homo sapiens GN=PRNP PE=1 SV=1 - [PRIO_HUMAN]	3,16	1
Q14764	Major vault protein OS=Homo sapiens GN=MVP PE=1 SV=4 - [MVP_HUMAN]	6,05	4
P40926	Malate dehydrogenase, mitochondrial OS=Homo sapiens GN=MDH2 PE=1 SV=3 - [MDHM_HUMAN]	17,75	4
Q14165	Malectin OS=Homo sapiens GN=MLEC PE=1 SV=1 - [MLEC_HUMAN]	29,11	4
P48740	Mannan-binding lectin serine protease 1 OS=Homo sapiens GN=MASP1 PE=1 SV=3 - [MASP1_HUMAN]	2,72	1
P11226	Mannose-binding protein C OS=Homo sapiens GN=MBL2 PE=1 SV=2 - [MBL2_HUMAN]	25,81	5
P11310	Medium-chain specific acyl-CoA dehydrogenase, mitochondrial OS=Homo sapiens GN=ACADM PE=1 SV=1 - [ACADM_HUMAN]	5,23	2
Q5JRA6	Melanoma inhibitory activity protein 3 OS=Homo sapiens GN=MIA3 PE=1 SV=1 - [MIA3_HUMAN]	0,63	1
P15529	Membrane cofactor protein OS=Homo sapiens GN=CD46 PE=1 SV=3 - [MCP_HUMAN]	2,04	1
Q8TBP5	Membrane protein FAM174A OS=Homo sapiens GN=FAM174A PE=2 SV=1 - [F174A_HUMAN]	4,74	1
O00264	Membrane-associated progesterone receptor component 1 OS=Homo sapiens GN=PGRMC1 PE=1 SV=3 - [PGRC1_HUMAN]	30,26	3
O15173	Membrane-associated progesterone receptor component 2 OS=Homo sapiens GN=PGRMC2 PE=1 SV=1 - [PGRC2_HUMAN]	12,56	1
P55145	Mesencephalic astrocyte-derived neurotrophic factor OS=Homo sapiens GN=MANF PE=1 SV=3 - [MANF_HUMAN]	18,68	1
P01033	Metalloproteinase inhibitor 1 OS=Homo sapiens GN=TIMP1 PE=1 SV=1 - [TIMP1_HUMAN]	5,80	1
Q96RQ3	Methylcrotonoyl-CoA carboxylase subunit alpha, mitochondrial OS=Homo sapiens GN=MCCC1 PE=1 SV=3 - [MCCA_HUMAN]	1,52	1
Q02252	Methylmalonate-semialdehyde dehydrogenase [acylating], mitochondrial OS=Homo sapiens GN=ALDH6A1 PE=1 SV=2 - [MMSA_HU	5,23	2
Q9UPN3	Microtubule-actin cross-linking factor 1, isoforms 1/2/3/5 OS=Homo sapiens GN=MACF1 PE=1 SV=4 - [MACF1_HUMAN]	0,66	3
Q8TCT9	Minor histocompatibility antigen H13 OS=Homo sapiens GN=HM13 PE=1 SV=1 - [HM13_HUMAN]	5,84	1
Q92619	Minor histocompatibility protein HA-1 OS=Homo sapiens GN=HMHA1 PE=1 SV=2 - [HMHA1_HUMAN]	2,46	2
Q02978	Mitochondrial 2-oxoglutarate/malate carrier protein OS=Homo sapiens GN=SLC25A11 PE=1 SV=3 - [M2OM_HUMAN]	14,97	3
Q7Z434	Mitochondrial antiviral-signaling protein OS=Homo sapiens GN=MAVS PE=1 SV=2 - [MAVS_HUMAN]	5,19	2
Q9Y3D6	Mitochondrial fission 1 protein OS=Homo sapiens GN=FIS1 PE=1 SV=2 - [FIS1_HUMAN]	8,55	1
O43615	Mitochondrial import inner membrane translocase subunit TIM44 OS=Homo sapiens GN=TIMM44 PE=1 SV=2 - [TIM44_HUMAN]	3,76	2
Q3ZCQ8	Mitochondrial import inner membrane translocase subunit TIM50 OS=Homo sapiens GN=TIMM50 PE=1 SV=2 - [TIM50_HUMAN]	4,82	1
Q9NS69	Mitochondrial import receptor subunit TOM22 homolog OS=Homo sapiens GN=TOMM22 PE=1 SV=3 - [TOM22_HUMAN]	8,45	1
Q8N4H5	Mitochondrial import receptor subunit TOM5 homolog OS=Homo sapiens GN=TOMM5 PE=1 SV=1 - [TOM5_HUMAN]	13,73	1
O94826	Mitochondrial import receptor subunit TOM70 OS=Homo sapiens GN=TOMM70A PE=1 SV=1 - [TOM70_HUMAN]	1,15	1
Q5TGZ0	Mitochondrial inner membrane organizing system protein 1 OS=Homo sapiens GN=MINOS1 PE=1 SV=1 - [MOS1_HUMAN]	10,26	1

Q16891	Mitochondrial inner membrane protein OS=Homo sapiens GN=IMMT PE=1 SV=1 - [IMMT_HUMAN]	13,19	10
Q99683	Mitogen-activated protein kinase kinase kinase 5 OS=Homo sapiens GN=MAP3K5 PE=1 SV=1 - [M3K5_HUMAN]	0,80	1
P26038	Moesin OS=Homo sapiens GN=MSN PE=1 SV=3 - [MOES_HUMAN]	47,31	16
O15427	Monocarboxylate transporter 4 OS=Homo sapiens GN=SLC16A3 PE=1 SV=1 - [MOT4_HUMAN]	9,03	1
Q765P7	MTSS1-like protein OS=Homo sapiens GN=MTSS1L PE=1 SV=1 - [MTSSL_HUMAN]	1,20	1
Q96DR8	Mucin-like protein 1 OS=Homo sapiens GN=MUCL1 PE=1 SV=1 - [MUCL1_HUMAN]	18,89	2
O15439	Multidrug resistance-associated protein 4 OS=Homo sapiens GN=ABCC4 PE=1 SV=3 - [MRP4_HUMAN]	8,08	3
Q13201	Multimerin-1 OS=Homo sapiens GN=MMRN1 PE=1 SV=3 - [MMRN1_HUMAN]	21,17	19
Q6DN14	Multiple C2 and transmembrane domain-containing protein 1 OS=Homo sapiens GN=MCTP1 PE=2 SV=2 - [MCTP1_HUMAN]	1,20	1
Q8N699	Myc target protein 1 OS=Homo sapiens GN=MYCT1 PE=2 SV=1 - [MYCT1_HUMAN]	22,13	1
Q96S97	Myeloid-associated differentiation marker OS=Homo sapiens GN=MYADM PE=1 SV=2 - [MYADM_HUMAN]	12,73	2
P05164	Myeloperoxidase OS=Homo sapiens GN=MPO PE=1 SV=1 - [PERM_HUMAN]	10,20	2
P60660	Myosin light polypeptide 6 OS=Homo sapiens GN=MYL6 PE=1 SV=2 - [MYL6_HUMAN]	52,32	7
P19105	Myosin regulatory light chain 12A OS=Homo sapiens GN=MYL12A PE=1 SV=2 - [ML12A_HUMAN]	29,82	5
P24844	Myosin regulatory light polypeptide 9 OS=Homo sapiens GN=MYL9 PE=1 SV=4 - [MYL9_HUMAN]	29,65	4
Q7Z406	Myosin-14 OS=Homo sapiens GN=MYH14 PE=1 SV=2 - [MYH14_HUMAN]	4,11	9
P35579	Myosin-9 OS=Homo sapiens GN=MYH9 PE=1 SV=4 - [MYH9_HUMAN]	48,37	86
P29966	Myristoylated alanine-rich C-kinase substrate OS=Homo sapiens GN=MARCKS PE=1 SV=4 - [MARCS_HUMAN]	4,52	1
O14745	Na(+)/H(+) exchange regulatory cofactor NHE-RF1 OS=Homo sapiens GN=SLC9A3R1 PE=1 SV=4 - [NHRF1_HUMAN]	17,88	4
Q13423	NAD(P) transhydrogenase, mitochondrial OS=Homo sapiens GN=NNT PE=1 SV=3 - [NNTM_HUMAN]	18,05	15
P23368	NAD-dependent malic enzyme, mitochondrial OS=Homo sapiens GN=ME2 PE=1 SV=1 - [MAOM_HUMAN]	21,75	3
Q86Y39	NADH dehydrogenase [ubiquinone] 1 alpha subcomplex subunit 11 OS=Homo sapiens GN=NDUFA11 PE=1 SV=3 - [NDUAB_HUMA	24,11	1
Q9P0J0	NADH dehydrogenase [ubiquinone] 1 alpha subcomplex subunit 13 OS=Homo sapiens GN=NDUFA13 PE=1 SV=3 - [NDUAD_HUMA	6,25	1
O43678	NADH dehydrogenase [ubiquinone] 1 alpha subcomplex subunit 2 OS=Homo sapiens GN=NDUFA2 PE=1 SV=3 - [NDUA2_HUMAN]	10,10	1
O00483	NADH dehydrogenase [ubiquinone] 1 alpha subcomplex subunit 4 OS=Homo sapiens GN=NDUFA4 PE=1 SV=1 - [NDUA4_HUMAN]	9,88	1
Q16718	NADH dehydrogenase [ubiquinone] 1 alpha subcomplex subunit 5 OS=Homo sapiens GN=NDUFA5 PE=1 SV=3 - [NDUA5_HUMAN]	16,38	2
O95182	NADH dehydrogenase [ubiquinone] 1 alpha subcomplex subunit 7 OS=Homo sapiens GN=NDUFA7 PE=1 SV=3 - [NDUA7_HUMAN]	15,93	1
O96000	NADH dehydrogenase [ubiquinone] 1 beta subcomplex subunit 10 OS=Homo sapiens GN=NDUFB10 PE=1 SV=3 - [NDUBA_HUMAN	12,79	1
Q9NX14	NADH dehydrogenase [ubiquinone] 1 beta subcomplex subunit 11, mitochondrial OS=Homo sapiens GN=NDUFB11 PE=1 SV=1 - [N	7,19	1
O43676	NADH dehydrogenase [ubiquinone] 1 beta subcomplex subunit 3 OS=Homo sapiens GN=NDUFB3 PE=1 SV=3 - [NDUB3_HUMAN]	11,22	1
P17568	NADH dehydrogenase [ubiquinone] 1 beta subcomplex subunit 7 OS=Homo sapiens GN=NDUFB7 PE=1 SV=4 - [NDUB7_HUMAN]	10,95	1
P19404	NADH dehydrogenase [ubiquinone] flavoprotein 2, mitochondrial OS=Homo sapiens GN=NDUFV2 PE=1 SV=2 - [NDUV2_HUMAN]	5,22	1
O75489	NADH dehydrogenase [ubiquinone] iron-sulfur protein 3, mitochondrial OS=Homo sapiens GN=NDUFS3 PE=1 SV=1 - [NDUS3_HUM	4,92	1
O75251	NADH dehydrogenase [ubiquinone] iron-sulfur protein 7, mitochondrial OS=Homo sapiens GN=NDUFS7 PE=1 SV=3 - [NDUS7_HUM	10,80	2
Q9UHQ9	NADH-cytochrome b5 reductase 1 OS=Homo sapiens GN=CYB5R1 PE=1 SV=1 - [NB5R1_HUMAN]	12,46	3
P00387	NADH-cytochrome b5 reductase 3 OS=Homo sapiens GN=CYB5R3 PE=1 SV=3 - [NB5R3_HUMAN]	43,19	3
P28331	NADH-ubiquinone oxidoreductase 75 kDa subunit, mitochondrial OS=Homo sapiens GN=NDUFS1 PE=1 SV=3 - [NDUS1_HUMAN]	3,71	3
P16435	NADPH--cytochrome P450 reductase OS=Homo sapiens GN=POR PE=1 SV=2 - [NCPR_HUMAN]	2,36	1
Q9Y2A7	Nck-associated protein 1 OS=Homo sapiens GN=NCKAP1 PE=1 SV=1 - [NCKP1_HUMAN]	7,62	2
P13591	Neural cell adhesion molecule 1 OS=Homo sapiens GN=NCAM1 PE=1 SV=3 - [NCAM1_HUMAN]	5,24	3

Q14697	Neutral alpha-glucosidase AB OS=Homo sapiens GN=GANAB PE=1 SV=3 - [GANAB_HUMAN]	18,86	6
P59665	Neutrophil defensin 1 OS=Homo sapiens GN=DEFA1 PE=1 SV=1 - [DEF1_HUMAN]	9,57	1
P80188	Neutrophil gelatinase-associated lipocalin OS=Homo sapiens GN=LCN2 PE=1 SV=2 - [NGAL_HUMAN]	7,58	1
Q0ZGT2	Nexilin OS=Homo sapiens GN=NEXN PE=1 SV=1 - [NEXN_HUMAN]	6,67	4
Q969V3	Nicalin OS=Homo sapiens GN=NCLN PE=1 SV=2 - [NCLN_HUMAN]	3,20	2
P14543	Nidogen-1 OS=Homo sapiens GN=NID1 PE=1 SV=3 - [NID1_HUMAN]	8,82	8
Q14112	Nidogen-2 OS=Homo sapiens GN=NID2 PE=1 SV=3 - [NID2_HUMAN]	1,24	1
Q86UT6	NLR family member X1 OS=Homo sapiens GN=NLRX1 PE=1 SV=1 - [NLRX1_HUMAN]	5,23	5
P69849	Nodal modulator 3 OS=Homo sapiens GN=NOMO3 PE=2 SV=2 - [NOMO3_HUMAN]	2,78	2
P80303	Nucleobindin-2 OS=Homo sapiens GN=NUCB2 PE=1 SV=2 - [NUCB2_HUMAN]	5,24	2
P55209	Nucleosome assembly protein 1-like 1 OS=Homo sapiens GN=NAP1L1 PE=1 SV=1 - [NP1L1_HUMAN]	11,51	1
Q99733	Nucleosome assembly protein 1-like 4 OS=Homo sapiens GN=NAP1L4 PE=1 SV=1 - [NP1L4_HUMAN]	5,60	1
Q8WWZ8	Oncoprotein-induced transcript 3 protein OS=Homo sapiens GN=OIT3 PE=1 SV=2 - [OIT3_HUMAN]	16,15	5
O75665	Oral-facial-digital syndrome 1 protein OS=Homo sapiens GN=OFD1 PE=1 SV=1 - [OFD1_HUMAN]	0,69	1
Q53FV1	ORM1-like protein 2 OS=Homo sapiens GN=ORMDL2 PE=2 SV=2 - [ORML2_HUMAN]	7,19	1
Q9BZF1	Oxysterol-binding protein-related protein 8 OS=Homo sapiens GN=OSBPL8 PE=1 SV=3 - [OSBL8_HUMAN]	1,24	1
P51575	P2X purinoceptor 1 OS=Homo sapiens GN=P2RX1 PE=1 SV=1 - [P2RX1_HUMAN]	14,04	3
Q9H244	P2Y purinoceptor 12 OS=Homo sapiens GN=P2RY12 PE=1 SV=1 - [P2Y12_HUMAN]	4,97	1
O00151	PDZ and LIM domain protein 1 OS=Homo sapiens GN=PDLIM1 PE=1 SV=4 - [PDLI1_HUMAN]	12,46	2
P62937	Peptidyl-prolyl cis-trans isomerase A OS=Homo sapiens GN=PPIA PE=1 SV=2 - [PPIA_HUMAN]	43,64	5
P23284	Peptidyl-prolyl cis-trans isomerase B OS=Homo sapiens GN=PPIB PE=1 SV=2 - [PPIB_HUMAN]	44,44	4
P30405	Peptidyl-prolyl cis-trans isomerase F, mitochondrial OS=Homo sapiens GN=PPIF PE=1 SV=1 - [PPIF_HUMAN]	16,91	3
O60437	Periplakin OS=Homo sapiens GN=PPL PE=1 SV=4 - [PEPL_HUMAN]	0,40	1
Q06830	Peroxiredoxin-1 OS=Homo sapiens GN=PRDX1 PE=1 SV=1 - [PRDX1_HUMAN]	36,68	6
P32119	Peroxiredoxin-2 OS=Homo sapiens GN=PRDX2 PE=1 SV=5 - [PRDX2_HUMAN]	19,70	4
Q13162	Peroxiredoxin-4 OS=Homo sapiens GN=PRDX4 PE=1 SV=1 - [PRDX4_HUMAN]	23,25	3
P30044	Peroxiredoxin-5, mitochondrial OS=Homo sapiens GN=PRDX5 PE=1 SV=4 - [PRDX5_HUMAN]	8,41	1
P30041	Peroxiredoxin-6 OS=Homo sapiens GN=PRDX6 PE=1 SV=3 - [PRDX6_HUMAN]	19,64	1
Q15067	Peroxisomal acyl-coenzyme A oxidase 1 OS=Homo sapiens GN=ACOX1 PE=1 SV=3 - [ACOX1_HUMAN]	1,36	1
P51659	Peroxisomal multifunctional enzyme type 2 OS=Homo sapiens GN=HSD17B4 PE=1 SV=3 - [DHB4_HUMAN]	29,48	8
O95571	Persulfide dioxygenase ETHE1, mitochondrial OS=Homo sapiens GN=ETHE1 PE=1 SV=2 - [ETHE1_HUMAN]	5,51	1
Q00325	Phosphate carrier protein, mitochondrial OS=Homo sapiens GN=SLC25A3 PE=1 SV=2 - [MPCP_HUMAN]	15,75	3
O95674	Phosphatidate cytidylyltransferase 2 OS=Homo sapiens GN=CDS2 PE=1 SV=1 - [CDS2_HUMAN]	8,76	3
P30086	Phosphatidylethanolamine-binding protein 1 OS=Homo sapiens GN=PEBP1 PE=1 SV=3 - [PEBP1_HUMAN]	22,46	1
Q9NTJ5	Phosphatidylinositide phosphatase SAC1 OS=Homo sapiens GN=SACM1L PE=1 SV=2 - [SAC1_HUMAN]	17,38	10
P42356	Phosphatidylinositol 4-kinase alpha OS=Homo sapiens GN=PI4KA PE=1 SV=4 - [PI4KA_HUMAN]	1,62	2
P48426	Phosphatidylinositol 5-phosphate 4-kinase type-2 alpha OS=Homo sapiens GN=PIP4K2A PE=1 SV=2 - [PI42A_HUMAN]	6,16	1
Q9BVG9	Phosphatidylserine synthase 2 OS=Homo sapiens GN=PTDSS2 PE=1 SV=1 - [PTSS2_HUMAN]	6,16	1
P36871	Phosphoglucomutase-1 OS=Homo sapiens GN=PGM1 PE=1 SV=3 - [PGM1_HUMAN]	2,14	1
P00558	Phosphoglycerate kinase 1 OS=Homo sapiens GN=PGK1 PE=1 SV=3 - [PGK1_HUMAN]	13,91	1

P36955	Pigment epithelium-derived factor OS=Homo sapiens GN=SERPINF1 PE=1 SV=4 - [PEDF_HUMAN]	4,31	2
P53801	Pituitary tumor-transforming gene 1 protein-interacting protein OS=Homo sapiens GN=PTTG1IP PE=1 SV=1 - [PTTG_HUMAN]	6,67	1
P03952	Plasma kallikrein OS=Homo sapiens GN=KLKB1 PE=1 SV=1 - [KLKB1_HUMAN]	20,06	2
P20020	Plasma membrane calcium-transporting ATPase 1 OS=Homo sapiens GN=ATP2B1 PE=1 SV=3 - [AT2B1_HUMAN]	2,86	1
P23634	Plasma membrane calcium-transporting ATPase 4 OS=Homo sapiens GN=ATP2B4 PE=1 SV=2 - [AT2B4_HUMAN]	4,19	2
P05155	Plasma protease C1 inhibitor OS=Homo sapiens GN=SERPING1 PE=1 SV=2 - [IC1_HUMAN]	11,00	4
P05121	Plasminogen activator inhibitor 1 OS=Homo sapiens GN=SERPINE1 PE=1 SV=1 - [PAI1_HUMAN]	4,73	1
P00747	Plasminogen OS=Homo sapiens GN=PLG PE=1 SV=2 - [PLMN_HUMAN]	19,51	4
Q9HBL7	Plasminogen receptor (KT) OS=Homo sapiens GN=PLGRKT PE=1 SV=1 - [PLRKT_HUMAN]	8,84	1
P13796	Plastin-2 OS=Homo sapiens GN=LCP1 PE=1 SV=6 - [PLSL_HUMAN]	8,61	1
P02775	Platelet basic protein OS=Homo sapiens GN=PPBP PE=1 SV=3 - [CXCL7_HUMAN]	45,31	5
Q5VY43	Platelet endothelial aggregation receptor 1 OS=Homo sapiens GN=PEAR1 PE=1 SV=1 - [PEAR1_HUMAN]	1,83	2
P16284	Platelet endothelial cell adhesion molecule OS=Homo sapiens GN=PECAM1 PE=1 SV=1 - [PECA1_HUMAN]	28,32	14
P02776	Platelet factor 4 OS=Homo sapiens GN=PF4 PE=1 SV=2 - [PLF4_HUMAN]	36,63	2
P16671	Platelet glycoprotein 4 OS=Homo sapiens GN=CD36 PE=1 SV=2 - [CD36_HUMAN]	18,22	6
P07359	Platelet glycoprotein Ib alpha chain OS=Homo sapiens GN=GP1BA PE=1 SV=2 - [GP1BA_HUMAN]	18,25	9
P13224	Platelet glycoprotein Ib beta chain OS=Homo sapiens GN=GP1BB PE=1 SV=1 - [GP1BB_HUMAN]	19,42	3
P14770	Platelet glycoprotein IX OS=Homo sapiens GN=GP9 PE=1 SV=3 - [GPIX_HUMAN]	35,59	3
P40197	Platelet glycoprotein V OS=Homo sapiens GN=GP5 PE=1 SV=1 - [GPV_HUMAN]	17,50	6
Q9HCN6	Platelet glycoprotein VI OS=Homo sapiens GN=GP6 PE=1 SV=4 - [GPVI_HUMAN]	7,37	2
Q9H7M9	Platelet receptor Gi24 OS=Homo sapiens GN=C10orf54 PE=1 SV=3 - [GI24_HUMAN]	11,25	2
P08567	Pleckstrin OS=Homo sapiens GN=PLEK PE=1 SV=3 - [PLEK_HUMAN]	7,71	1
Q15149	Plectin OS=Homo sapiens GN=PLEC PE=1 SV=3 - [PLEC_HUMAN]	14,24	42
Q6UX71	Plexin domain-containing protein 2 OS=Homo sapiens GN=PLXDC2 PE=1 SV=1 - [PXDC2_HUMAN]	6,24	2
Q9HCM2	Plexin-A4 OS=Homo sapiens GN=PLXNA4 PE=1 SV=4 - [PLXA4_HUMAN]	4,65	4
O15031	Plexin-B2 OS=Homo sapiens GN=PLXNB2 PE=1 SV=3 - [PLXB2_HUMAN]	7,13	7
Q9ULL4	Plexin-B3 OS=Homo sapiens GN=PLXNB3 PE=1 SV=2 - [PLXB3_HUMAN]	2,67	3
O00592	Podocalyxin OS=Homo sapiens GN=PODXL PE=1 SV=2 - [PODXL_HUMAN]	3,94	2
Q92692	Poliovirus receptor-related protein 2 OS=Homo sapiens GN=PVRL2 PE=1 SV=1 - [PVRL2_HUMAN]	6,13	1
Q15365	Poly(rC)-binding protein 1 OS=Homo sapiens GN=PCBP1 PE=1 SV=2 - [PCBP1_HUMAN]	5,62	2
P01833	Polymeric immunoglobulin receptor OS=Homo sapiens GN=PIGR PE=1 SV=4 - [PIGR_HUMAN]	18,59	1
Q10472	Polypeptide N-acetylgalactosaminyltransferase 1 OS=Homo sapiens GN=GALNT1 PE=1 SV=1 - [GALT1_HUMAN]	2,15	1
P0CG48	Polyubiquitin-C OS=Homo sapiens GN=UBC PE=1 SV=3 - [UBC_HUMAN]	49,93	1
O60831	PRA1 family protein 2 OS=Homo sapiens GN=PRAF2 PE=1 SV=1 - [PRAF2_HUMAN]	6,18	1
O75915	PRA1 family protein 3 OS=Homo sapiens GN=ARL6IP5 PE=1 SV=1 - [PRAF3_HUMAN]	9,57	1
P20742	Pregnancy zone protein OS=Homo sapiens GN=PZP PE=1 SV=4 - [PZP_HUMAN]	7,09	3
P02545	Prelamin-A/C OS=Homo sapiens GN=LMNA PE=1 SV=1 - [LMNA_HUMAN]	3,16	2
Q9HD20	Probable cation-transporting ATPase 13A1 OS=Homo sapiens GN=ATP13A1 PE=1 SV=2 - [AT131_HUMAN]	3,65	2
Q9Y2Q0	Probable phospholipid-transporting ATPase IA OS=Homo sapiens GN=ATP8A1 PE=1 SV=1 - [AT8A1_HUMAN]	20,02	3
Q9Y2G3	Probable phospholipid-transporting ATPase IF OS=Homo sapiens GN=ATP11B PE=1 SV=2 - [AT11B_HUMAN]	4,08	3

O43861	Probable phospholipid-transporting ATPase IIB OS=Homo sapiens GN=ATP9B PE=2 SV=4 - [ATP9B_HUMAN]	0,87	1
P09668	Pro-cathepsin H OS=Homo sapiens GN=CTSH PE=1 SV=4 - [CATH_HUMAN]	3,88	1
P01133	Pro-epidermal growth factor OS=Homo sapiens GN=EGF PE=1 SV=2 - [EGF_HUMAN]	2,90	3
P07737	Profilin-1 OS=Homo sapiens GN=PFN1 PE=1 SV=2 - [PROF1_HUMAN]	47,86	4
Q8WUM4	Programmed cell death 6-interacting protein OS=Homo sapiens GN=PDCD6IP PE=1 SV=1 - [PDC6I_HUMAN]	26,84	4
P35232	Prohibitin OS=Homo sapiens GN=PHB PE=1 SV=1 - [PHB_HUMAN]	39,34	8
Q99623	Prohibitin-2 OS=Homo sapiens GN=PHB2 PE=1 SV=2 - [PHB2_HUMAN]	36,45	4
P12273	Prolactin-inducible protein OS=Homo sapiens GN=PIP PE=1 SV=1 - [PIP_HUMAN]	25,34	2
Q9H939	Proline-serine-threonine phosphatase-interacting protein 2 OS=Homo sapiens GN=PSTPIP2 PE=1 SV=4 - [PPIP2_HUMAN]	3,29	1
Q32P28	Prolyl 3-hydroxylase 1 OS=Homo sapiens GN=LEPRE1 PE=1 SV=2 - [P3H1_HUMAN]	1,36	1
P27918	Properdin OS=Homo sapiens GN=CFP PE=1 SV=2 - [PROP_HUMAN]	4,69	1
P05165	Propionyl-CoA carboxylase alpha chain, mitochondrial OS=Homo sapiens GN=PCCA PE=1 SV=4 - [PCCA_HUMAN]	3,30	2
P05166	Propionyl-CoA carboxylase beta chain, mitochondrial OS=Homo sapiens GN=PCCB PE=1 SV=3 - [PCCB_HUMAN]	5,75	2
P23219	Prostaglandin G/H synthase 1 OS=Homo sapiens GN=PTGS1 PE=1 SV=2 - [PGH1_HUMAN]	21,54	6
P25786	Proteasome subunit alpha type-1 OS=Homo sapiens GN=PSMA1 PE=1 SV=1 - [PSA1_HUMAN]	10,65	1
P25788	Proteasome subunit alpha type-3 OS=Homo sapiens GN=PSMA3 PE=1 SV=2 - [PSA3_HUMAN]	8,24	1
P25789	Proteasome subunit alpha type-4 OS=Homo sapiens GN=PSMA4 PE=1 SV=1 - [PSA4_HUMAN]	10,34	2
P28066	Proteasome subunit alpha type-5 OS=Homo sapiens GN=PSMA5 PE=1 SV=3 - [PSA5_HUMAN]	4,15	1
P60900	Proteasome subunit alpha type-6 OS=Homo sapiens GN=PSMA6 PE=1 SV=1 - [PSA6_HUMAN]	9,35	2
O14818	Proteasome subunit alpha type-7 OS=Homo sapiens GN=PSMA7 PE=1 SV=1 - [PSA7_HUMAN]	13,71	2
P49721	Proteasome subunit beta type-2 OS=Homo sapiens GN=PSMB2 PE=1 SV=1 - [PSB2_HUMAN]	4,98	1
P28070	Proteasome subunit beta type-4 OS=Homo sapiens GN=PSMB4 PE=1 SV=4 - [PSB4_HUMAN]	3,79	1
P28072	Proteasome subunit beta type-6 OS=Homo sapiens GN=PSMB6 PE=1 SV=4 - [PSB6_HUMAN]	8,79	2
Q99436	Proteasome subunit beta type-7 OS=Homo sapiens GN=PSMB7 PE=1 SV=1 - [PSB7_HUMAN]	3,61	1
P28065	Proteasome subunit beta type-9 OS=Homo sapiens GN=PSMB9 PE=1 SV=2 - [PSB9_HUMAN]	4,57	1
P11171	Protein 4.1 OS=Homo sapiens GN=EPB41 PE=1 SV=4 - [41_HUMAN]	10,88	3
P02760	Protein AMBP OS=Homo sapiens GN=AMBP PE=1 SV=1 - [AMBP_HUMAN]	11,36	1
Q9Y2B0	Protein canopy homolog 2 OS=Homo sapiens GN=CNPY2 PE=1 SV=1 - [CNPY2_HUMAN]	5,49	1
O60610	Protein diaphanous homolog 1 OS=Homo sapiens GN=DIAPH1 PE=1 SV=2 - [DIAP1_HUMAN]	4,64	5
P30101	Protein disulfide-isomerase A3 OS=Homo sapiens GN=PDIA3 PE=1 SV=4 - [PDIA3_HUMAN]	37,43	9
P13667	Protein disulfide-isomerase A4 OS=Homo sapiens GN=PDIA4 PE=1 SV=2 - [PDIA4_HUMAN]	26,20	11
Q14554	Protein disulfide-isomerase A5 OS=Homo sapiens GN=PDIA5 PE=1 SV=1 - [PDIA5_HUMAN]	15,99	2
Q15084	Protein disulfide-isomerase A6 OS=Homo sapiens GN=PDIA6 PE=1 SV=1 - [PDIA6_HUMAN]	18,86	5
P07237	Protein disulfide-isomerase OS=Homo sapiens GN=P4HB PE=1 SV=3 - [PDIA1_HUMAN]	30,91	9
Q96JJ7	Protein disulfide-isomerase TMX3 OS=Homo sapiens GN=TMX3 PE=1 SV=2 - [TMX3_HUMAN]	12,33	1
Q99497	Protein DJ-1 OS=Homo sapiens GN=PARK7 PE=1 SV=2 - [PARK7_HUMAN]	33,86	2
Q14156	Protein EFR3 homolog A OS=Homo sapiens GN=EFR3A PE=1 SV=2 - [EFR3A_HUMAN]	2,80	1
P49257	Protein ERGIC-53 OS=Homo sapiens GN=LMAN1 PE=1 SV=2 - [LMAN1_HUMAN]	9,80	3
Q8N128	Protein FAM177A1 OS=Homo sapiens GN=FAM177A1 PE=1 SV=1 - [F177A_HUMAN]	4,69	1
P98173	Protein FAM3A OS=Homo sapiens GN=FAM3A PE=1 SV=2 - [FAM3A_HUMAN]	5,22	1

Q92520	Protein FAM3C OS=Homo sapiens GN=FAM3C PE=1 SV=1 - [FAM3C_HUMAN]	12,33	1
Q9NUQ9	Protein FAM49B OS=Homo sapiens GN=FAM49B PE=1 SV=1 - [FA49B_HUMAN]	5,86	1
Q9ULI3	Protein HEG homolog 1 OS=Homo sapiens GN=HEG1 PE=1 SV=3 - [HEG1_HUMAN]	2,17	1
Q8N5M9	Protein jagunal homolog 1 OS=Homo sapiens GN=JAGN1 PE=1 SV=1 - [JAGN1_HUMAN]	6,56	1
Q9UNF0	Protein kinase C and casein kinase substrate in neurons protein 2 OS=Homo sapiens GN=PACSIN2 PE=1 SV=2 - [PACN2_HUMAN]	4,12	2
P05771	Protein kinase C beta type OS=Homo sapiens GN=PRKCB PE=1 SV=4 - [KPCB_HUMAN]	6,71	1
Q05655	Protein kinase C delta type OS=Homo sapiens GN=PRKCD PE=1 SV=2 - [KPCD_HUMAN]	2,51	1
Q96RT1	Protein LAP2 OS=Homo sapiens GN=ERBB2IP PE=1 SV=2 - [LAP2_HUMAN]	2,48	2
Q86UE4	Protein LYRIC OS=Homo sapiens GN=MTDH PE=1 SV=2 - [LYRIC_HUMAN]	3,09	1
Q9Y6F6	Protein MRVI1 OS=Homo sapiens GN=MRVI1 PE=1 SV=2 - [MRVI1_HUMAN]	4,75	4
Q9UFN0	Protein NipSnap homolog 3A OS=Homo sapiens GN=NIPSNAP3A PE=1 SV=2 - [NPS3A_HUMAN]	21,86	1
P35813	Protein phosphatase 1A OS=Homo sapiens GN=PPM1A PE=1 SV=1 - [PPM1A_HUMAN]	6,28	2
Q8WVV4	Protein POF1B OS=Homo sapiens GN=POF1B PE=1 SV=3 - [POF1B_HUMAN]	1,70	1
O15258	Protein RER1 OS=Homo sapiens GN=RER1 PE=1 SV=1 - [RER1_HUMAN]	9,18	1
P31949	Protein S100-A11 OS=Homo sapiens GN=S100A11 PE=1 SV=2 - [S10AB_HUMAN]	8,57	1
Q9HCY8	Protein S100-A14 OS=Homo sapiens GN=S100A14 PE=1 SV=1 - [S10AE_HUMAN]	10,58	1
P26447	Protein S100-A4 OS=Homo sapiens GN=S100A4 PE=1 SV=1 - [S10A4_HUMAN]	18,81	1
P06703	Protein S100-A6 OS=Homo sapiens GN=S100A6 PE=1 SV=1 - [S10A6_HUMAN]	16,67	2
P31151	Protein S100-A7 OS=Homo sapiens GN=S100A7 PE=1 SV=4 - [S10A7_HUMAN]	23,76	2
P05109	Protein S100-A8 OS=Homo sapiens GN=S100A8 PE=1 SV=1 - [S10A8_HUMAN]	48,39	1
P25815	Protein S100-P OS=Homo sapiens GN=S100P PE=1 SV=2 - [S100P_HUMAN]	13,68	1
Q8TF72	Protein Shroom3 OS=Homo sapiens GN=SHROOM3 PE=1 SV=2 - [SHRM3_HUMAN]	0,45	1
Q69YW2	Protein stum homolog OS=Homo sapiens GN=C1orf95 PE=2 SV=1 - [STUM_HUMAN]	9,22	1
Q92734	Protein TFG OS=Homo sapiens GN=TFG PE=1 SV=2 - [TFG_HUMAN]	4,50	2
Q8WUY1	Protein THEM6 OS=Homo sapiens GN=THEM6 PE=1 SV=2 - [THEM6_HUMAN]	13,46	1
P61619	Protein transport protein Sec61 subunit alpha isoform 1 OS=Homo sapiens GN=SEC61A1 PE=1 SV=2 - [S61A1_HUMAN]	4,62	2
P60468	Protein transport protein Sec61 subunit beta OS=Homo sapiens GN=SEC61B PE=1 SV=2 - [SC61B_HUMAN]	37,50	3
Q9C0H2	Protein tweety homolog 3 OS=Homo sapiens GN=TTYH3 PE=1 SV=3 - [TTYH3_HUMAN]	10,33	2
Q70J99	Protein unc-13 homolog D OS=Homo sapiens GN=UNC13D PE=1 SV=1 - [UN13D_HUMAN]	6,33	3
Q9H3U1	Protein unc-45 homolog A OS=Homo sapiens GN=UNC45A PE=1 SV=1 - [UN45A_HUMAN]	1,27	1
P22735	Protein-glutamine gamma-glutamyltransferase K OS=Homo sapiens GN=TGM1 PE=1 SV=4 - [TGM1_HUMAN]	9,79	6
O60704	Protein-tyrosine sulfotransferase 2 OS=Homo sapiens GN=TPST2 PE=1 SV=1 - [TPST2_HUMAN]	5,04	1
Q92954	Proteoglycan 4 OS=Homo sapiens GN=PRG4 PE=1 SV=2 - [PRG4_HUMAN]	0,50	1
Q04941	Proteolipid protein 2 OS=Homo sapiens GN=PLP2 PE=1 SV=1 - [PLP2_HUMAN]	8,55	1
P00734	Prothrombin OS=Homo sapiens GN=F2 PE=1 SV=2 - [THRB_HUMAN]	10,45	5
P12931	Proto-oncogene tyrosine-protein kinase Src OS=Homo sapiens GN=SRC PE=1 SV=3 - [SRC_HUMAN]	28,36	7
P15498	Proto-oncogene vav OS=Homo sapiens GN=VAV1 PE=1 SV=4 - [VAV_HUMAN]	1,30	1
Q14242	P-selectin glycoprotein ligand 1 OS=Homo sapiens GN=SELPLG PE=1 SV=1 - [SELPL_HUMAN]	2,18	1
P16109	P-selectin OS=Homo sapiens GN=SELP PE=1 SV=3 - [LYAM3_HUMAN]	11,81	8
P00491	Purine nucleoside phosphorylase OS=Homo sapiens GN=PNP PE=1 SV=2 - [PNPH_HUMAN]	17,99	1

Q16740	Putative ATP-dependent Clp protease proteolytic subunit, mitochondrial OS=Homo sapiens GN=CLPP PE=1 SV=1 - [CLPP_HUMAN]	5,42	1
Q9Y315	Putative deoxyribose-phosphate aldolase OS=Homo sapiens GN=DERA PE=1 SV=2 - [DEOC_HUMAN]	5,03	1
A6NGU5	Putative gamma-glutamyltranspeptidase 3 OS=Homo sapiens GN=GGT3P PE=5 SV=2 - [GGT3_HUMAN]	2,11	1
Q14409	Putative glycerol kinase 3 OS=Homo sapiens GN=GK3P PE=5 SV=2 - [GLPK3_HUMAN]	1,81	1
Q8IZP2	Putative protein FAM10A4 OS=Homo sapiens GN=ST13P4 PE=5 SV=1 - [ST134_HUMAN]	4,17	1
P08559	Pyruvate dehydrogenase E1 component subunit alpha, somatic form, mitochondrial OS=Homo sapiens GN=PDHA1 PE=1 SV=3 - [O	7,44	3
P11177	Pyruvate dehydrogenase E1 component subunit beta, mitochondrial OS=Homo sapiens GN=PDHB PE=1 SV=3 - [ODPB_HUMAN]	6,13	2
O00330	Pyruvate dehydrogenase protein X component, mitochondrial OS=Homo sapiens GN=PDHX PE=1 SV=3 - [ODPX_HUMAN]	2,79	1
P14618	Pyruvate kinase PKM OS=Homo sapiens GN=PKM PE=1 SV=4 - [KPYM_HUMAN]	24,86	9
Q5HYI8	Rab-like protein 3 OS=Homo sapiens GN=RABL3 PE=1 SV=1 - [RABL3_HUMAN]	9,32	2
P35241	Radixin OS=Homo sapiens GN=RDX PE=1 SV=1 - [RADI_HUMAN]	15,95	4
Q14699	Raftlin OS=Homo sapiens GN=RFTN1 PE=1 SV=4 - [RFTN1_HUMAN]	3,63	2
Q6IAA8	Ragulator complex protein LAMTOR1 OS=Homo sapiens GN=LAMTOR1 PE=1 SV=2 - [LTOR1_HUMAN]	14,91	1
Q14644	Ras GTPase-activating protein 3 OS=Homo sapiens GN=RASA3 PE=1 SV=3 - [RASA3_HUMAN]	17,15	6
Q13576	Ras GTPase-activating-like protein IQGAP2 OS=Homo sapiens GN=IQGAP2 PE=1 SV=4 - [IQGA2_HUMAN]	2,54	2
Q15404	Ras suppressor protein 1 OS=Homo sapiens GN=RSU1 PE=1 SV=3 - [RSU1_HUMAN]	23,47	4
P63000	Ras-related C3 botulinum toxin substrate 1 OS=Homo sapiens GN=RAC1 PE=1 SV=1 - [RAC1_HUMAN]	34,90	4
P15153	Ras-related C3 botulinum toxin substrate 2 OS=Homo sapiens GN=RAC2 PE=1 SV=1 - [RAC2_HUMAN]	22,92	4
P61026	Ras-related protein Rab-10 OS=Homo sapiens GN=RAB10 PE=1 SV=1 - [RAB10_HUMAN]	46,00	7
Q15907	Ras-related protein Rab-11B OS=Homo sapiens GN=RAB11B PE=1 SV=4 - [RB11B_HUMAN]	46,33	6
P61106	Ras-related protein Rab-14 OS=Homo sapiens GN=RAB14 PE=1 SV=4 - [RAB14_HUMAN]	45,12	7
Q9NP72	Ras-related protein Rab-18 OS=Homo sapiens GN=RAB18 PE=1 SV=1 - [RAB18_HUMAN]	7,28	1
P62820	Ras-related protein Rab-1A OS=Homo sapiens GN=RAB1A PE=1 SV=3 - [RAB1A_HUMAN]	37,07	7
Q9H0U4	Ras-related protein Rab-1B OS=Homo sapiens GN=RAB1B PE=1 SV=1 - [RAB1B_HUMAN]	34,83	5
Q9UL25	Ras-related protein Rab-21 OS=Homo sapiens GN=RAB21 PE=1 SV=3 - [RAB21_HUMAN]	16,89	2
O00194	Ras-related protein Rab-27B OS=Homo sapiens GN=RAB27B PE=1 SV=4 - [RB27B_HUMAN]	48,62	7
P61019	Ras-related protein Rab-2A OS=Homo sapiens GN=RAB2A PE=1 SV=1 - [RAB2A_HUMAN]	24,06	2
Q8WUD1	Ras-related protein Rab-2B OS=Homo sapiens GN=RAB2B PE=1 SV=1 - [RAB2B_HUMAN]	31,94	3
Q15771	Ras-related protein Rab-30 OS=Homo sapiens GN=RAB30 PE=1 SV=2 - [RAB30_HUMAN]	19,21	3
Q13637	Ras-related protein Rab-32 OS=Homo sapiens GN=RAB32 PE=1 SV=3 - [RAB32_HUMAN]	32,00	5
Q14088	Ras-related protein Rab-33A OS=Homo sapiens GN=RAB33A PE=1 SV=2 - [RB33A_HUMAN]	8,86	2
Q15286	Ras-related protein Rab-35 OS=Homo sapiens GN=RAB35 PE=1 SV=1 - [RAB35_HUMAN]	15,42	2
Q96AX2	Ras-related protein Rab-37 OS=Homo sapiens GN=RAB37 PE=1 SV=3 - [RAB37_HUMAN]	28,25	4
P57729	Ras-related protein Rab-38 OS=Homo sapiens GN=RAB38 PE=1 SV=1 - [RAB38_HUMAN]	4,74	1
P20338	Ras-related protein Rab-4A OS=Homo sapiens GN=RAB4A PE=1 SV=3 - [RAB4A_HUMAN]	23,39	1
P61018	Ras-related protein Rab-4B OS=Homo sapiens GN=RAB4B PE=1 SV=1 - [RAB4B_HUMAN]	27,70	3
P20339	Ras-related protein Rab-5A OS=Homo sapiens GN=RAB5A PE=1 SV=2 - [RAB5A_HUMAN]	10,70	1
P61020	Ras-related protein Rab-5B OS=Homo sapiens GN=RAB5B PE=1 SV=1 - [RAB5B_HUMAN]	17,21	2
P51148	Ras-related protein Rab-5C OS=Homo sapiens GN=RAB5C PE=1 SV=2 - [RAB5C_HUMAN]	22,69	3
P20340	Ras-related protein Rab-6A OS=Homo sapiens GN=RAB6A PE=1 SV=3 - [RAB6A_HUMAN]	43,75	8

Q9NRW1	Ras-related protein Rab-6B OS=Homo sapiens GN=RAB6B PE=1 SV=1 - [RAB6B_HUMAN]	41,35	8
P51149	Ras-related protein Rab-7a OS=Homo sapiens GN=RAB7A PE=1 SV=1 - [RAB7A_HUMAN]	42,03	6
P61006	Ras-related protein Rab-8A OS=Homo sapiens GN=RAB8A PE=1 SV=1 - [RAB8A_HUMAN]	34,78	8
Q92930	Ras-related protein Rab-8B OS=Homo sapiens GN=RAB8B PE=1 SV=2 - [RAB8B_HUMAN]	41,55	8
P11233	Ras-related protein Ral-A OS=Homo sapiens GN=RALA PE=1 SV=1 - [RALA_HUMAN]	25,24	2
P11234	Ras-related protein Ral-B OS=Homo sapiens GN=RALB PE=1 SV=1 - [RALB_HUMAN]	27,67	3
P62834	Ras-related protein Rap-1A OS=Homo sapiens GN=RAP1A PE=1 SV=1 - [RAP1A_HUMAN]	30,43	5
P61224	Ras-related protein Rap-1b OS=Homo sapiens GN=RAP1B PE=1 SV=1 - [RAP1B_HUMAN]	37,50	7
P10114	Ras-related protein Rap-2a OS=Homo sapiens GN=RAP2A PE=1 SV=1 - [RAP2A_HUMAN]	16,39	1
P61225	Ras-related protein Rap-2b OS=Homo sapiens GN=RAP2B PE=1 SV=1 - [RAP2B_HUMAN]	39,34	3
P10301	Ras-related protein R-Ras OS=Homo sapiens GN=RRAS PE=1 SV=1 - [RRAS_HUMAN]	16,97	3
Q00765	Receptor expression-enhancing protein 5 OS=Homo sapiens GN=REEP5 PE=1 SV=3 - [REEP5_HUMAN]	15,34	2
P23467	Receptor-type tyrosine-protein phosphatase beta OS=Homo sapiens GN=PTPRB PE=1 SV=3 - [PTPRB_HUMAN]	5,06	2
P08575	Receptor-type tyrosine-protein phosphatase C OS=Homo sapiens GN=PTPRC PE=1 SV=2 - [PTPRC_HUMAN]	7,44	2
P23469	Receptor-type tyrosine-protein phosphatase epsilon OS=Homo sapiens GN=PTPRE PE=1 SV=1 - [PTPRE_HUMAN]	1,29	1
Q12913	Receptor-type tyrosine-protein phosphatase eta OS=Homo sapiens GN=PTPRJ PE=1 SV=3 - [PTPRJ_HUMAN]	13,91	11
O43665	Regulator of G-protein signaling 10 OS=Homo sapiens GN=RGS10 PE=1 SV=2 - [RGS10_HUMAN]	6,94	1
O75787	Renin receptor OS=Homo sapiens GN=ATP6AP2 PE=1 SV=2 - [RENR_HUMAN]	3,14	1
Q16799	Reticulon-1 OS=Homo sapiens GN=RTN1 PE=1 SV=1 - [RTN1_HUMAN]	4,38	1
O75298	Reticulon-2 OS=Homo sapiens GN=RTN2 PE=1 SV=1 - [RTN2_HUMAN]	7,89	2
O95197	Reticulon-3 OS=Homo sapiens GN=RTN3 PE=1 SV=2 - [RTN3_HUMAN]	2,62	2
Q9NQC3	Reticulon-4 OS=Homo sapiens GN=RTN4 PE=1 SV=2 - [RTN4_HUMAN]	6,12	5
Q9HB40	Retinoid-inducible serine carboxypeptidase OS=Homo sapiens GN=SCPEP1 PE=1 SV=1 - [RISC_HUMAN]	5,09	1
Q8TC12	Retinol dehydrogenase 11 OS=Homo sapiens GN=RDH11 PE=1 SV=2 - [RDH11_HUMAN]	29,25	6
P52565	Rho GDP-dissociation inhibitor 1 OS=Homo sapiens GN=ARHGDIA PE=1 SV=3 - [GDIR1_HUMAN]	14,22	1
P52566	Rho GDP-dissociation inhibitor 2 OS=Homo sapiens GN=ARHGDIB PE=1 SV=3 - [GDIR2_HUMAN]	18,91	2
Q8N392	Rho GTPase-activating protein 18 OS=Homo sapiens GN=ARHGAP18 PE=1 SV=3 - [RHG18_HUMAN]	2,87	1
P98171	Rho GTPase-activating protein 4 OS=Homo sapiens GN=ARHGAP4 PE=1 SV=2 - [RHG04_HUMAN]	1,06	1
Q13464	Rho-associated protein kinase 1 OS=Homo sapiens GN=ROCK1 PE=1 SV=1 - [ROCK1_HUMAN]	2,14	2
O75116	Rho-associated protein kinase 2 OS=Homo sapiens GN=ROCK2 PE=1 SV=4 - [ROCK2_HUMAN]	4,18	2
P84095	Rho-related GTP-binding protein RhoG OS=Homo sapiens GN=RHOG PE=1 SV=1 - [RHOG_HUMAN]	19,90	3
Q9P2E9	Ribosome-binding protein 1 OS=Homo sapiens GN=RRBP1 PE=1 SV=4 - [RRBP1_HUMAN]	2,62	3
Q96T51	RUN and FYVE domain-containing protein 1 OS=Homo sapiens GN=RUFY1 PE=1 SV=2 - [RUFY1_HUMAN]	4,24	3
Q8NBX0	Saccharopine dehydrogenase-like oxidoreductase OS=Homo sapiens GN=SCCPDH PE=1 SV=1 - [SCPDL_HUMAN]	15,85	2
Q14BN4	Sarcolemmal membrane-associated protein OS=Homo sapiens GN=SLMAP PE=1 SV=1 - [SLMAP_HUMAN]	9,18	6
P16615	Sarcoplasmic/endoplasmic reticulum calcium ATPase 2 OS=Homo sapiens GN=ATP2A2 PE=1 SV=1 - [AT2A2_HUMAN]	18,04	17
Q93084	Sarcoplasmic/endoplasmic reticulum calcium ATPase 3 OS=Homo sapiens GN=ATP2A3 PE=1 SV=2 - [AT2A3_HUMAN]	25,60	22
Q8WVM8	Sec1 family domain-containing protein 1 OS=Homo sapiens GN=SCFD1 PE=1 SV=4 - [SCFD1_HUMAN]	3,58	2
O15126	Secretory carrier-associated membrane protein 1 OS=Homo sapiens GN=SCAMP1 PE=1 SV=2 - [SCAM1_HUMAN]	9,76	2
O15127	Secretory carrier-associated membrane protein 2 OS=Homo sapiens GN=SCAMP2 PE=1 SV=2 - [SCAM2_HUMAN]	15,81	3

O14828	Secretory carrier-associated membrane protein 3 OS=Homo sapiens GN=SCAMP3 PE=1 SV=3 - [SCAM3_HUMAN]	6,92	2
P49908	Selenoprotein P OS=Homo sapiens GN=SEPP1 PE=1 SV=3 - [SEPP1_HUMAN]	2,62	1
Q9NVA2	Septin-11 OS=Homo sapiens GN=SEPT11 PE=1 SV=3 - [SEP11_HUMAN]	2,33	1
Q15019	Septin-2 OS=Homo sapiens GN=SEPT2 PE=1 SV=1 - [SEPT2_HUMAN]	4,71	1
P34897	Serine hydroxymethyltransferase, mitochondrial OS=Homo sapiens GN=SHMT2 PE=1 SV=3 - [GLYM_HUMAN]	8,33	3
O15269	Serine palmitoyltransferase 1 OS=Homo sapiens GN=SPTLC1 PE=1 SV=1 - [SPTC1_HUMAN]	2,96	1
O94804	Serine/threonine-protein kinase 10 OS=Homo sapiens GN=STK10 PE=1 SV=1 - [STK10_HUMAN]	0,83	1
Q9Y5S2	Serine/threonine-protein kinase MRCK beta OS=Homo sapiens GN=CDC42BPB PE=1 SV=2 - [MRCKB_HUMAN]	2,10	1
P30153	Serine/threonine-protein phosphatase 2A 65 kDa regulatory subunit A alpha isoform OS=Homo sapiens GN=PPP2R1A PE=1 SV=4 -	4,75	3
Q96HS1	Serine/threonine-protein phosphatase PGAM5, mitochondrial OS=Homo sapiens GN=PGAM5 PE=1 SV=2 - [PGAM5_HUMAN]	10,03	2
P62136	Serine/threonine-protein phosphatase PP1-alpha catalytic subunit OS=Homo sapiens GN=PPP1CA PE=1 SV=1 - [PP1A_HUMAN]	8,79	1
P36873	Serine/threonine-protein phosphatase PP1-gamma catalytic subunit OS=Homo sapiens GN=PPP1CC PE=1 SV=1 - [PP1G_HUMAN]	6,19	1
Q9NP81	Serine--tRNA ligase, mitochondrial OS=Homo sapiens GN=SARS2 PE=1 SV=1 - [SYSM_HUMAN]	1,35	1
P02787	Serotransferrin OS=Homo sapiens GN=TF PE=1 SV=3 - [TRFE_HUMAN]	42,26	14
P29508	Serpin B3 OS=Homo sapiens GN=SERPINB3 PE=1 SV=2 - [SPB3_HUMAN]	7,69	1
P02768	Serum albumin OS=Homo sapiens GN=ALB PE=1 SV=2 - [ALBU_HUMAN]	52,05	25
P35542	Serum amyloid A-4 protein OS=Homo sapiens GN=SAA4 PE=1 SV=2 - [SAA4_HUMAN]	8,46	1
P02743	Serum amyloid P-component OS=Homo sapiens GN=APCS PE=1 SV=2 - [SAMP_HUMAN]	16,14	1
O95810	Serum deprivation-response protein OS=Homo sapiens GN=SDPR PE=1 SV=3 - [SDPR_HUMAN]	27,29	7
P27169	Serum paraoxonase/arylesterase 1 OS=Homo sapiens GN=PON1 PE=1 SV=3 - [PON1_HUMAN]	15,77	4
Q9H299	SH3 domain-binding glutamic acid-rich-like protein 3 OS=Homo sapiens GN=SH3BGRL3 PE=1 SV=1 - [SH3L3_HUMAN]	10,75	1
Q9Y210	Short transient receptor potential channel 6 OS=Homo sapiens GN=TRPC6 PE=1 SV=1 - [TRPC6_HUMAN]	7,09	5
P45954	Short/branched chain specific acyl-CoA dehydrogenase, mitochondrial OS=Homo sapiens GN=ACADSB PE=1 SV=1 - [ACDSB_HUMA	13,66	4
P16219	Short-chain specific acyl-CoA dehydrogenase, mitochondrial OS=Homo sapiens GN=ACADS PE=1 SV=1 - [ACADS_HUMAN]	2,43	1
Q9HAT2	Sialate O-acetylesterase OS=Homo sapiens GN=SIAE PE=1 SV=1 - [SIAE_HUMAN]	7,46	1
A6NMB1	Sialic acid-binding Ig-like lectin 16 OS=Homo sapiens GN=SIGLEC16 PE=2 SV=3 - [SIG16_HUMAN]	1,46	1
Q9H9B4	Sideroflexin-1 OS=Homo sapiens GN=SFXN1 PE=1 SV=4 - [SFXN1_HUMAN]	4,66	1
Q9BWM7	Sideroflexin-3 OS=Homo sapiens GN=SFXN3 PE=1 SV=3 - [SFXN3_HUMAN]	2,80	1
Q99720	Sigma non-opioid intracellular receptor 1 OS=Homo sapiens GN=SIGMAR1 PE=1 SV=1 - [SGMR1_HUMAN]	3,59	1
P61009	Signal peptidase complex subunit 3 OS=Homo sapiens GN=SPCS3 PE=1 SV=1 - [SPCS3_HUMAN]	15,56	2
P08240	Signal recognition particle receptor subunit alpha OS=Homo sapiens GN=SRPR PE=1 SV=2 - [SRPR_HUMAN]	3,92	2
Q9Y5M8	Signal recognition particle receptor subunit beta OS=Homo sapiens GN=SRPRB PE=1 SV=3 - [SRPRB_HUMAN]	4,80	1
P51692	Signal transducer and activator of transcription 5B OS=Homo sapiens GN=STAT5B PE=1 SV=2 - [STA5B_HUMAN]	1,14	1
Q6IA17	Single Ig IL-1-related receptor OS=Homo sapiens GN=SIGIRR PE=1 SV=3 - [SIGIR_HUMAN]	2,93	1
Q04837	Single-stranded DNA-binding protein, mitochondrial OS=Homo sapiens GN=SSBP1 PE=1 SV=1 - [SSBP_HUMAN]	10,14	1
Q5T750	Skin-specific protein 32 OS=Homo sapiens GN=XP32 PE=1 SV=1 - [XP32_HUMAN]	3,20	1
Q9UIB8	SLAM family member 5 OS=Homo sapiens GN=CD84 PE=1 SV=1 - [SLAF5_HUMAN]	14,78	3
B2RUZ4	Small integral membrane protein 1 OS=Homo sapiens GN=SMIM1 PE=1 SV=1 - [SMIM1_HUMAN]	20,51	1
Q71RC9	Small integral membrane protein 5 OS=Homo sapiens GN=SMIM5 PE=4 SV=2 - [SMIM5_HUMAN]	11,69	1
Q9BSF0	Small membrane A-kinase anchor protein OS=Homo sapiens GN=C2orf88 PE=1 SV=2 - [SMAKA_HUMAN]	13,68	1

P61956	Small ubiquitin-related modifier 2 OS=Homo sapiens GN=SUMO2 PE=1 SV=3 - [SUMO2_HUMAN]	12,63	1
P53814	Smoothelin OS=Homo sapiens GN=SMTN PE=1 SV=7 - [SMTN_HUMAN]	2,29	2
P19634	Sodium/hydrogen exchanger 1 OS=Homo sapiens GN=SLC9A1 PE=1 SV=2 - [SL9A1_HUMAN]	4,66	1
Q8IVB4	Sodium/hydrogen exchanger 9 OS=Homo sapiens GN=SLC9A9 PE=1 SV=1 - [SL9A9_HUMAN]	9,46	1
P05023	Sodium/potassium-transporting ATPase subunit alpha-1 OS=Homo sapiens GN=ATP1A1 PE=1 SV=1 - [AT1A1_HUMAN]	25,22	12
P54709	Sodium/potassium-transporting ATPase subunit beta-3 OS=Homo sapiens GN=ATP1B3 PE=1 SV=1 - [AT1B3_HUMAN]	16,49	1
A1L4H1	Soluble scavenger receptor cysteine-rich domain-containing protein SSC5D OS=Homo sapiens GN=SSC5D PE=2 SV=3 - [SRCRL_HU	0,57	1
P11166	Solute carrier family 2, facilitated glucose transporter member 1 OS=Homo sapiens GN=SLC2A1 PE=1 SV=2 - [GTR1_HUMAN]	5,69	1
P11169	Solute carrier family 2, facilitated glucose transporter member 3 OS=Homo sapiens GN=SLC2A3 PE=1 SV=1 - [GTR3_HUMAN]	17,14	5
Q96BI1	Solute carrier family 22 member 18 OS=Homo sapiens GN=SLC22A18 PE=1 SV=3 - [S22AI_HUMAN]	2,83	1
Q5M8T2	Solute carrier family 35 member D3 OS=Homo sapiens GN=SLC35D3 PE=2 SV=1 - [S35D3_HUMAN]	2,88	1
Q9UIG8	Solute carrier organic anion transporter family member 3A1 OS=Homo sapiens GN=SLCO3A1 PE=1 SV=3 - [SO3A1_HUMAN]	1,55	1
Q99523	Sortilin OS=Homo sapiens GN=SORT1 PE=1 SV=3 - [SORT_HUMAN]	4,21	2
P02549	Spectrin alpha chain, erythrocytic 1 OS=Homo sapiens GN=SPTA1 PE=1 SV=5 - [SPTA1_HUMAN]	21,54	30
Q13813	Spectrin alpha chain, non-erythrocytic 1 OS=Homo sapiens GN=SPTAN1 PE=1 SV=3 - [SPTN1_HUMAN]	5,10	4
P11277	Spectrin beta chain, erythrocytic OS=Homo sapiens GN=SPTB PE=1 SV=5 - [SPTB1_HUMAN]	22,23	22
Q01082	Spectrin beta chain, non-erythrocytic 1 OS=Homo sapiens GN=SPTBN1 PE=1 SV=2 - [SPTB2_HUMAN]	7,78	13
P63208	S-phase kinase-associated protein 1 OS=Homo sapiens GN=SKP1 PE=1 SV=2 - [SKP1_HUMAN]	7,36	1
Q8WW59	SPRY domain-containing protein 4 OS=Homo sapiens GN=SPRYD4 PE=1 SV=2 - [SPRY4_HUMAN]	5,31	1
O75563	Src kinase-associated phosphoprotein 2 OS=Homo sapiens GN=SKAP2 PE=1 SV=1 - [SKAP2_HUMAN]	10,58	1
Q14247	Src substrate cortactin OS=Homo sapiens GN=CTTN PE=1 SV=2 - [SRC8_HUMAN]	4,91	3
Q9UJZ1	Stomatin-like protein 2, mitochondrial OS=Homo sapiens GN=STOML2 PE=1 SV=1 - [STML2_HUMAN]	16,57	4
Q8WXE9	Stonin-2 OS=Homo sapiens GN=STON2 PE=1 SV=1 - [STON2_HUMAN]	1,88	1
P38646	Stress-70 protein, mitochondrial OS=Homo sapiens GN=HSPA9 PE=1 SV=2 - [GRP75_HUMAN]	21,21	11
O43815	Striatin OS=Homo sapiens GN=STRN PE=1 SV=4 - [STRN_HUMAN]	5,00	3
Q13033	Striatin-3 OS=Homo sapiens GN=STRN3 PE=1 SV=3 - [STRN3_HUMAN]	2,26	2
Q9NRL3	Striatin-4 OS=Homo sapiens GN=STRN4 PE=1 SV=2 - [STRN4_HUMAN]	4,52	3
Q13586	Stromal interaction molecule 1 OS=Homo sapiens GN=STIM1 PE=1 SV=3 - [STIM1_HUMAN]	15,62	7
P31040	Succinate dehydrogenase [ubiquinone] flavoprotein subunit, mitochondrial OS=Homo sapiens GN=SDHA PE=1 SV=2 - [DHSA_HUM	1,66	1
Q9P2R7	Succinyl-CoA ligase [ADP-forming] subunit beta, mitochondrial OS=Homo sapiens GN=SUCLA2 PE=1 SV=3 - [SUCB1_HUMAN]	10,80	5
Q96I99	Succinyl-CoA ligase [GDP-forming] subunit beta, mitochondrial OS=Homo sapiens GN=SUCLG2 PE=1 SV=2 - [SUCB2_HUMAN]	2,55	1
P55809	Succinyl-CoA:3-ketoacid coenzyme A transferase 1, mitochondrial OS=Homo sapiens GN=OXCT1 PE=1 SV=1 - [SCOT1_HUMAN]	10,96	5
Q9Y6N5	Sulfide:quinone oxidoreductase, mitochondrial OS=Homo sapiens GN=SQRDL PE=1 SV=1 - [SQRD_HUMAN]	27,56	3
P04179	Superoxide dismutase [Mn], mitochondrial OS=Homo sapiens GN=SOD2 PE=1 SV=2 - [SODM_HUMAN]	23,87	4
Q6UWP8	Suprabasin OS=Homo sapiens GN=SBSN PE=2 SV=2 - [SBSN_HUMAN]	11,53	1
Q15526	Surfeit locus protein 1 OS=Homo sapiens GN=SURF1 PE=1 SV=1 - [SURF1_HUMAN]	9,00	1
Q6UWL2	Sushi domain-containing protein 1 OS=Homo sapiens GN=SUSD1 PE=1 SV=1 - [SUSD1_HUMAN]	4,82	2
Q7L0J3	Synaptic vesicle glycoprotein 2A OS=Homo sapiens GN=SV2A PE=1 SV=1 - [SV2A_HUMAN]	1,75	1
O43760	Synaptogyrin-2 OS=Homo sapiens GN=SYNGR2 PE=1 SV=1 - [SNG2_HUMAN]	8,04	1
O00161	Synaptosomal-associated protein 23 OS=Homo sapiens GN=SNAP23 PE=1 SV=1 - [SNP23_HUMAN]	41,23	6

O95721	Synaptosomal-associated protein 29 OS=Homo sapiens GN=SNAP29 PE=1 SV=1 - [SNP29_HUMAN]	23,26	2
Q96C24	Synaptotagmin-like protein 4 OS=Homo sapiens GN=SYTL4 PE=1 SV=2 - [SYTL4_HUMAN]	21,46	6
O75558	Syntaxin-11 OS=Homo sapiens GN=STX11 PE=2 SV=1 - [STX11_HUMAN]	42,16	9
Q86Y82	Syntaxin-12 OS=Homo sapiens GN=STX12 PE=1 SV=1 - [STX12_HUMAN]	12,32	3
O14662	Syntaxin-16 OS=Homo sapiens GN=STX16 PE=1 SV=3 - [STX16_HUMAN]	2,46	1
Q12846	Syntaxin-4 OS=Homo sapiens GN=STX4 PE=1 SV=2 - [STX4_HUMAN]	24,58	5
O15400	Syntaxin-7 OS=Homo sapiens GN=STX7 PE=1 SV=4 - [STX7_HUMAN]	23,75	4
Q15833	Syntaxin-binding protein 2 OS=Homo sapiens GN=STXBP2 PE=1 SV=2 - [STXB2_HUMAN]	31,87	11
O00186	Syntaxin-binding protein 3 OS=Homo sapiens GN=STXBP3 PE=1 SV=2 - [STXB3_HUMAN]	8,45	3
O00258	Tail-anchored protein insertion receptor WRB OS=Homo sapiens GN=WRB PE=1 SV=2 - [WRB_HUMAN]	5,17	1
Q9Y490	Talin-1 OS=Homo sapiens GN=TLN1 PE=1 SV=3 - [TLN1_HUMAN]	45,69	71
Q9Y4G6	Talin-2 OS=Homo sapiens GN=TLN2 PE=1 SV=4 - [TLN2_HUMAN]	5,70	10
Q8N9U0	Tandem C2 domains nuclear protein OS=Homo sapiens GN=TC2N PE=1 SV=2 - [TAC2N_HUMAN]	10,20	1
P78371	T-complex protein 1 subunit beta OS=Homo sapiens GN=CCT2 PE=1 SV=4 - [TCPB_HUMAN]	1,31	1
P50991	T-complex protein 1 subunit delta OS=Homo sapiens GN=CCT4 PE=1 SV=4 - [TCPD_HUMAN]	3,90	2
P48643	T-complex protein 1 subunit epsilon OS=Homo sapiens GN=CCT5 PE=1 SV=1 - [TCPE_HUMAN]	1,85	1
P49368	T-complex protein 1 subunit gamma OS=Homo sapiens GN=CCT3 PE=1 SV=4 - [TCPG_HUMAN]	2,02	1
P50990	T-complex protein 1 subunit theta OS=Homo sapiens GN=CCT8 PE=1 SV=4 - [TCPQ_HUMAN]	5,66	2
P24821	Tenascin OS=Homo sapiens GN=TNC PE=1 SV=3 - [TENA_HUMAN]	4,63	7
Q9Y6I9	Testis-expressed sequence 264 protein OS=Homo sapiens GN=TEX264 PE=1 SV=1 - [TX264_HUMAN]	3,19	1
Q8NG11	Tetraspanin-14 OS=Homo sapiens GN=TSPAN14 PE=1 SV=1 - [TSN14_HUMAN]	10,74	1
O60636	Tetraspanin-2 OS=Homo sapiens GN=TSPAN2 PE=2 SV=2 - [TSN2_HUMAN]	5,43	1
Q96QS1	Tetraspanin-32 OS=Homo sapiens GN=TSPAN32 PE=2 SV=1 - [TSN32_HUMAN]	7,81	1
Q86TV6	Tetratricopeptide repeat protein 7B OS=Homo sapiens GN=TTC7B PE=1 SV=3 - [TTC7B_HUMAN]	2,02	1
Q8NBS9	Thioredoxin domain-containing protein 5 OS=Homo sapiens GN=TXNDC5 PE=1 SV=2 - [TXND5_HUMAN]	4,40	1
P10599	Thioredoxin OS=Homo sapiens GN=TXN PE=1 SV=3 - [THIO_HUMAN]	31,43	1
Q9NNW7	Thioredoxin reductase 2, mitochondrial OS=Homo sapiens GN=TXNRD2 PE=1 SV=3 - [TRXR2_HUMAN]	2,10	1
P30048	Thioredoxin-dependent peroxide reductase, mitochondrial OS=Homo sapiens GN=PRDX3 PE=1 SV=3 - [PRDX3_HUMAN]	15,63	1
Q9H3N1	Thioredoxin-related transmembrane protein 1 OS=Homo sapiens GN=TMX1 PE=1 SV=1 - [TMX1_HUMAN]	17,86	3
Q9H1E5	Thioredoxin-related transmembrane protein 4 OS=Homo sapiens GN=TMX4 PE=1 SV=1 - [TMX4_HUMAN]	3,44	1
Q16762	Thiosulfate sulfurtransferase OS=Homo sapiens GN=TST PE=1 SV=4 - [THTR_HUMAN]	21,21	1
P07996	Thrombospondin-1 OS=Homo sapiens GN=THBS1 PE=1 SV=2 - [TSP1_HUMAN]	36,41	26
P21731	Thromboxane A2 receptor OS=Homo sapiens GN=TBXA2R PE=1 SV=3 - [TA2R_HUMAN]	3,21	1
P24557	Thromboxane-A synthase OS=Homo sapiens GN=TBXAS1 PE=1 SV=3 - [THAS_HUMAN]	25,89	9
P19971	Thymidine phosphorylase OS=Homo sapiens GN=TYMP PE=1 SV=2 - [TYPH_HUMAN]	9,96	3
Q9UDY2	Tight junction protein ZO-2 OS=Homo sapiens GN=TJP2 PE=1 SV=2 - [ZO2_HUMAN]	5,04	5
Q8NFQ8	Torsin-1A-interacting protein 2 OS=Homo sapiens GN=TOR1AIP2 PE=1 SV=1 - [TOIP2_HUMAN]	2,34	1
Q9NXH8	Torsin-4A OS=Homo sapiens GN=TOR4A PE=1 SV=2 - [TOR4A_HUMAN]	8,04	2
P37837	Transaldolase OS=Homo sapiens GN=TALDO1 PE=1 SV=2 - [TALDO_HUMAN]	8,31	2
Q00059	Transcription factor A, mitochondrial OS=Homo sapiens GN=TFAM PE=1 SV=1 - [TFAM_HUMAN]	4,07	1

P02786	Transferrin receptor protein 1 OS=Homo sapiens GN=TFRC PE=1 SV=2 - [TFR1_HUMAN]	14,47	7
P01137	Transforming growth factor beta-1 OS=Homo sapiens GN=TGFB1 PE=1 SV=2 - [TGFB1_HUMAN]	27,69	7
Q15582	Transforming growth factor-beta-induced protein ig-h3 OS=Homo sapiens GN=TGFBI PE=1 SV=1 - [BGH3_HUMAN]	2,49	1
P37802	Transgelin-2 OS=Homo sapiens GN=TAGLN2 PE=1 SV=3 - [TAGL2_HUMAN]	35,18	6
O43493	Trans-Golgi network integral membrane protein 2 OS=Homo sapiens GN=TGOLN2 PE=1 SV=2 - [TGON2_HUMAN]	10,63	4
Q8TD43	Transient receptor potential cation channel subfamily M member 4 OS=Homo sapiens GN=TRPM4 PE=1 SV=1 - [TRPM4_HUMAN]	0,82	1
P55072	Transitional endoplasmic reticulum ATPase OS=Homo sapiens GN=VCP PE=1 SV=4 - [TERA_HUMAN]	25,43	10
P29401	Transketolase OS=Homo sapiens GN=TKT PE=1 SV=3 - [TKT_HUMAN]	2,89	1
Q99442	Translocation protein SEC62 OS=Homo sapiens GN=SEC62 PE=1 SV=1 - [SEC62_HUMAN]	9,52	3
P51571	Translocon-associated protein subunit delta OS=Homo sapiens GN=SSR4 PE=1 SV=1 - [SSRD_HUMAN]	25,43	2
Q99805	Transmembrane 9 superfamily member 2 OS=Homo sapiens GN=TM9SF2 PE=1 SV=1 - [TM9S2_HUMAN]	7,99	1
O75069	Transmembrane and coiled-coil domains protein 2 OS=Homo sapiens GN=TMCC2 PE=1 SV=3 - [TMCC2_HUMAN]	1,13	1
Q9BVT8	Transmembrane and ubiquitin-like domain-containing protein 1 OS=Homo sapiens GN=TMUB1 PE=1 SV=1 - [TMUB1_HUMAN]	4,07	1
Q7Z403	Transmembrane channel-like protein 6 OS=Homo sapiens GN=TMC6 PE=2 SV=2 - [TMC6_HUMAN]	1,24	1
P49755	Transmembrane emp24 domain-containing protein 10 OS=Homo sapiens GN=TMED10 PE=1 SV=2 - [TMEDA_HUMAN]	25,57	3
Q15363	Transmembrane emp24 domain-containing protein 2 OS=Homo sapiens GN=TMED2 PE=1 SV=1 - [TMED2_HUMAN]	13,43	1
Q7Z7H5	Transmembrane emp24 domain-containing protein 4 OS=Homo sapiens GN=TMED4 PE=1 SV=1 - [TMED4_HUMAN]	8,81	1
Q9Y3B3	Transmembrane emp24 domain-containing protein 7 OS=Homo sapiens GN=TMED7 PE=1 SV=2 - [TMED7_HUMAN]	9,38	2
Q9BVK6	Transmembrane emp24 domain-containing protein 9 OS=Homo sapiens GN=TMED9 PE=1 SV=2 - [TMED9_HUMAN]	17,87	2
Q14956	Transmembrane glycoprotein NMB OS=Homo sapiens GN=GPNMB PE=1 SV=2 - [GPNMB_HUMAN]	1,57	1
Q9BVC6	Transmembrane protein 109 OS=Homo sapiens GN=TMEM109 PE=1 SV=1 - [TM109_HUMAN]	5,35	1
Q9HC07	Transmembrane protein 165 OS=Homo sapiens GN=TMEM165 PE=1 SV=1 - [TM165_HUMAN]	5,56	1
Q9H330	Transmembrane protein 245 OS=Homo sapiens GN=TMEM245 PE=1 SV=2 - [TM245_HUMAN]	1,43	1
P57088	Transmembrane protein 33 OS=Homo sapiens GN=TMEM33 PE=1 SV=2 - [TMM33_HUMAN]	14,98	1
Q8WWA1	Transmembrane protein 40 OS=Homo sapiens GN=TMEM40 PE=1 SV=2 - [TMM40_HUMAN]	12,45	2
Q9BTV4	Transmembrane protein 43 OS=Homo sapiens GN=TMEM43 PE=1 SV=1 - [TMM43_HUMAN]	27,25	1
O94886	Transmembrane protein 63A OS=Homo sapiens GN=TMEM63A PE=2 SV=3 - [TM63A_HUMAN]	5,95	1
Q5T3F8	Transmembrane protein 63B OS=Homo sapiens GN=TMEM63B PE=1 SV=1 - [TM63B_HUMAN]	1,08	1
Q9NQ34	Transmembrane protein 9B OS=Homo sapiens GN=TMEM9B PE=1 SV=1 - [TMM9B_HUMAN]	8,59	1
Q92973	Transportin-1 OS=Homo sapiens GN=TNPO1 PE=1 SV=2 - [TNPO1_HUMAN]	1,22	1
P02766	Transthyretin OS=Homo sapiens GN=TTR PE=1 SV=1 - [TTHY_HUMAN]	17,69	1
P53007	Tricarboxylate transport protein, mitochondrial OS=Homo sapiens GN=SLC25A1 PE=1 SV=2 - [TXTP_HUMAN]	6,43	1
P40939	Trifunctional enzyme subunit alpha, mitochondrial OS=Homo sapiens GN=HADHA PE=1 SV=2 - [ECHA_HUMAN]	14,68	9
P55084	Trifunctional enzyme subunit beta, mitochondrial OS=Homo sapiens GN=HADHB PE=1 SV=3 - [ECHB_HUMAN]	13,71	7
P60174	Triosephosphate isomerase OS=Homo sapiens GN=TPI1 PE=1 SV=3 - [TPIS_HUMAN]	14,69	4
Q8NG06	Tripartite motif-containing protein 58 OS=Homo sapiens GN=TRIM58 PE=2 SV=2 - [TRI58_HUMAN]	2,88	1
O14773	Tripeptidyl-peptidase 1 OS=Homo sapiens GN=TPP1 PE=1 SV=2 - [TPP1_HUMAN]	6,22	1
P29144	Tripeptidyl-peptidase 2 OS=Homo sapiens GN=TPP2 PE=1 SV=4 - [TPP2_HUMAN]	2,56	1
P28289	Tropomodulin-1 OS=Homo sapiens GN=TMOD1 PE=1 SV=1 - [TMOD1_HUMAN]	3,06	1
P06753	Tropomyosin alpha-3 chain OS=Homo sapiens GN=TPM3 PE=1 SV=2 - [TPM3_HUMAN]	18,95	3

P67936	Tropomyosin alpha-4 chain OS=Homo sapiens GN=TPM4 PE=1 SV=3 - [TPM4_HUMAN]	42,34	7
P07951	Tropomyosin beta chain OS=Homo sapiens GN=TPM2 PE=1 SV=1 - [TPM2_HUMAN]	21,83	5
Q9BQE3	Tubulin alpha-1C chain OS=Homo sapiens GN=TUBA1C PE=1 SV=1 - [TBA1C_HUMAN]	26,50	6
P68366	Tubulin alpha-4A chain OS=Homo sapiens GN=TUBA4A PE=1 SV=1 - [TBA4A_HUMAN]	22,10	5
Q9NY65	Tubulin alpha-8 chain OS=Homo sapiens GN=TUBA8 PE=1 SV=1 - [TBA8_HUMAN]	21,83	6
P07437	Tubulin beta chain OS=Homo sapiens GN=TUBB PE=1 SV=2 - [TBB5_HUMAN]	31,76	10
Q9H4B7	Tubulin beta-1 chain OS=Homo sapiens GN=TUBB1 PE=1 SV=1 - [TBB1_HUMAN]	37,69	11
P68371	Tubulin beta-4B chain OS=Homo sapiens GN=TUBB4B PE=1 SV=1 - [TBB4B_HUMAN]	28,31	8
O43399	Tumor protein D54 OS=Homo sapiens GN=TPD52L2 PE=1 SV=2 - [TPD54_HUMAN]	5,34	1
Q8N4L2	Type 2 phosphatidylinositol 4,5-bisphosphate 4-phosphatase OS=Homo sapiens GN=TMEM55A PE=1 SV=1 - [TM55A_HUMAN]	8,17	1
Q14642	Type I inositol 1,4,5-trisphosphate 5-phosphatase OS=Homo sapiens GN=INPP5A PE=1 SV=1 - [I5P1_HUMAN]	17,96	4
Q6RW13	Type-1 angiotensin II receptor-associated protein OS=Homo sapiens GN=AGTRAP PE=1 SV=1 - [ATRAP_HUMAN]	18,87	2
Q06187	Tyrosine-protein kinase BTK OS=Homo sapiens GN=BTK PE=1 SV=3 - [BTK_HUMAN]	1,52	1
P06241	Tyrosine-protein kinase Fyn OS=Homo sapiens GN=FYN PE=1 SV=3 - [FYN_HUMAN]	12,29	3
P23458	Tyrosine-protein kinase JAK1 OS=Homo sapiens GN=JAK1 PE=1 SV=2 - [JAK1_HUMAN]	1,30	1
P07948	Tyrosine-protein kinase Lyn OS=Homo sapiens GN=LYN PE=1 SV=3 - [LYN_HUMAN]	32,42	8
P18031	Tyrosine-protein phosphatase non-receptor type 1 OS=Homo sapiens GN=PTPN1 PE=1 SV=1 - [PTN1_HUMAN]	2,99	1
Q8NBM4	Ubiquitin-associated domain-containing protein 2 OS=Homo sapiens GN=UBAC2 PE=2 SV=1 - [UBAC2_HUMAN]	5,23	1
P61088	Ubiquitin-conjugating enzyme E2 N OS=Homo sapiens GN=UBE2N PE=1 SV=1 - [UBE2N_HUMAN]	13,82	2
P22314	Ubiquitin-like modifier-activating enzyme 1 OS=Homo sapiens GN=UBA1 PE=1 SV=3 - [UBA1_HUMAN]	2,46	2
Q92575	UBX domain-containing protein 4 OS=Homo sapiens GN=UBXN4 PE=1 SV=2 - [UBXN4_HUMAN]	2,76	1
Q9NYU2	UDP-glucose:glycoprotein glucosyltransferase 1 OS=Homo sapiens GN=UGGT1 PE=1 SV=3 - [UGGG1_HUMAN]	11,19	6
P30085	UMP-CMP kinase OS=Homo sapiens GN=CMPK1 PE=1 SV=3 - [KCY_HUMAN]	6,12	1
Q86XI8	Uncharacterized protein C19orf68 OS=Homo sapiens GN=C19orf68 PE=1 SV=2 - [CS068_HUMAN]	1,59	1
Q9H425	Uncharacterized protein C1orf198 OS=Homo sapiens GN=C1orf198 PE=1 SV=1 - [CA198_HUMAN]	6,73	1
Q2LD37	Uncharacterized protein KIAA1109 OS=Homo sapiens GN=KIAA1109 PE=1 SV=2 - [K1109_HUMAN]	0,28	1
O00159	Unconventional myosin-Ic OS=Homo sapiens GN=MYO1C PE=1 SV=4 - [MYO1C_HUMAN]	3,86	1
Q9Y4I1	Unconventional myosin-Va OS=Homo sapiens GN=MYO5A PE=1 SV=2 - [MYO5A_HUMAN]	2,05	3
Q969H8	UPF0556 protein C19orf10 OS=Homo sapiens GN=C19orf10 PE=1 SV=1 - [CS010_HUMAN]	8,67	1
Q96IX5	Up-regulated during skeletal muscle growth protein 5 OS=Homo sapiens GN=USMG5 PE=1 SV=1 - [USMG5_HUMAN]	25,86	1
Q16851	UTP--glucose-1-phosphate uridylyltransferase OS=Homo sapiens GN=UGP2 PE=1 SV=5 - [UGPA_HUMAN]	3,35	2
P46939	Utrophin OS=Homo sapiens GN=UTRN PE=1 SV=2 - [UTRO_HUMAN]	0,41	1
Q3ZAQ7	Vacuolar ATPase assembly integral membrane protein VMA21 OS=Homo sapiens GN=VMA21 PE=1 SV=1 - [VMA21_HUMAN]	11,88	1
Q96RL7	Vacuolar protein sorting-associated protein 13A OS=Homo sapiens GN=VPS13A PE=1 SV=2 - [VP13A_HUMAN]	1,01	2
Q9UK41	Vacuolar protein sorting-associated protein 28 homolog OS=Homo sapiens GN=VPS28 PE=1 SV=1 - [VPS28_HUMAN]	4,52	1
Q9H9H4	Vacuolar protein sorting-associated protein 37B OS=Homo sapiens GN=VPS37B PE=1 SV=1 - [VP37B_HUMAN]	6,32	1
P50552	Vasodilator-stimulated phosphoprotein OS=Homo sapiens GN=VASP PE=1 SV=3 - [VASP_HUMAN]	23,16	7
Q8IWB7	WD repeat and FYVE domain-containing protein 1 OS=Homo sapiens GN=WDFY1 PE=1 SV=1 - [WDFY1_HUMAN]	2,68	1
O75083	WD repeat-containing protein 1 OS=Homo sapiens GN=WDR1 PE=1 SV=4 - [WDR1_HUMAN]	12,87	2
Q5JSH3	WD repeat-containing protein 44 OS=Homo sapiens GN=WDR44 PE=1 SV=1 - [WDR44_HUMAN]	3,40	3

P49748	Very long-chain specific acyl-CoA dehydrogenase, mitochondrial OS=Homo sapiens GN=ACADVL PE=1 SV=1 - [ACADV_HUMAN]	13,59	3
Q9P035	Very-long-chain (3R)-3-hydroxyacyl-[acyl-carrier protein] dehydratase 3 OS=Homo sapiens GN=PTPLAD1 PE=1 SV=2 - [HACD3_HU	6,91	1
Q12981	Vesicle transport protein SEC20 OS=Homo sapiens GN=BNIP1 PE=1 SV=3 - [SEC20_HUMAN]	3,95	1
Q9NZ43	Vesicle transport protein USE1 OS=Homo sapiens GN=USE1 PE=1 SV=2 - [USE1_HUMAN]	4,63	1
Q9UEU0	Vesicle transport through interaction with t-SNAREs homolog 1B OS=Homo sapiens GN=VTI1B PE=1 SV=3 - [VTI1B_HUMAN]	18,53	4
Q15836	Vesicle-associated membrane protein 3 OS=Homo sapiens GN=VAMP3 PE=1 SV=3 - [VAMP3_HUMAN]	40,00	3
O95183	Vesicle-associated membrane protein 5 OS=Homo sapiens GN=VAMP5 PE=1 SV=1 - [VAMP5_HUMAN]	17,24	1
P51809	Vesicle-associated membrane protein 7 OS=Homo sapiens GN=VAMP7 PE=1 SV=3 - [VAMP7_HUMAN]	13,18	1
Q9BV40	Vesicle-associated membrane protein 8 OS=Homo sapiens GN=VAMP8 PE=1 SV=1 - [VAMP8_HUMAN]	27,00	2
Q9P0L0	Vesicle-associated membrane protein-associated protein A OS=Homo sapiens GN=VAPA PE=1 SV=3 - [VAPA_HUMAN]	10,04	2
O95292	Vesicle-associated membrane protein-associated protein B/C OS=Homo sapiens GN=VAPB PE=1 SV=3 - [VAPB_HUMAN]	17,70	3
O75396	Vesicle-trafficking protein SEC22b OS=Homo sapiens GN=SEC22B PE=1 SV=4 - [SC22B_HUMAN]	33,02	7
Q12907	Vesicular integral-membrane protein VIP36 OS=Homo sapiens GN=LMAN2 PE=1 SV=1 - [LMAN2_HUMAN]	19,10	4
P18206	Vinculin OS=Homo sapiens GN=VCL PE=1 SV=4 - [VINC_HUMAN]	54,50	43
Q9Y6W5	Wiskott-Aldrich syndrome protein family member 2 OS=Homo sapiens GN=WASF2 PE=1 SV=3 - [WASF2_HUMAN]	9,44	2
P42768	Wiskott-Aldrich syndrome protein OS=Homo sapiens GN=WAS PE=1 SV=4 - [WASP_HUMAN]	15,14	4
P02774	Vitamin D-binding protein OS=Homo sapiens GN=GC PE=1 SV=1 - [VTDB_HUMAN]	6,96	3
P07225	Vitamin K-dependent protein S OS=Homo sapiens GN=PROS1 PE=1 SV=1 - [PROS_HUMAN]	13,46	2
P04004	Vitronectin OS=Homo sapiens GN=VTN PE=1 SV=1 - [VTNC_HUMAN]	13,81	2
P21796	Voltage-dependent anion-selective channel protein 1 OS=Homo sapiens GN=VDAC1 PE=1 SV=2 - [VDAC1_HUMAN]	48,06	6
P45880	Voltage-dependent anion-selective channel protein 2 OS=Homo sapiens GN=VDAC2 PE=1 SV=2 - [VDAC2_HUMAN]	45,24	6
Q9Y277	Voltage-dependent anion-selective channel protein 3 OS=Homo sapiens GN=VDAC3 PE=1 SV=1 - [VDAC3_HUMAN]	47,70	4
P04275	von Willebrand factor OS=Homo sapiens GN=VWF PE=1 SV=4 - [VWF_HUMAN]	23,21	36
Q93050	V-type proton ATPase 116 kDa subunit a isoform 1 OS=Homo sapiens GN=ATP6V0A1 PE=1 SV=3 - [VPP1_HUMAN]	14,22	5
Q9Y487	V-type proton ATPase 116 kDa subunit a isoform 2 OS=Homo sapiens GN=ATP6V0A2 PE=1 SV=2 - [VPP2_HUMAN]	6,78	2
P38606	V-type proton ATPase catalytic subunit A OS=Homo sapiens GN=ATP6V1A PE=1 SV=2 - [VATA_HUMAN]	2,43	1
Q6PML9	Zinc transporter 9 OS=Homo sapiens GN=SLC30A9 PE=1 SV=1 - [ZNT9_HUMAN]	1,58	1
P25311	Zinc-alpha-2-glycoprotein OS=Homo sapiens GN=AZGP1 PE=1 SV=2 - [ZA2G_HUMAN]	38,59	7
Q15942	Zyxin OS=Homo sapiens GN=ZYX PE=1 SV=1 - [ZYX_HUMAN]	6,64	3
